# Supplementary material for: Common data models to streamline metabolomics processing and annotation, and implementation in a Python pipeline
Source: PLoS Comput Biol. 2024 Jun 6;20(6):e1011912. doi: 10.1371/journal.pcbi.1011912 (PMC11185459; doi:10.1371/journal.pcbi.1011912)
Supplement: S1 File — (PDF) [file pcbi.1011912.s003.pdf]

Supplemental Material for

Common data models to streamline metabolomics processing and annotation, and implementation in a Python pipeline

Joshua M. Mitchell[1], Yuanye Chi[1], Maheshwor Thapa[1], Zhiqiang Pang[2], Jianguo Xia[2], Shuzhao Li[1,3]

[1]The Jackson Laboratory for Genomic Medicine, 10 Discovery Drive, Farmington, Connecticut , USA

[2]Institute of Parasitology, McGill University, Montreal, Quebec, Canada

[3]University of Connecticut School of Medicine, Farmington, Connecticut , USA

Dataset Descriptions

Five datasets generated in our own laboratory and three public datasets were used for the evaluation of this pipeline. Two sets of 1685 MS^1 acquisitions were acquired from human plasma samples in a vaccine study (HZV029) as well as various blanks and quality control samples. Each acquisition was acquired at 120,000K resolving power on a Thermo Scientific Orbitrap ID-X mass spectrometer equipped with a HILIC or RP guard and analytical column in positive or negative ionization mode respectively and a five-minute chromatography time (see LC-MS 5-minute method described below). More detailed methods on the preparation of these extracts and their analyses have been previously published [1]. Both have a corresponding set of MS^2 spectra collected using the AcquireX methodology on pooled study samples. These are referred to as “HZV029 Plasma RP-” and “HZV029 Plasma HILIC+” datasets respectively.

A subset of these HZV029 plasma samples was also analyzed using a different two-phase extraction protocol and a longer chromatography method (see standard method below). This dataset is referred to as the “HZV029 Two-Phase HILIC-” dataset and includes 69 MS^1 acquisitions which also includes blanks and quality controls. All these datasets were collected on the same ID-X mass spectrometer using the same chromatography system.

The “HZV029 QC” dataset is a subset of "HZV029 Plasma HILIC+" data, comprised of 268 acquisitions collected from two human pooled plasma samples collected repeatedly over 17 batches on the same ID-X mass spectrometer as the previously mentioned dataset using the 5-

minute method in positive ionization mode with a HILIC column. This dataset was previously deposited in Metabolomics Workbench as Project PR001423.

The three public datasets used here are: the Bowen 2023 dataset [2] consists of HILIC+ acquisition derived from cardiomyocyte cell pellets and media that have been treated with sunitinib (20 and 21 acquisitions respectively, excluding MSMS samples and QC samples but including blanks). This dataset was downloaded from Metabolights (accession code MTBLS2746). The Ansone 2021 dataset [3] consists of 105 acquisitions collected on extracts from plasma of patients with acute-severe COVID, post-acute COVID, or health non-COVID controls and was downloaded from Metabolights (accession code MTBLS3852). The checkmate study was downloaded from Metabolomics Workbench (project ID: PR000828, study ID: ST001237) and consists of 1221 samples.

For comparisons between MetaboAnalystR and pcpfm, subsets of the HZV029 Plasma RP-, HZV029 HILIC+ and checkmate studies were used. For the HZV029 datasets 85 samples were selected at random, 5 from each batch. Only study samples were considered for inclusion (i.e., no blanks, pooled samples, etc.). For the checkmate study, 50 samples were selected at random.

Plasma Two-Phase Preparation

Plasma samples were extracted following an in-house two-phase extraction protocol using methyl tert-butyl ether (MTBE) and methanol as a non-polar and polar extraction solvent and water as a phase separation solvent. The polar phase was used for metabolomics and non-polar phase for lipidomics studies. The MTBE method was introduced by Matayash 2008 [4] which we have modified and optimized. Briefly, 150 µl of ice-cold methanol and 300 µl of ice-cold MTBE was added to 20 µl of plasma to extract polar and non-polar lipid molecules, respectively. For a phase separation, 150 µl of ice-cold water was used. 4 µl of IS solution prepared by mixing 0.12 µl of 1 M D-Glucose-$^{13}C_6$, 0.2 mL of 5 mM Caffeine-3-methyl-^{13}C, 0.6 mL of 10 mM L-Methionine-$^{13}C_5$, 0.6 mL of 20 mM L-Glutamic acid-$^{13}C_5$, 0.8 µl of 10 mM Uracil-$^{15}N_2$, 4 mL of 2 mM L-Tyrosine-^{15}N and 3.68 mL water in 15-mL tube was added in each sample as spike-in controls for polar phase extraction. 10 µl of stable isotope labeled standards (Avanti SPLASH Lipidomix), representing differing lipid classes, were added to each sample to use as a spike ins quality control for non-polar phase extraction. All samples were vortexed (Vortex-Genie 2, Scientific Industries, cat. no.

SI-0236) and incubated with shaking (Eppendorf Thermo Mixer C) at 1000 rpm for 20 min at 4 °C followed by centrifugation at 4 °C for 15 min at 20,817 × g (Centrifuge 5430 R, Eppendorf). 100 µl of lower polar phase for metabolomics were transferred to 1.5 mL autosampler vial and 3 µl injected directly into UHPLC-MS. 250 µl of upper non-polar MTBE phase was transferred to new tube and dried for 2 hrs at Labconco CentriVap Centrifugal Vacuum Concentrator (CentriVap Benchtop Centrifugal Vacuum Concentrator with acrylic lid, Labconco Corporation, cat. no. 7810010), followed by resuspending in 70 µl of methanol:toulene (8:1, v/v) solution. 3 µl of reconstituted solution was injected into UHPLC-MS. For quality control (QC) and assurance (QA), 10 µl of methanol extract from each sample of batch 1 was collected and pooled together to prepare QC sample for polar phase metabolomics. Similarly, 10 µl of reconstituted methanol:toluene (8:1, v/v) solution from each sample of batch 1 was collected and pooled together to prepare QC sample to be used for non-polar phase lipidomics.

LC-MS 5-minute Method

The chromatographic separations were performed using Thermo Scientific Transcend Duo LX-2 UHPLC system interfaced with high resolution Thermo Scientific Orbitrap ID-X Tribid mass spectrometer with a HESI ionization source, using positive and negative ionization modes. All samples were maintained at 4 °C in the autosampler. Data were acquired using hydrophilic interaction liquid chromatography (HILIC) and reversed phase (RP) column in parallel both in positive and negative polarities in full scan mode with mass resolution of 120,000. An Accucore−150-Amide HILIC column (2.6 µm, 2.1 mm × 50 mm) embedded with Accucore-150-Amide-HILIC guard column (10 × 2.1 mm, 2.6 µm) (Thermo Fisher Scientific, MA, USA. Cat. 16726-012105) and a Hypersil GOLD RP column (3 µm, 2.1 mm × 50 mm) embedded with Hypersil GOLD RP guard column (2.1 mm x 10 mm, 3 µm) (Thermo Fisher Scientific, MA, USA. Cat. 25003-012101) maintained at 45 °C were used for chromatographic separation. 10 mM ammonium acetate in acetonitrile:water (95:5, v/v) with 0.1% acetic acid as mobile phase A and 10 mM ammonium acetate in acetonitrile:water (50:50, v/v) with 0.1% acetic acid as mobile phase B were used for HILIC method. 0.1% formic acid in water and 0.1% formic acid in acetonitrile were used as mobile phase A and B respectively for RP acquisition. For HILIC acquisition, following gradient was applied at a flow rate of 0.55 ml/min: 0–0.1 min: 0% B, 0.10–5.0 min: 98% B, and 5 min for cleaning and equilibration of column. For RP column, following gradient was applied at a flow rate of 0.4 ml/min: 0–0.1 min: 0% B, 0.10–1.9 min: 60% B, 1.9–5.0 min: 98% B, and 5 min cleaning and column equilibration. This way the mass spec data for each sample was

collected consecutively, carrying only one (either HILIC or RP) eluent to the MS for 5 min, while the other eluent was directed to the waste during washing and re-equilibration.

Mass spectrometry data were collected with the following MS settings: mass range, 80–1000 m/z; spray voltage, 3500 V (ESI +), 2800 V (ESI−); sheath gas, 45 Arb; auxiliary gas, 20 Arb; sweep gas, 1 Arb; ion transfer tube temperature, 325 °C; vaporizer temperature, 325 °C; full scan mass resolution, 120,000 (MS^1); normalized AGC target (%), 25; maximum injection time, 100 ms. Data dependent fragmentation (dd-MS/MS) parameters for each polarity as follows: isolation window (m/z), 1.2; stepped HCD collision energy (%), 20,40,80; dd-MS/MS resolution, 30,000; normalized AGC target (%), 20; maximum injection time (ms), 54; microscan, 1; cycle time (sec), 1.2. A full scan data-dependent MS^2 (ddMS2) method was utilized to collect MS^2 spectra for identification of compounds.

LC-MS "Standard" Method

The chromatographic separations were performed using Thermo Scientific Transcend Duo LX-2 UHPLC system interfaced with high resolution Thermo Scientific Orbitrap ID-X Tribid mass spectrometer with a HESI ionization source, using both positive and negative ionization mode with a run time of 8.5 min for polar and 12 min for non-polar extract. All samples were maintained at 4 °C in the autosampler. Data were acquired for polar and non-polar extract using HILIC and RP column, respectively in full scan mode with mass resolution of 60,000. A Hypersil GOLD RP column (3 mm, 2.1 mm x 50 mm) embedded with Hypersil GOLD RP guard column (2.1 mm x 10 mm, 3 µm) (Thermo Fisher Scientific, MA, USA. Cat. 25003-012101) maintained at 45 ºC was used for chromatographic separation of non-polar extract. An Accucore-150-Amide HILIC column (2.6 mm, 2.1 mm x 100 mm) embedded with Accucore-150-Amide-HILIC guard column (10 × 2.1 mm, 2.6 µm) (Thermo Fisher Scientific, MA, USA. Cat. 16726-012105) was used for polar extract. 10 mM ammonium acetate in acetonitrile:water (95:5, v/v) with 0.1% acetic acid as mobile phase A and 10 mM ammonium acetate in acetonitrile:water (50:50, v/v) with 0.1% acetic acid as mobile phase B were used for HILIC method. For HILIC acquisition, following gradient was applied at a flow rate of 0.55 ml/min: 0-0.2 min: 0% B, 0.20-8.75 min: 98% B, and 11.25 min for cleaning and equilibration of column. 10 mM ammonium formate in acetornitrile:water (60:40, v/v) and 10 mM ammonium formate in 2-propanolol:acetonitrile (90:10, v/v) with 0.1% formic acid were used as mobile phase A and B, respectively for RP positive acquisition. For RP negative, 10 mM ammonium acetate in acetonitrile: water (60:40, v/v) and 10 mM ammonium acetate in 2-propanol: acetonitrile (90:10, v/v) were used as mobile phase A and B respectively were used. For RP

column, following gradient was applied at a flow rate of 0.4 ml/min: 0-0.1 min: 15% B, 0.10-2.01 min: 30% B, 2.01-11.0 min: 82% B, 11.0-11.5 min: 99% B, 11.5-12.0 min: 99% B and 8 min cleaning and column equilibration.

Mass spectrometry data were collected with the following MS settings: mass range, 100-1700 m/z for lipidomics and 60-1000 for metabolomics; spray voltage, 3200 V (ESI+), 2800 V (ESI-); sheath gas, 45 Arb; auxiliary gas, 20 Arb; sweep gas, 1 Arb; ion transfer tube temperature, 325 °C; vaporizer temperature, 325 °C; full scan mass resolution, 60,000 (MS^1); normalized AGC target (%), 25; maximum injection time, 100 ms. Data dependent fragmentation (dd-MS/MS) parameters for each polarity as follows: isolation window (m/z), 1.2; stepped HCD collision energy (%), 20,40,80; dd-MS/MS resolution, 30,000; normalized AGC target (%), 20; maximum injection time (ms), 54; micro scan, 1; cycle time (sec), 1.2. A full scan data-dependent MS^2 (ddMS2) method was utilized to collect MS^2 spectra for identification of compounds.

AcquireX deep scan analysis of pooled plasma sample

AcquireX deep scan workflow was used to collect MS^2 spectra on pooled plasma sample. A solvent blank was used to detect unimportant precursor features and add them in exclusion list as a function of retention time and ion intensity. This is followed by analysis of a representative study sample to map all important precursor features and add them in an inclusion list. The workflow manages replicate sample analysis and automated creation of exclusion and inclusion lists with dynamic modification of those inclusion and exclusion lists in between each replicate injection. If the precursor ion intensity is greater in the sample by a user-defined parameter, acquisition of tandem MS is performed.

Regular full scan method was used to generate exclusion and inclusion list with the following MS settings: mass range, 100-1700 m/z for lipidomics and 60-1000 for metabolomics; spray voltage, 3200 V (ESI+), 2800 V (ESI-); sheath gas, 45 Arb; auxiliary gas, 20 Arb; sweep gas, 1 Arb; ion transfer tube temperature, 325 °C; vaporizer temperature, 325 °C; full scan mass resolution, 60,000 (MS^1); normalized AGC target (%), 25; maximum injection time, 100 ms. A full scan data-dependent MS^2 ($ddMS^2$) method was utilized to collect MS^2 spectra for identification of compounds with the following MS settings: isolation window (m/z), 1.2; stepped HCD collision energy (%), 20,40,80; dd-MS/MS resolution, 30,000; normalized AGC target (%), 20; maximum injection time (ms), 54; micro scan, 1; cycle time (sec), 1.2.

MS^2 Annotation and Comparison Methods

MS^2 annotations were generated for both the HZV029 HILIC+ and RP- datasets using the AcquireX Deep Scan data. Both Compound Discover (CD) and the pcpfm were used to generate annotations and similar settings were used for both analyses. Compound Discoverer was configured to annotate peaks with a minimum of 5 scans and a minimum intensity of 10,000. A precursor ion m/z tolerance and a fragment mass tolerance of 10ppm. The HighChem HighRes search algorithm with a match factor cutoff of 50 and an RT tolerance of 30 seconds was used for all MS^2 comparisons. Compound Discoverer method files are provided in the github repo.

Unlike pcpfm annotations which are natively mapped to their corresponding feature in the Asari feature table, CD annotations are reported relative to an internal CD annotation table and thus a second mapping step is necessary for the comparison of CD and pcpfm MS2 annotations. For CD annotations, an m/z tolerance of 10 ppm and a retention time tolerance of 30 seconds was used to map CD annotations to features in the corresponding Asari feature table.

For the pcpfm, MS^2 spectra are extracted from both experimental data and provided annotation databases using the import function in MatchMS for the provided filetype. Spectra were first mapped to features from the full feature table, i.e., blanks, QC samples, etc. without peak quality metrics via the empirical compounds constructed therefrom, using a 30 second retention time tolerance and an effective m/z tolerance of 10 ppm. MS^2 spectra are subjected to the default_filter function from MatchMS followed by intensity normalization. MS^2 similarity is calculated using the ConsineHungarian function from MatchMS. This function requires an absolute, not relative, mass tolerance and a value calculated using the precursor ion m/z and a 10ppm m/z tolerance was used for all comparisons. Comparisons with a score greater than 0.50, sharing at least one peak, and with precursor ions within 10ppm were considered a match and used for annotation.

To compare the annotation results for both the mapped CD and pcpfm annotations, annotated feature sets were constructed by concatenating the annotation's name with the id_number of the mapped feature (e.g., Caffeine_F1234). These two sets were then compared using Python's set logic operators to compare the annotations. This ensures that only identically named

annotations to the same asari feature are considered matches between the two methods. Furthermore, duplicate annotated features are removed in this process to minimize the impact of redundant identically named annotations that arise because of multiple MS^2 reference spectra per compound in MoNA.

Computational Platforms Used for Testing

Computational performance comparisons between the pcpfm and MetaboAnalystR were performed on an Intel NUC (model no. BOXNUC8i7BEH1) equipped with a 4-core, 8-thread Intel Core i7-8559U, 32gb of DDR4 RAM, a 256Gb SATA SSD and a 1TB HDD (all data was stored and processed on the SSD) with a fresh install of Ubuntu 20.04. R version 4.3.2, MetaboAnalyst4.0, and OptiLCMSv1.1.0 was installed along with Python3.10 and pcpfm v1.0.13.

All other analyses were performed using pcpfm v1.0.13 on a 14-inch 2023 MacBook Pro (model number MPHG3LL/A) equipped with an Apple M2 Max CPU (12 cores, 8 performance and 4 efficiency), 32 GB of RAM, and 1 TB AP1024Z NVMe SSD running macOS Ventura 13.6.2. For all performance evaluations the machine was powered via an AC adapter and low power mode was disabled. Basic functionality, including installing and completing the standard workflow on the HZV029 QC dataset , was tested on other platforms including x86-64 systems equipped with both AMD and Intel CPUs running Debian GNU/Linux 12 (bookworm) and Ubuntu 20.04 respectively. Software versions for the analyses were as follows: scipy [5] v1.10.1, gdown v.4.7.1 [6], matplotlib v3.8.2 [7], pycombat v0.20 [8], scikit-learn v1.3.2[9, 10], jms-metabolite-services v0.5.7, combat v0.3.3, matchms v0.23.1 [11], mass2chem v0.4.9, pymzml v2.5.2 [12], intervaltree v3.1.0 mass2chem, khipu-metabolomics v0.7.5, seaborn v0.13.0 [13], setuptools v69.0.2, metDataModel v0.6.1, fpdf v1.7.2 [14], asari-metabolomics v1.12.8 [15], numpy v1.24.4 [16], pandas 2.1.3 [17], and the python version was 3.9.6. Compound Discover was version 3.3.0.550.

Pcpfm Workflows Description

For the comparison of MetaboAnalystR and pcpfm, a minimal pcpfm workflow was employed. The minimal workflow consists of experiment assembly, asari processing using the default asari parameters, and subsequent empirical compound construction using the full asari feature table. The MetaboAnalystR workflow mimics this minimal workflow and consists of feature and is

implemented using MetaboAnalystR v4.0.0, OptiLCMS v.1.1.0 which implements methods derived from XCMS, MSnBase, and CAMERA ran in Rstudio v. 2023.12.0 build 369 with an R v4.3.2 interpreter. Automatic parameter optimization and rt.idx of 0.9 for ROI detection and an mz_abs_add of 0.015 were used for annotation in the MetaboAnalystR workflow.

Other analyses were performed using an appropriately modified ‘default’ workflow. The default workflow includes basic QA/QC processing as described below and the default parameters each step. This workflow has proved suitable for experiments performed in-house but may require modification for use with other experimental designs. The default workflow consists of experiment assembly, asari pre-processing with automatic inference of ionization modality, blank masking using all samples with sample_type including the substring “Blank” and an intensity ratio cutoff of 3, meaning features whose mean intensity, excluding zeros, do not exceed three times the mean intensity in blanks, excluding zeros, are dropped. After blank masking, non-study samples are dropped, assumed to have a sample_type of “Unknown”, and then outliers are dropped using a Z-score cutoff of 2.5 on the number of features Z-score. The remaining study samples are normalized using a one-step process to the median TIC of all the samples calculated using features present in over 90% of samples. Features present in less than 50% of samples are then dropped and remaining missing values are imputed using .50 times the minimum value of that feature observed in the feature table before finally log2 transforming the feature table. These operations are performed only on the preferred feature table as they represent the high-quality features. The full feature table is then used to generate empirical compounds using common adducts for the respective ionization mode and ^{13}C isotopologues up to m+$^{13}C_3$ only. Experiments using isotope labelling will require an extended isotopologue range. These empirical compounds are then level4 annotated using both the HMDB and LMSD and then output is generated using the final preferred feature table and the annotated empirical compounds.

Modifications of this default or minimal workflow are used for the analyses presented in the manuscript both to demonstrate the feasibility of modifying the workflow but also to account for differences in the experimental designs of the experiments. For the Ansone et al. 2021 dataset, a default workflow with the asari autoheight option is enabled. For Bowen et al. 2023 analyses, a default workflow is employed but no features are dropped based on their inclusion percentile in samples to account for the relative rarity of xenobiotic features and is expanded to also include processing of the full feature table. For the Checkmate analysis, a modified minimal

workflow is employed where empirical compounds are not constructed, essentially ending the analysis after the run of asari. For HZV029 QC and HZV029 Two-Phase HILIC-, blank masking is performed twice using first process blanks and then solvent blanks followed by removal of non-study samples and the dropping of outliers. No annotation is performed. For HZV029 RP- and HILIC+ annotation results are generated using the default workflow but expanded to include MS^2 mapping and level 2 annotation. Due to combat's sensitivity to missing values, an alternative batch correction of the default workflow is used for analyses requiring batch correction that features an aggressive cutoff for the missing features (90% vs. the default 50%).

References

1. Siddiqa A, Wang Y, Thapa M, Martin DE, Cadar AN, Bartley JM, et al. A pilot metabolomic study of drug interaction with the immune response to seasonal influenza vaccination. npj Vaccines. 2023;8(1):92. doi: 10.1038/s41541-023-00682-2.
2. Bowen TJ, Southam AD, Hall AR, Weber RJM, Lloyd GR, Macdonald R, et al. Simultaneously discovering the fate and biochemical effects of pharmaceuticals through untargeted metabolomics. Nat Commun. 2023;14(1):4653. Epub 2023/08/04. doi: 10.1038/s41467-023-40333-7. PubMed PMID: 37537184; PubMed Central PMCID: PMCPMC10400635.
3. Ansone L, Briviba M, Silamikelis I, Terentjeva A, Perkons I, Birzniece L, et al. Amino Acid Metabolism is Significantly Altered at the Time of Admission in Hospital for Severe COVID-19 Patients: Findings from Longitudinal Targeted Metabolomics Analysis. Microbiol Spectr. 2021;9(3):e0033821. Epub 2021/12/09. doi: 10.1128/spectrum.00338-21. PubMed PMID: 34878333; PubMed Central PMCID: PMCPMC8653833.
4. Matyash V, Liebisch G, Kurzchalia TV, Shevchenko A, Schwudke D. Lipid extraction by methyl-tert-butyl ether for high-throughput lipidomics. J Lipid Res. 2008;49(5):1137-46. Epub 2008/02/19. doi: 10.1194/jlr.D700041-JLR200. PubMed PMID: 18281723; PubMed Central PMCID: PMCPMC2311442.
5. Virtanen P, Gommers R, Oliphant TE, Haberland M, Reddy T, Cournapeau D, et al. SciPy 1.0: fundamental algorithms for scientific computing in Python. Nature methods. 2020;17(3):261-72.
6. Wada K. gdown. v4.7.1 ed: Github; 2024. p. Google Drive Public File Downloader when Curl/Wget Fails
7. Hunter JD. Matplotlib: A 2D Graphics Environment. Computing in Science & Engineering. 2007;9(3):90-5. doi: 10.1109/MCSE.2007.55.
8. Behdenna A, Colange M, Haziza J, Gema A, Appé G, Azencott C-A, et al. pyComBat, a Python tool for batch effects correction in high-throughput molecular data using empirical Bayes methods. BMC Bioinformatics. 2023;24(1):459. doi: 10.1186/s12859-023-05578-5.
9. Kramer O. Scikit-Learn. In: Kramer O, editor. Machine Learning for Evolution Strategies. Cham: Springer International Publishing; 2016. p. 45-53.

10. Kramer O, Kramer O. Scikit-learn. Machine learning for evolution strategies. 2016:45-53.
11. Florian Huber SV, Christiaan Meijer, Hanno Spreeuw, Efrain Manuel Villanueva Castilla, Culiang Geng, Justin J. J. van der Hooft, Simon Rogers, Adam Belloum, Faruk Diblen, and Jurriaan H. Spaaks. matchms - processing and similarity evaluation of mass spectrometry data. The Journal of Open Source Software. 2020;5(52):2411. doi: https://doi.org/10.21105/joss.02411.
12. Kösters M, Leufken J, Schulze S, Sugimoto K, Klein J, Zahedi RP, et al. pymzML v2.0: introducing a highly compressed and seekable gzip format. Bioinformatics. 2018;34(14):2513-4. doi: 10.1093/bioinformatics/bty046.
13. Waskom M, Botvinnik O, O'Kane D, Hobson P, Lukauskas S, Gemperline DC, et al. mwaskom/seaborn: v0.8.1 (September 2017). v0.8.1 ed: Zenodo; 2017.
14. Slabon J. FPDF. v1.86 ed: github; 2023. p. FPDF is a PHP class which allows to generate PDF files with pure PHP. F from FPDF stands for Free: you may use it for any kind of usage and modify it to suit your needs.
15. Li S, Siddiqa A, Thapa M, Chi Y, Zheng S. Trackable and scalable LC-MS metabolomics data processing using asari. Nature Communications. 2023;14(1):4113. doi: 10.1038/s41467-023-39889-1.
16. Oliphant TE. Guide to numpy: Trelgol Publishing USA; 2006.
17. McKinney W. pandas: a foundational Python library for data analysis and statistics. Python for high performance and scientific computing. 2011;14(9):1-9.
