# Supplementary material for: Common data models to streamline metabolomics processing and annotation, and implementation in a Python pipeline
Source: PLoS Comput Biol. 2024 Jun 6;20(6):e1011912. doi: 10.1371/journal.pcbi.1011912 (PMC11185459; doi:10.1371/journal.pcbi.1011912)
Supplement: S2 File — (PDF) [file pcbi.1011912.s004.pdf]

PCPFM Report - HZV029_plasma_HILIC_pos

Timestamp

Report generated on 2024-04-12 17:16:59.335703

Feature Table Summary

A feature denotes a region of a spectrum believed to represent a ion of a compound with a retention time and a mass-to-charge ratio. Multiple features often represent the same metabolite due to isotopologues, adduct, multiple charges etc. and thus, the number of features is only a rough proxy for the number of detected metabolites. Due to noise, artifacts, rare metabolites, etc. the number of features often increases with the number of samples.

Table Name, Num Samples, Num Features
full, 1684, 40007
preferred, 1684, 28051
preferred_blank_masked, 1684, 23832
masked_preferred_unknowns, 1329, 21895
qaqc_filtered_masked_pref_unknowns, 1318, 21730
pref_normalized, 1318, 21730
pref_dropped, 1318, 2291
pref_interpolated, 1318, 2291
for_analysis, 1318, 2291

empCpd Table Summary

Empirical compounds are computational intermediates representing sets of features suspected to correspond to the same compound. Each empirical compound is a khipu, thus, the number of khipus is an estimateof the number of detected metabolites. Each khipu represents multiple features; however, unless singletons were added to the khipu during construction, the number of features grouped captured by empCpds is less thanthe number of features.

EmpCpd Name, Num Khipus, Num Features
asari, 28207, 40008
for_analysis, 27866, 40008
for_analysis2, 27866, 40008
HMDB_LMSD_annotated_for_analysis, 27866, 40008
MoNA_HMDB_LMSD_annotated_for_analysis, 27866, 40008

Experiment Summary

empCpd list
asari
for_analysis
for_analysis2
HMDB_LMSD_annotated_for_analysis
MoNA_HMDB_LMSD_annotated_for_analysis

Feature Table list
full
preferred
preferred_blank_masked
masked_preferred_unknowns
qaqc_filtered_masked_pref_unknowns
pref_normalized
pref_dropped

PCPFM Report - HZV029_plasma_HILIC_pos

pref_interpolated
for_analysis

Annotation Summary

Annotations are mappings of features / empCpds to suspected chemical entities. Annotations can be higher or lower confidence depending on the origin in which they are generated. In general, MS1 annotated features are lower confidence than MS2 annotated features.

Name, #EmpCpds, #l4 Annotated, #l2 Annotated, #l1b Annotated, #l1a Annotated
asari, 28207, 0, 0, 0, 0
for_analysis, 27866, 0, 0, 0, 0
for_analysis2, 27866, 0, 0, 0, 0
HMDB_LMSD_annotated_for_analysis, 27866, 16434, 0, 0, 0
MoNA_HMDB_LMSD_annotated_for_analysis, 27866, 16434, 0, 0, 0

PCPFM Report - HZV029_plasma_HILIC_pos

Table: full Figure: pca

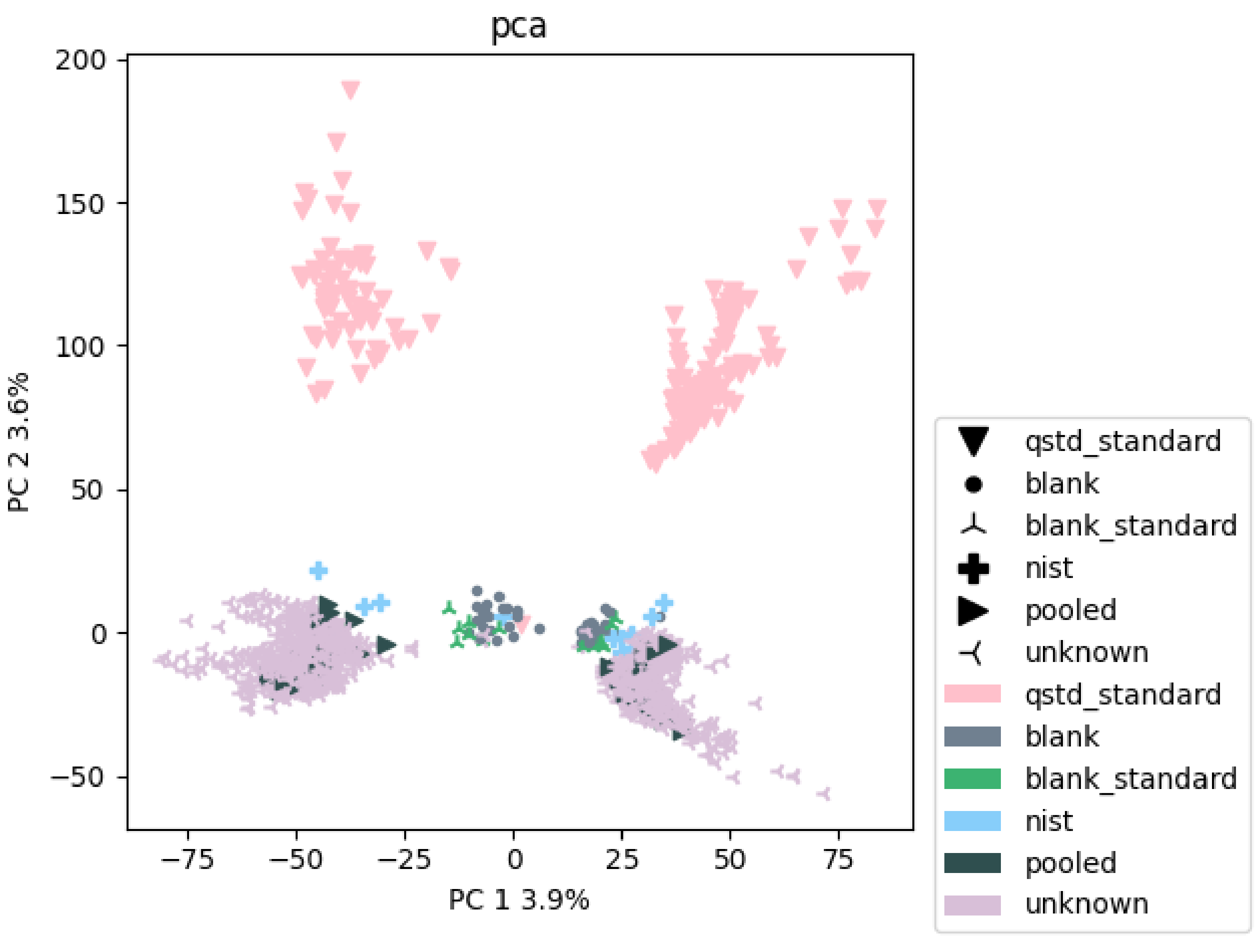

This is PCA performed on the specified feature table. It has standardized to a mean of zero and unit variance.

PCPFM Report - HZV029_plasma_HILIC_pos

Table: preferred Figure: pca

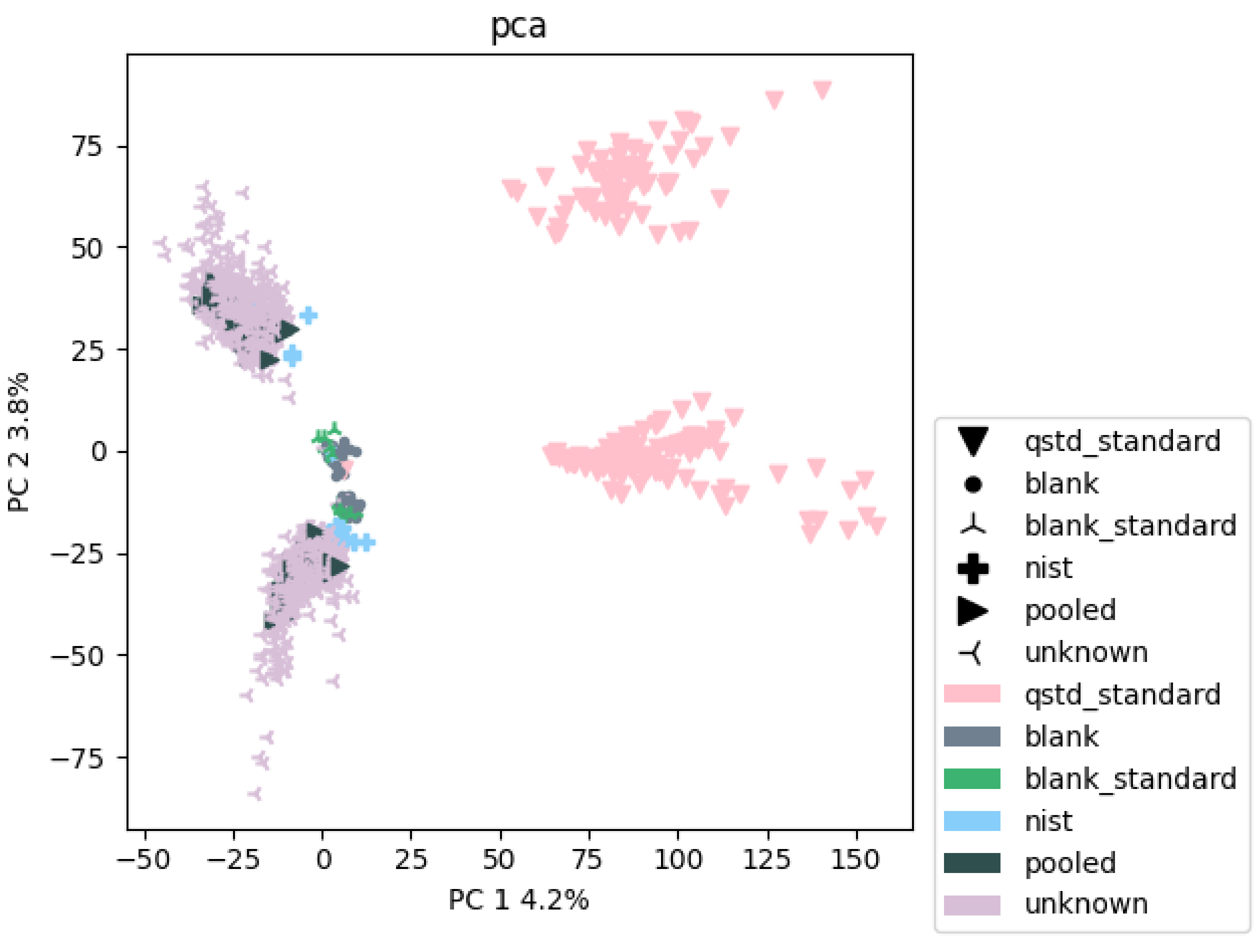

This is PCA performed on the specified feature table. It has standardized to a mean of zero and unit variance.

PCPFM Report - HZV029_plasma_HILIC_pos

Table: preferred_blank_masked Figure: pca

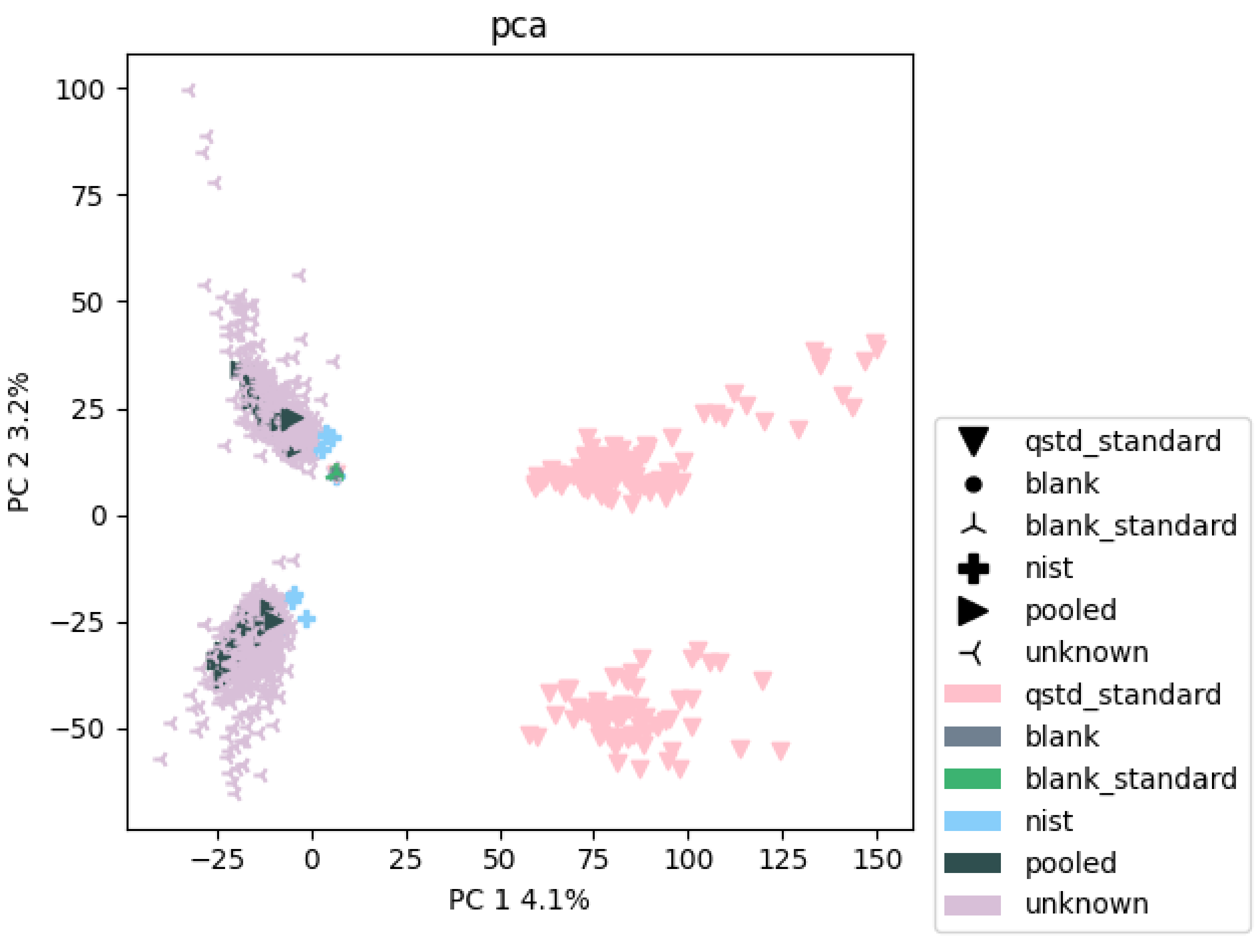

This is PCA performed on the specified feature table. It has standardized to a mean of zero and unit variance.

PCPFM Report - HZV029_plasma_HILIC_pos

Table: masked_preferred_unknowns Figure: pca

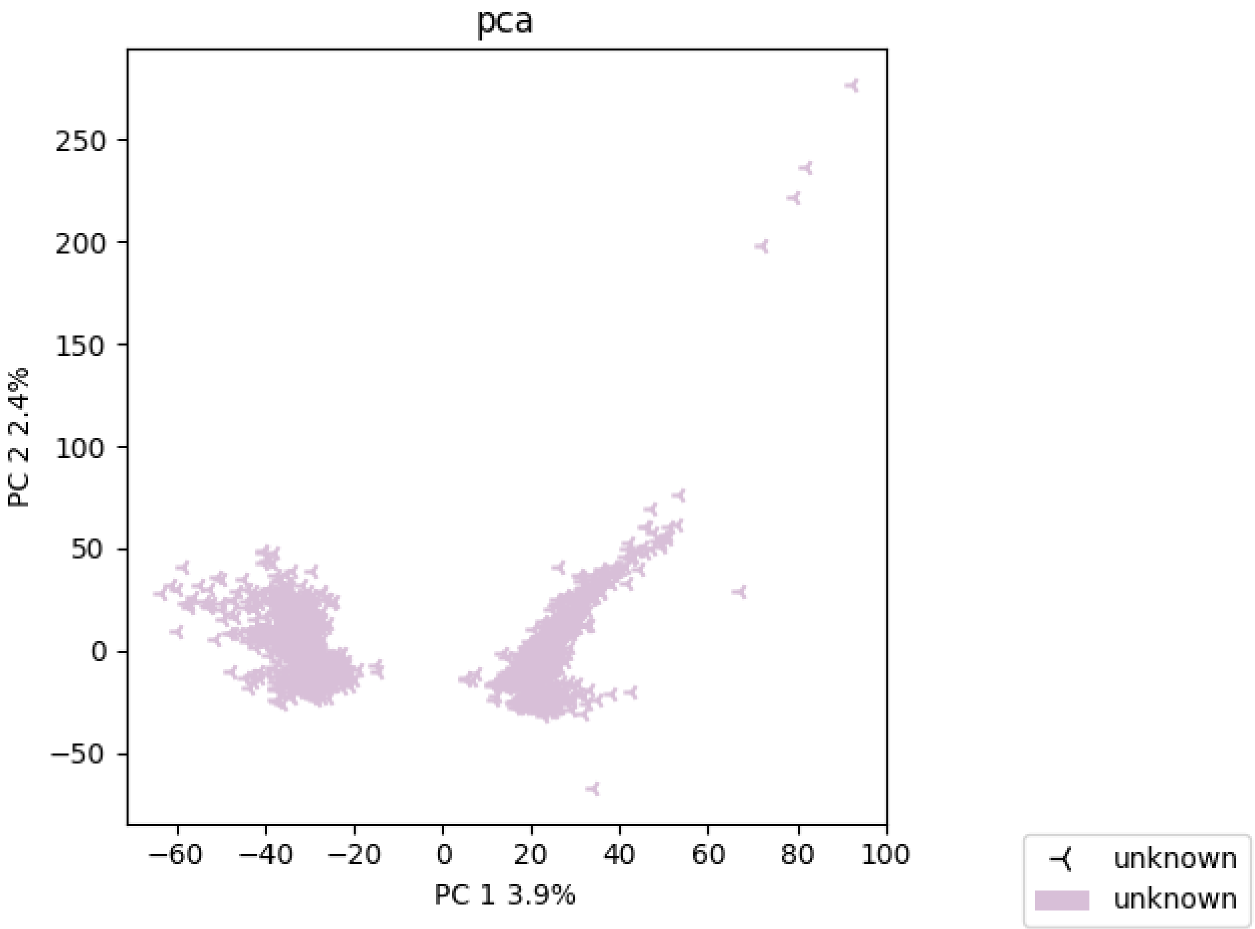

This is PCA performed on the specified feature table. It has standardized to a mean of zero and unit variance.

PCPFM Report - HZV029_plasma_HILIC_pos

Table: qaqc_filtered_masked_pref_unknowns Figure: pca

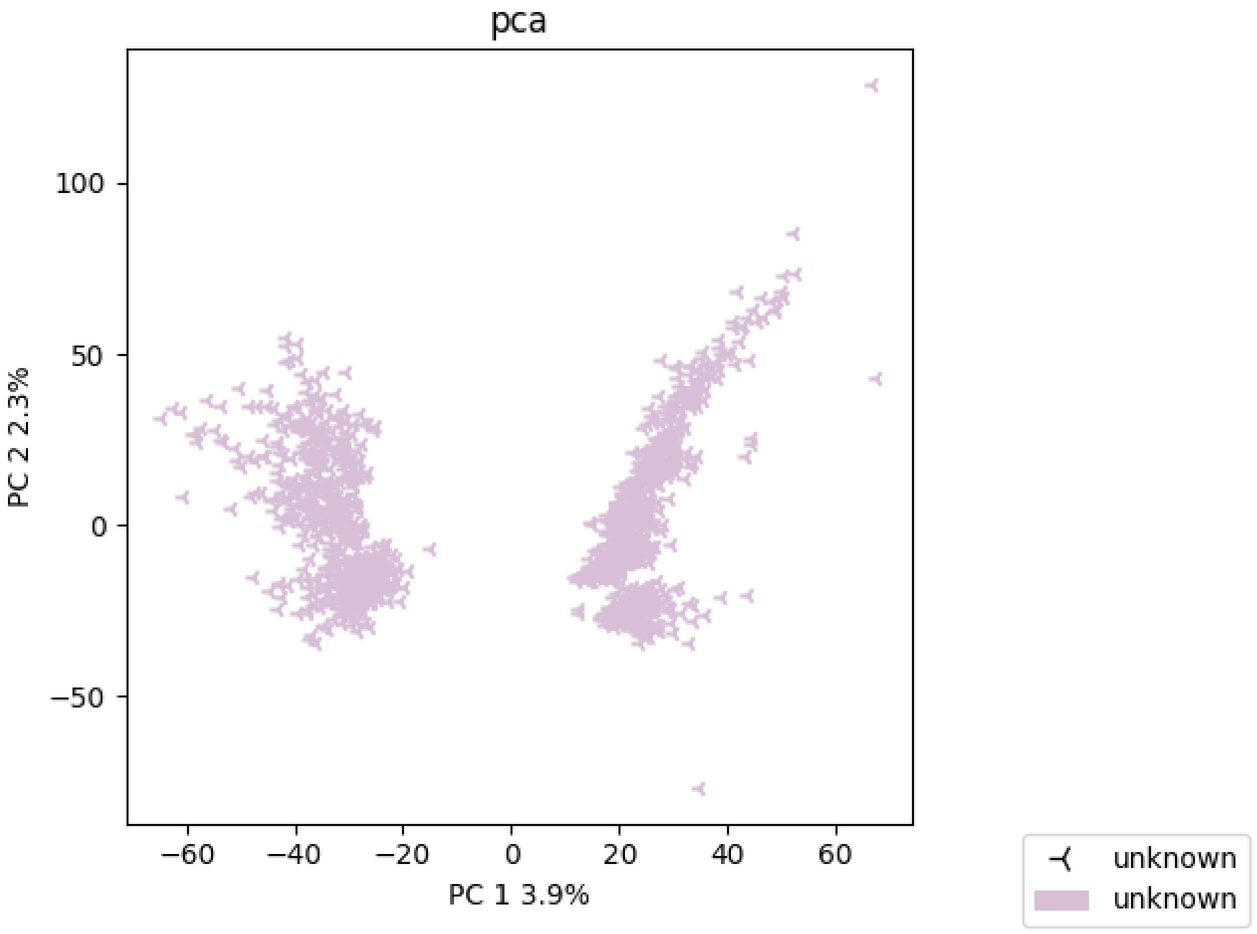

This is PCA performed on the specified feature table. It has standardized to a mean of zero and unit variance.

PCPFM Report - HZV029_plasma_HILIC_pos

Table: pref_normalized Figure: pca

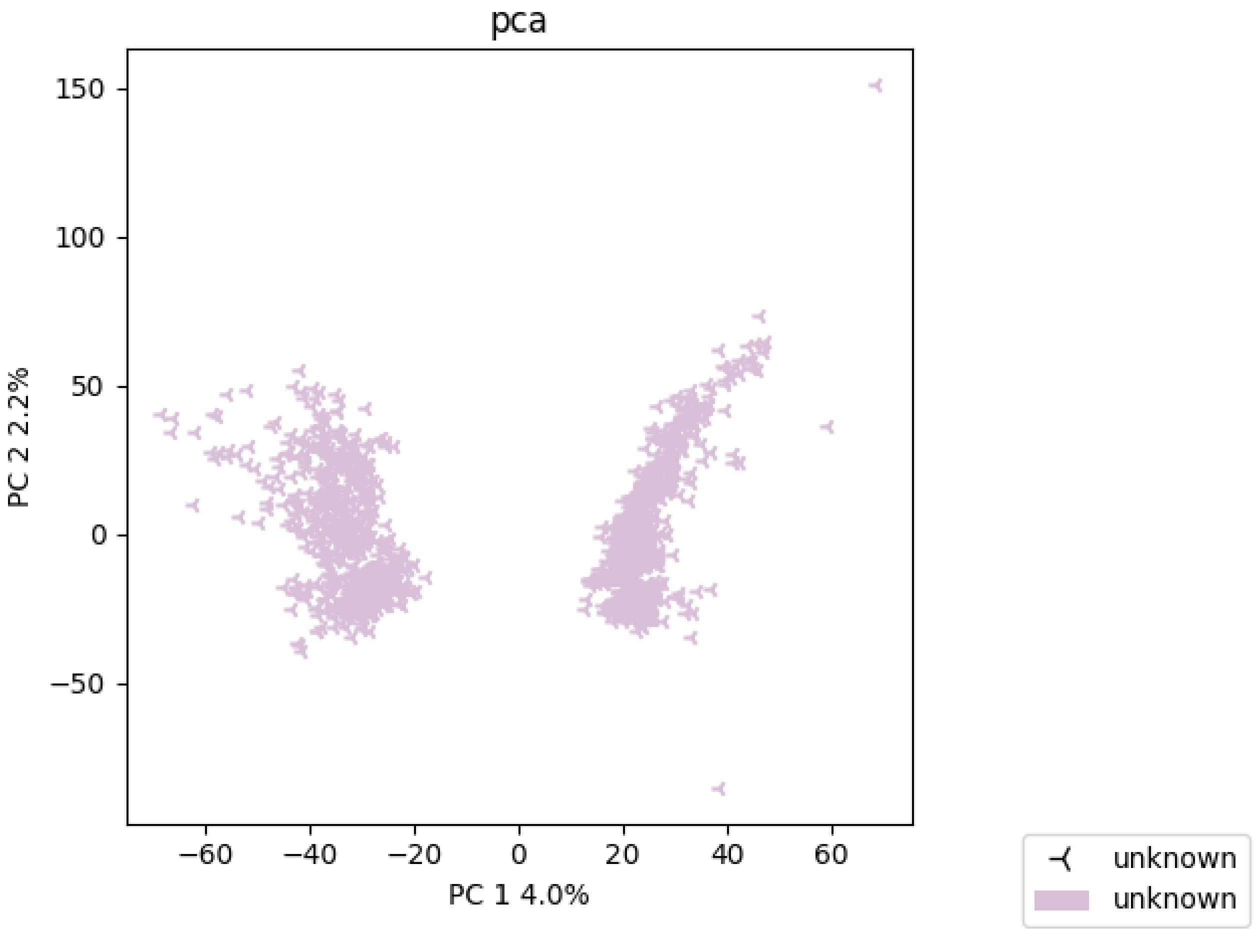

This is PCA performed on the specified feature table. It has standardized to a mean of zero and unit variance.

PCPFM Report - HZV029_plasma_HILIC_pos

Table: pref_dropped Figure: pca

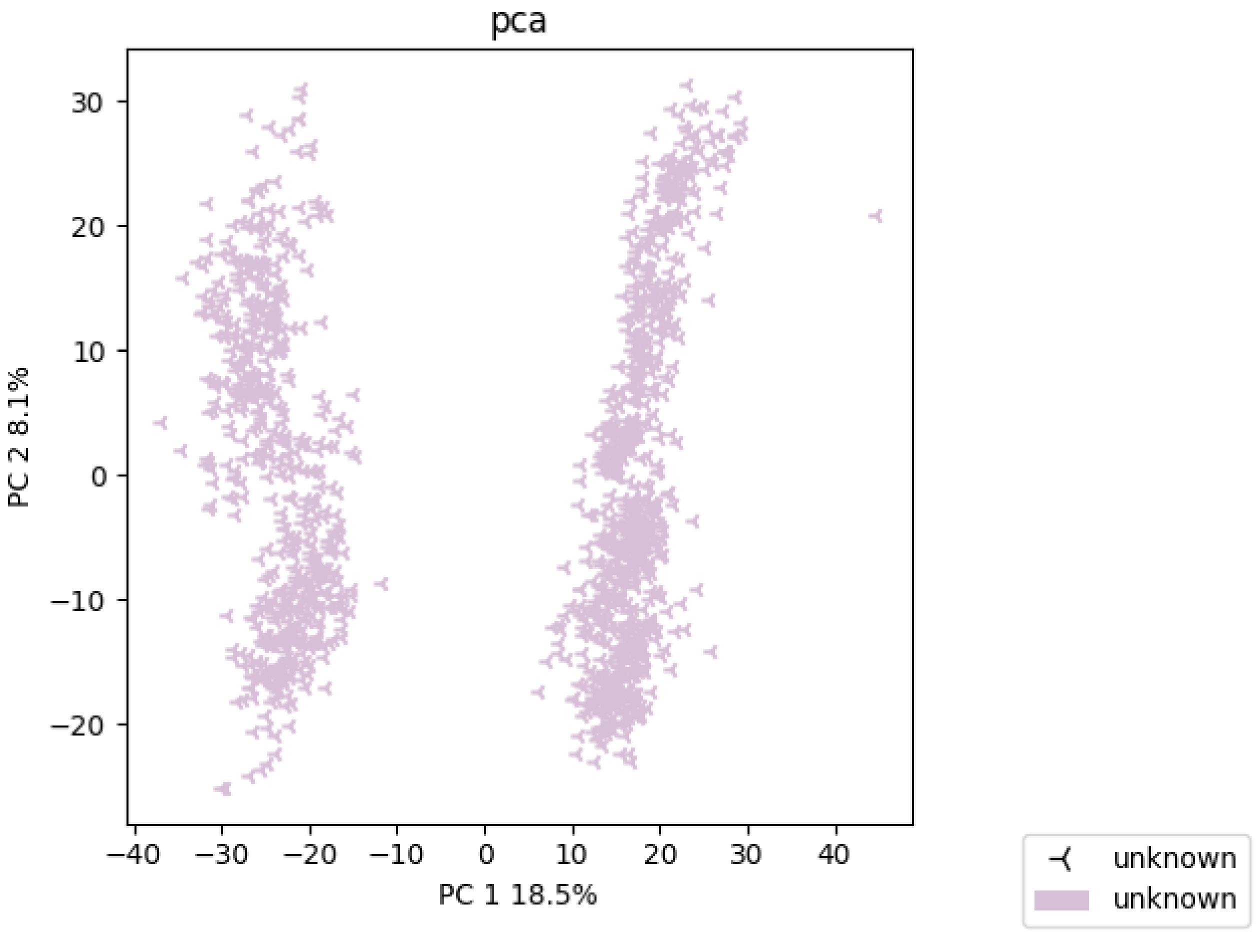

This is PCA performed on the specified feature table. It has standardized to a mean of zero and unit variance.

PCPFM Report - HZV029_plasma_HILIC_pos

Table: pref_interpolated Figure: pca

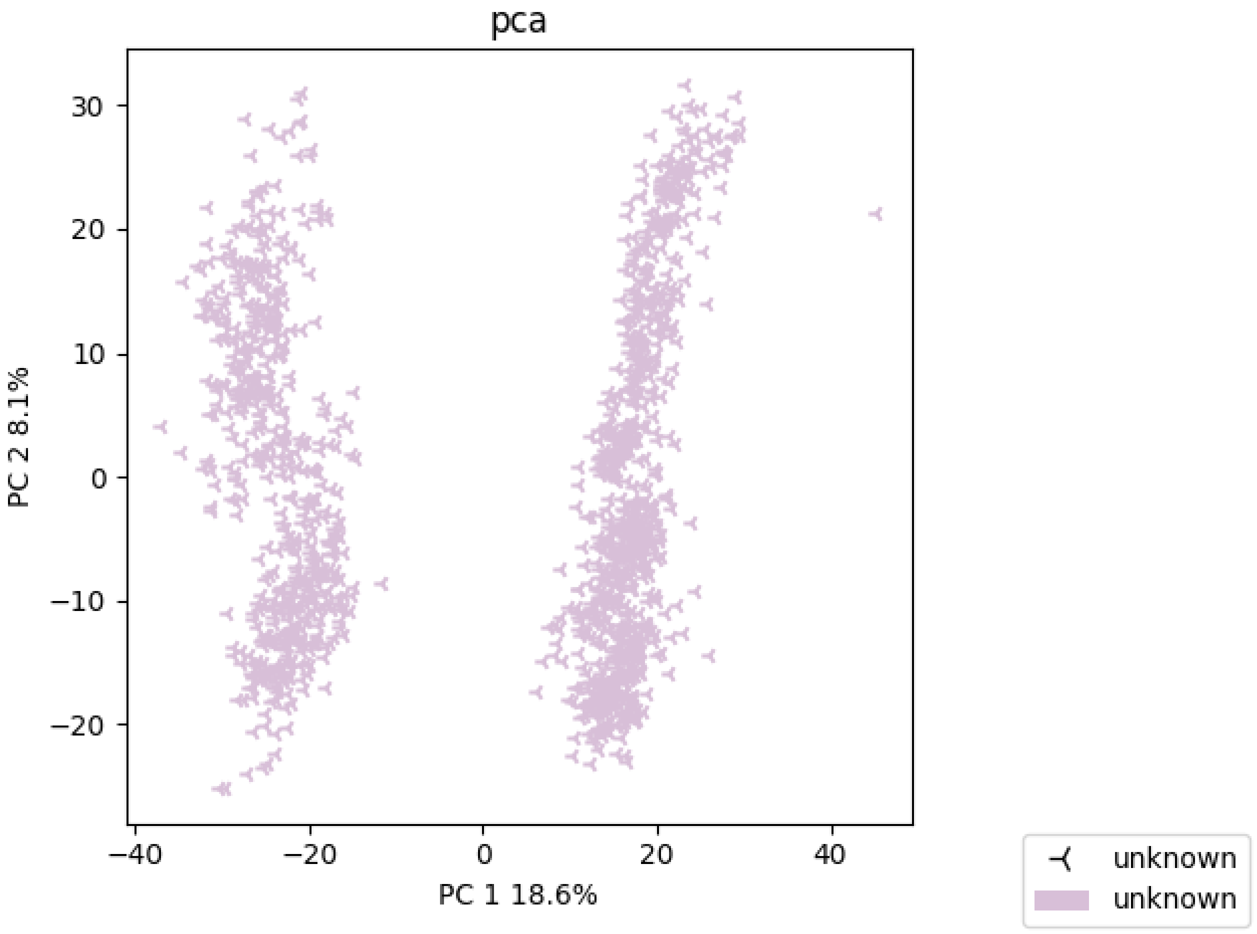

This is PCA performed on the specified feature table. It has standardized to a mean of zero and unit variance.

PCPFM Report - HZV029_plasma_HILIC_pos

Table: for_analysis Figure: pca

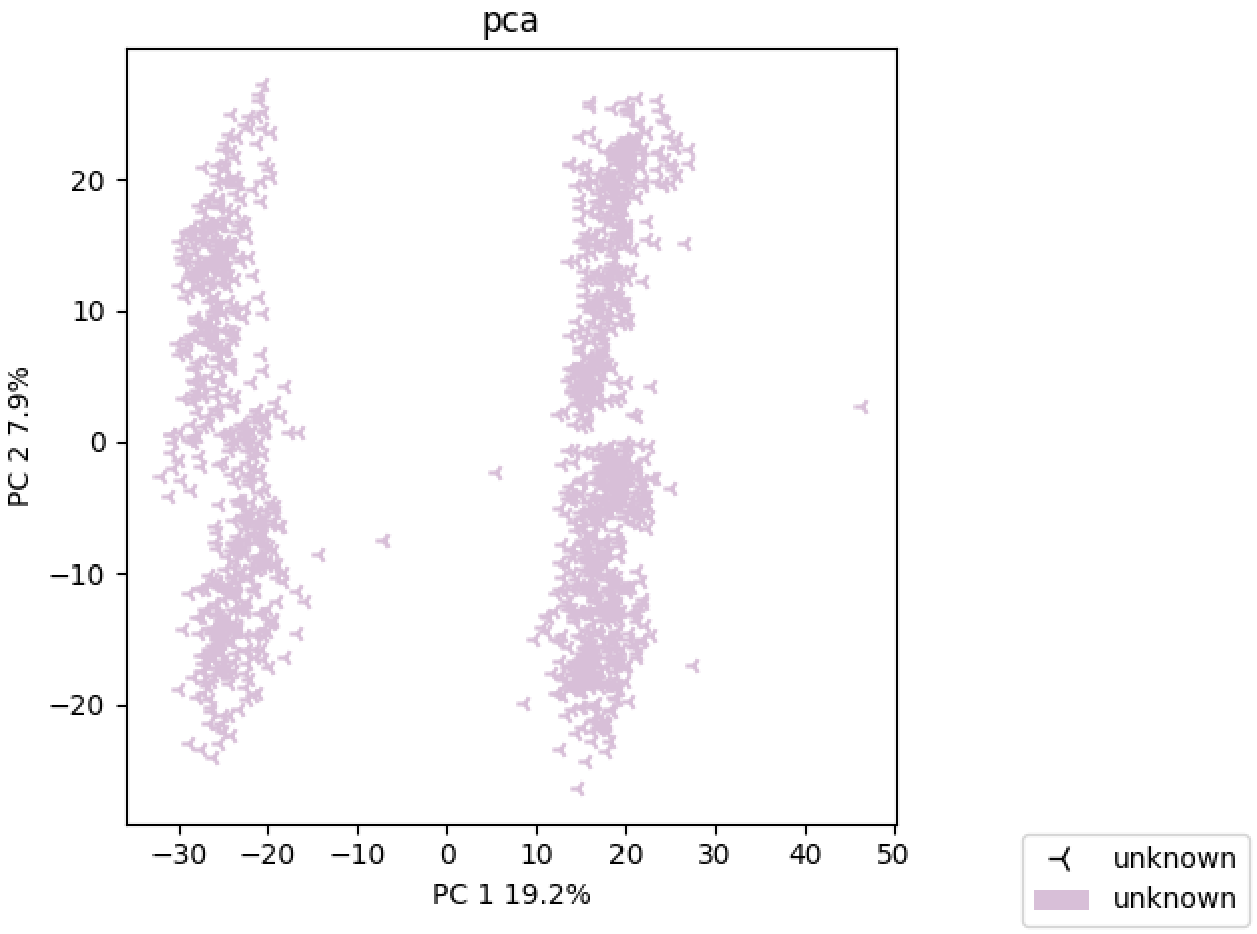

This is PCA performed on the specified feature table. It has standardized to a mean of zero and unit variance.

PCPFM Report - HZV029_plasma_HILIC_pos

Table: full Figure: log_pca

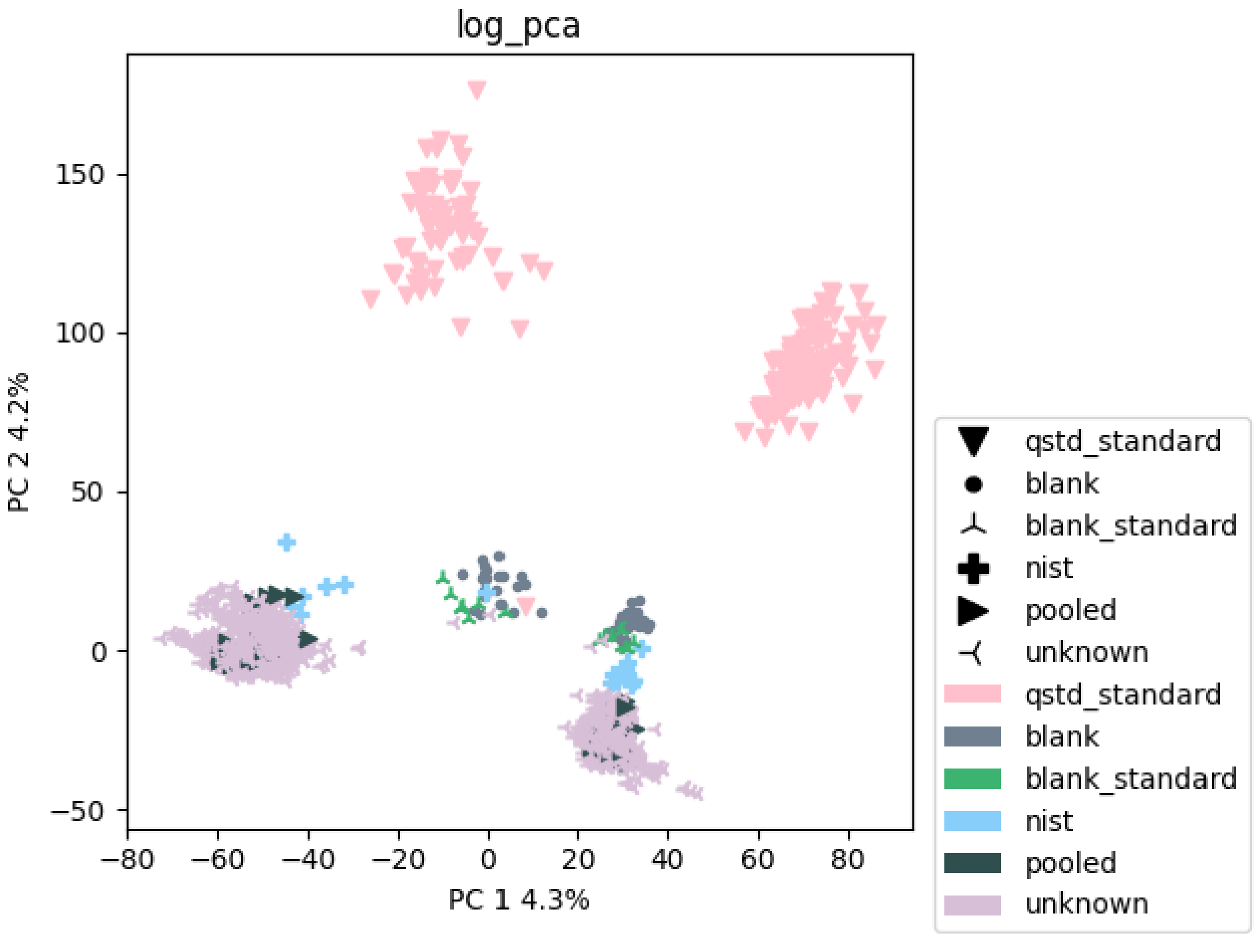

This is PCA performed on the log2 transformed feature table. This is very similar to PCA without log transform but may better handle outliers. It has standardized to a mean of zero and unit variance.

PCPFM Report - HZV029_plasma_HILIC_pos

Table: preferred Figure: log_pca

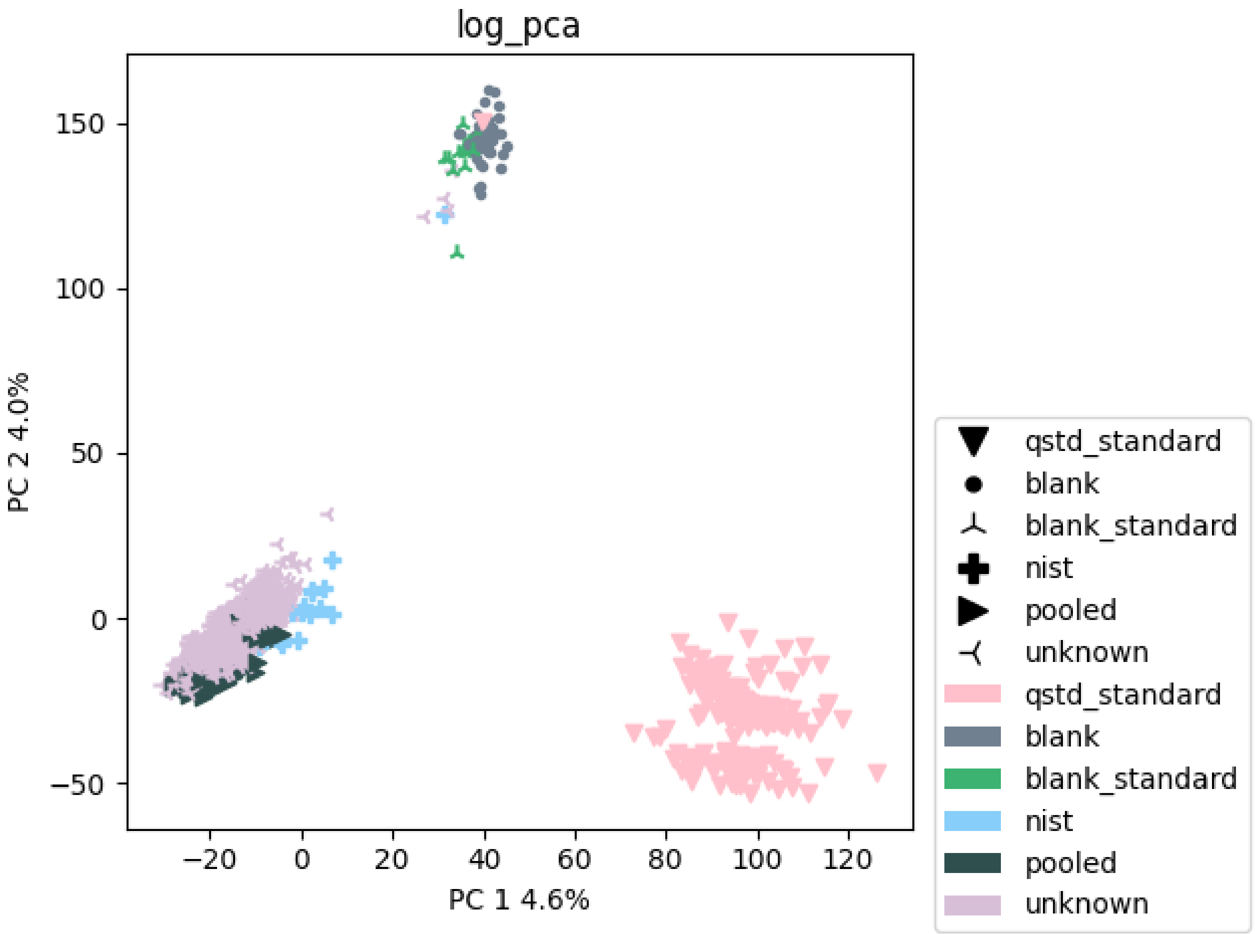

This is PCA performed on the log2 transformed feature table. This is very similar to PCA without log transform but may better handle outliers. It has standardized to a mean of zero and unit variance.

PCPFM Report - HZV029_plasma_HILIC_pos

Table: preferred_blank_masked Figure: log_pca

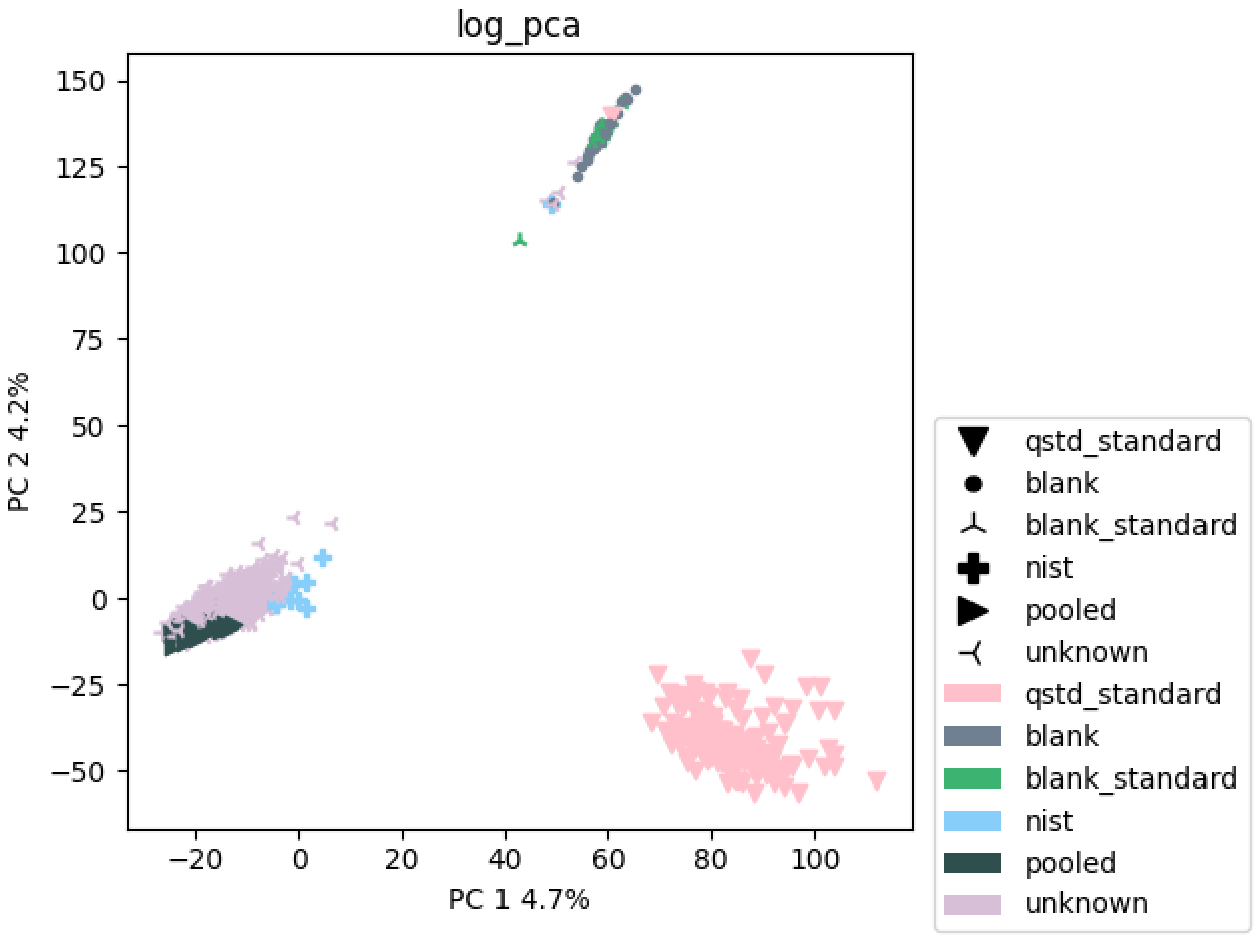

This is PCA performed on the log2 transformed feature table. This is very similar to PCA without log transform but may better handle outliers. It has standardized to a mean of zero and unit variance.

PCPFM Report - HZV029_plasma_HILIC_pos

Table: masked_preferred_unknowns Figure: log_pca

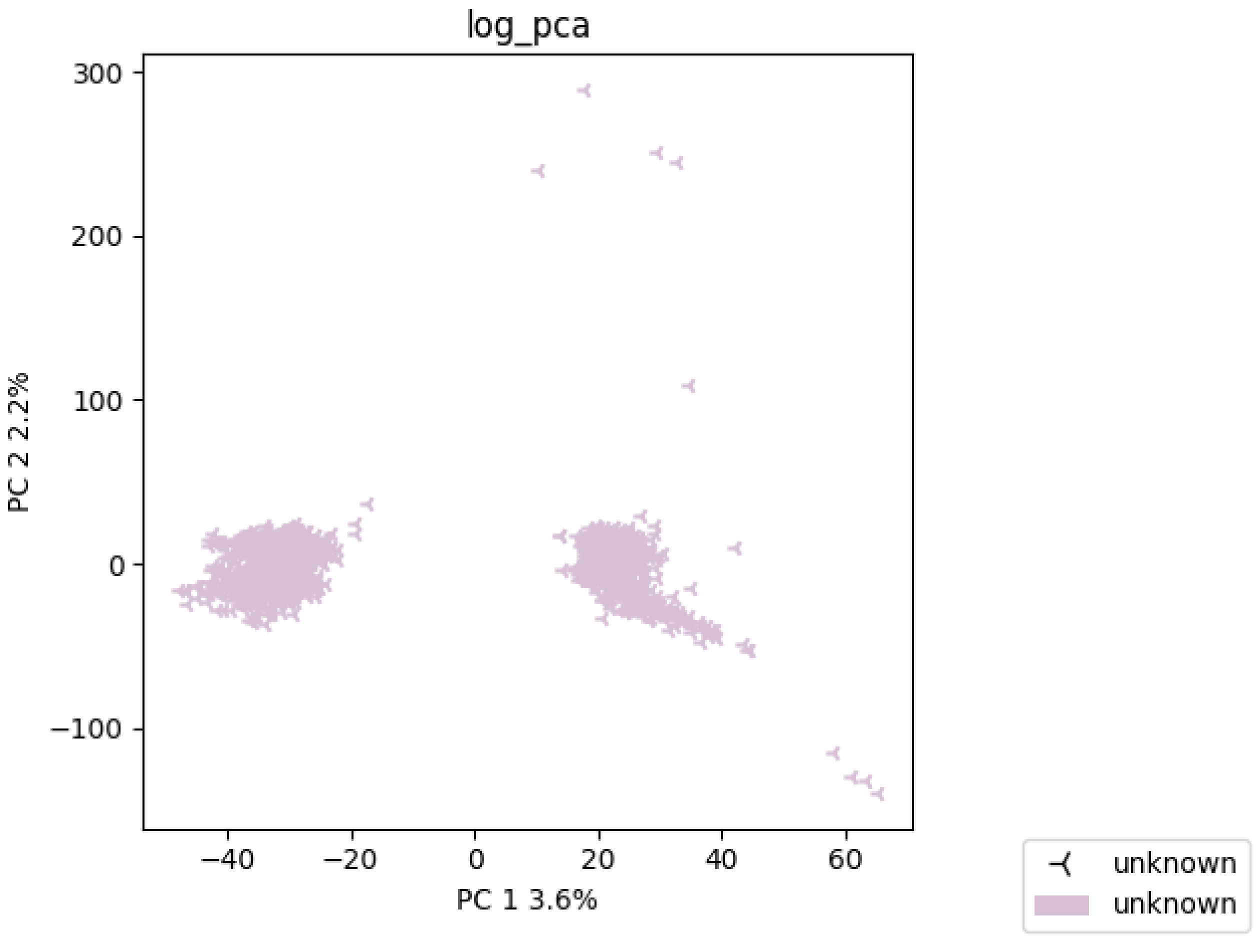

This is PCA performed on the log2 transformed feature table. This is very similar to PCA without log transform but may better handle outliers. It has standardized to a mean of zero and unit variance.

PCPFM Report - HZV029_plasma_HILIC_pos

Table: qaqc_filtered_masked_pref_unknowns Figure: log_pca

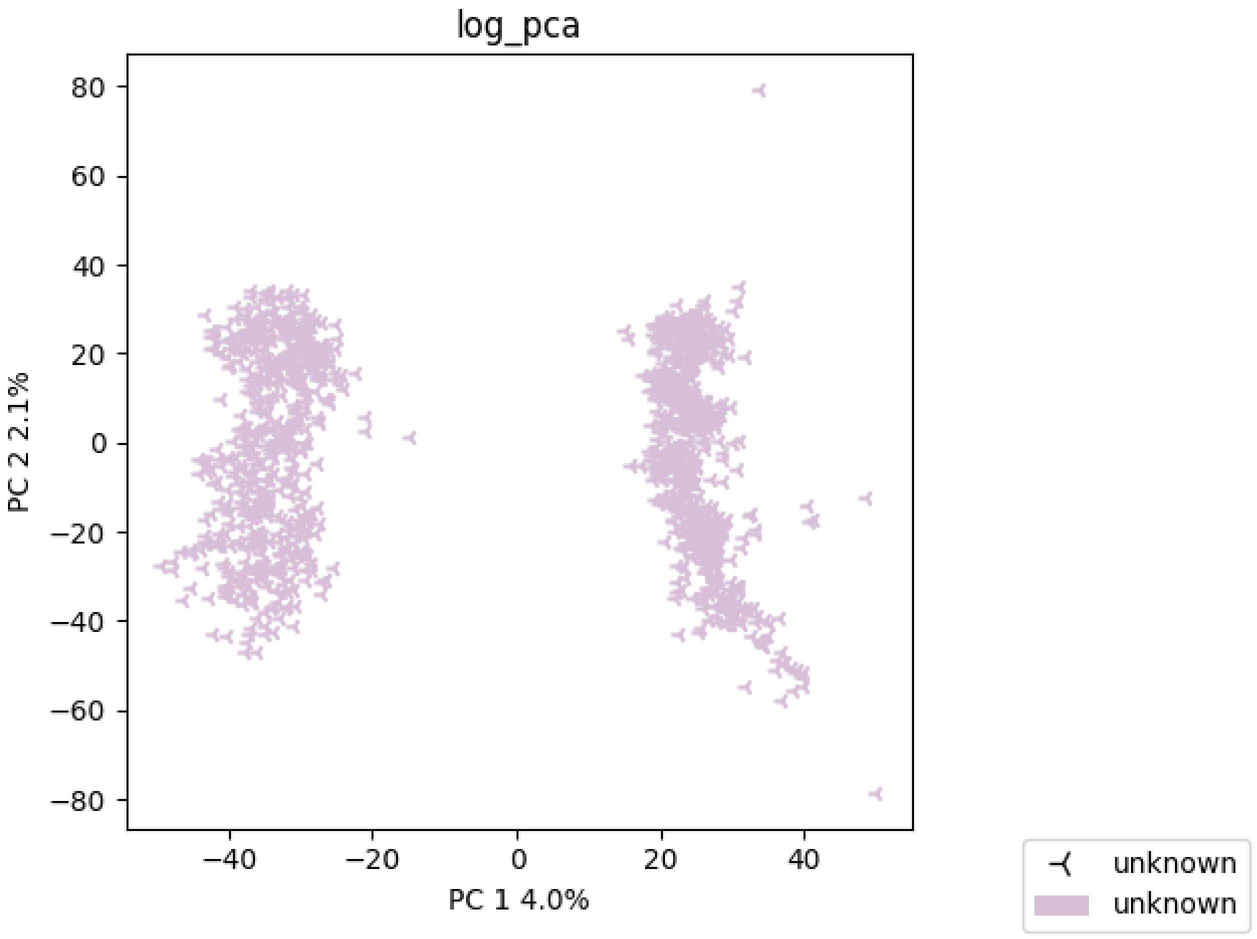

This is PCA performed on the log2 transformed feature table. This is very similar to PCA without log transform but may better handle outliers. It has standardized to a mean of zero and unit variance.

PCPFM Report - HZV029_plasma_HILIC_pos

Table: pref_normalized Figure: log_pca

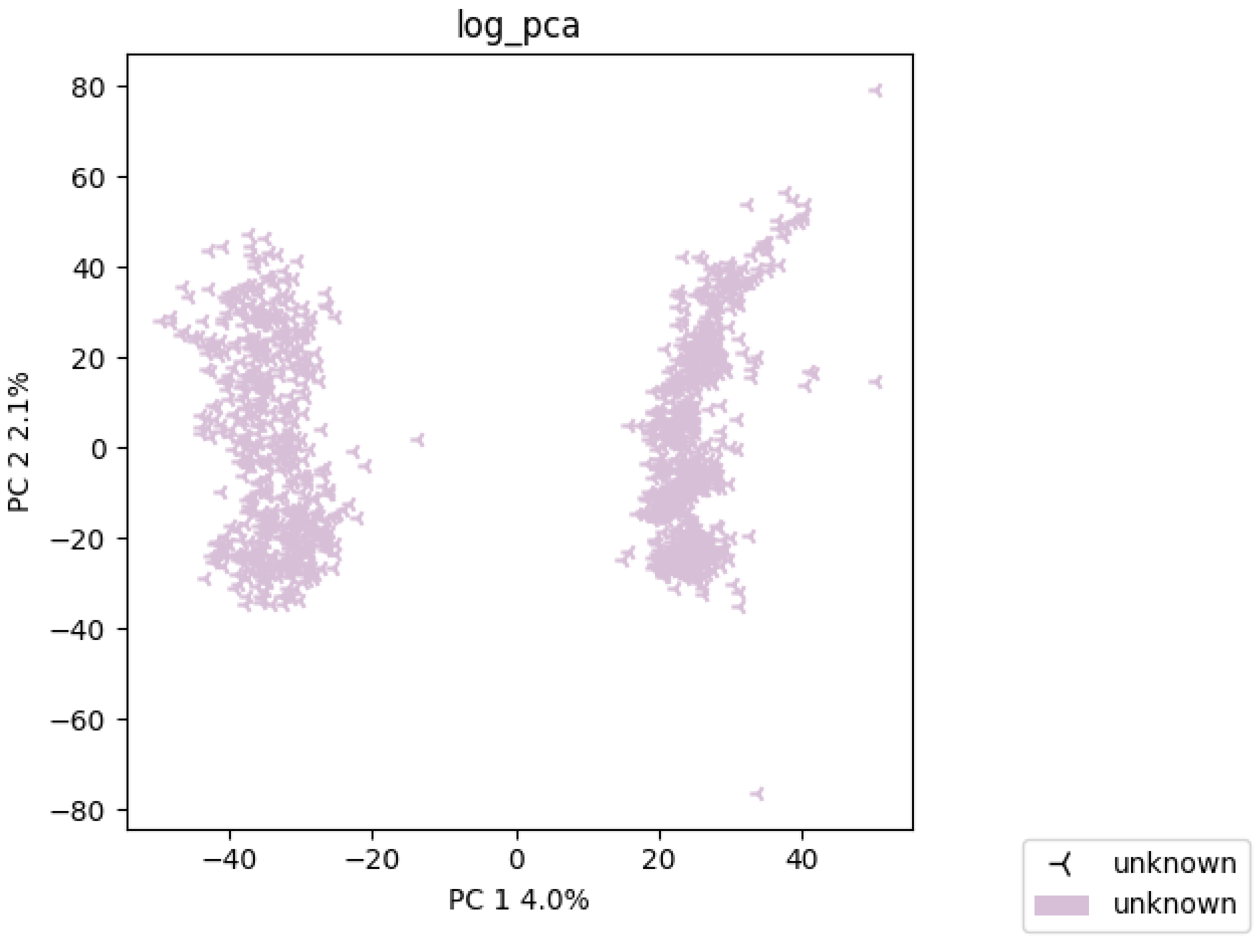

This is PCA performed on the log2 transformed feature table. This is very similar to PCA without log transform but may better handle outliers. It has standardized to a mean of zero and unit variance.

PCPFM Report - HZV029_plasma_HILIC_pos

Table: pref_dropped Figure: log_pca

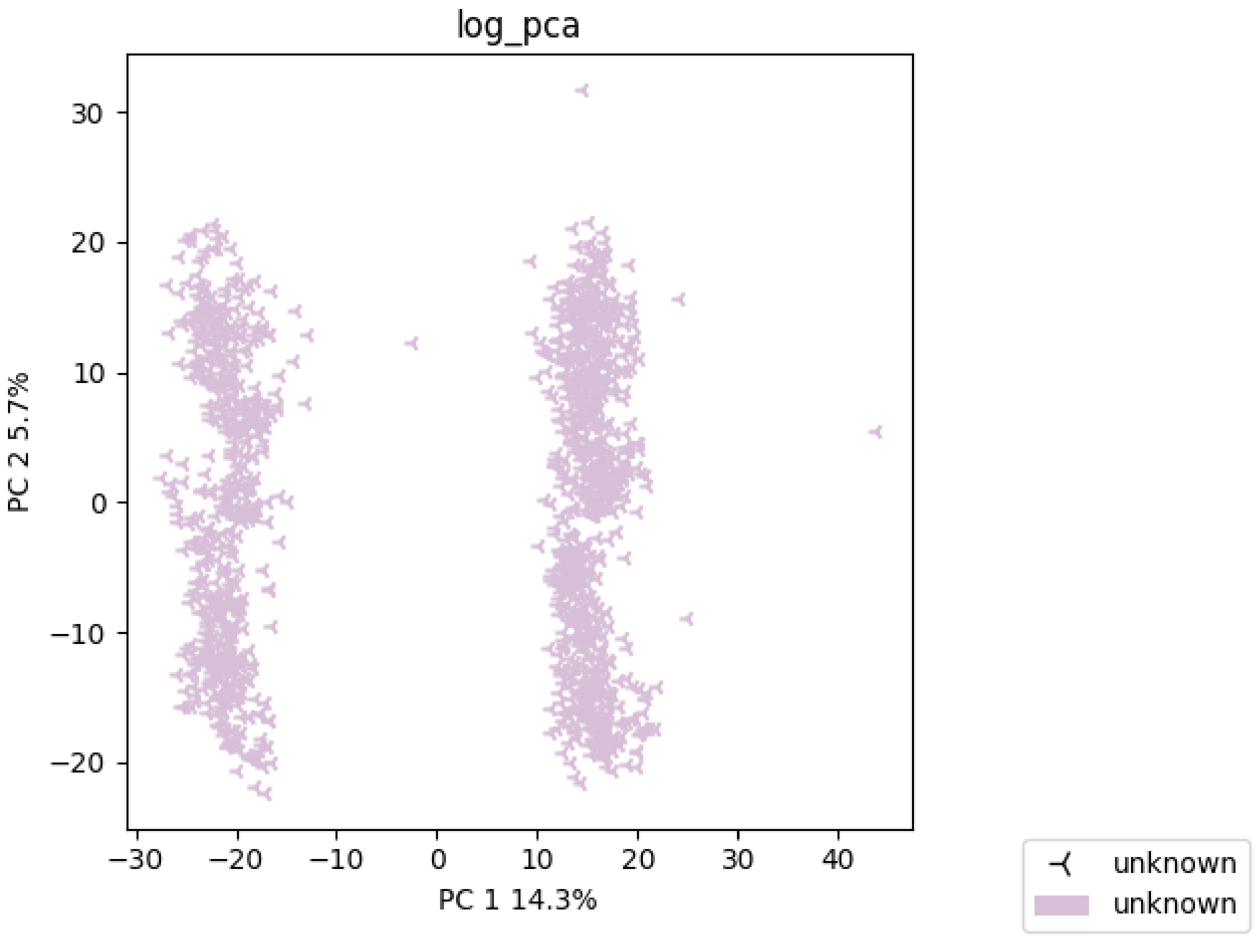

This is PCA performed on the log2 transformed feature table. This is very similar to PCA without log transform but may better handle outliers. It has standardized to a mean of zero and unit variance.

PCPFM Report - HZV029_plasma_HILIC_pos

Table: pref_interpolated Figure: log_pca

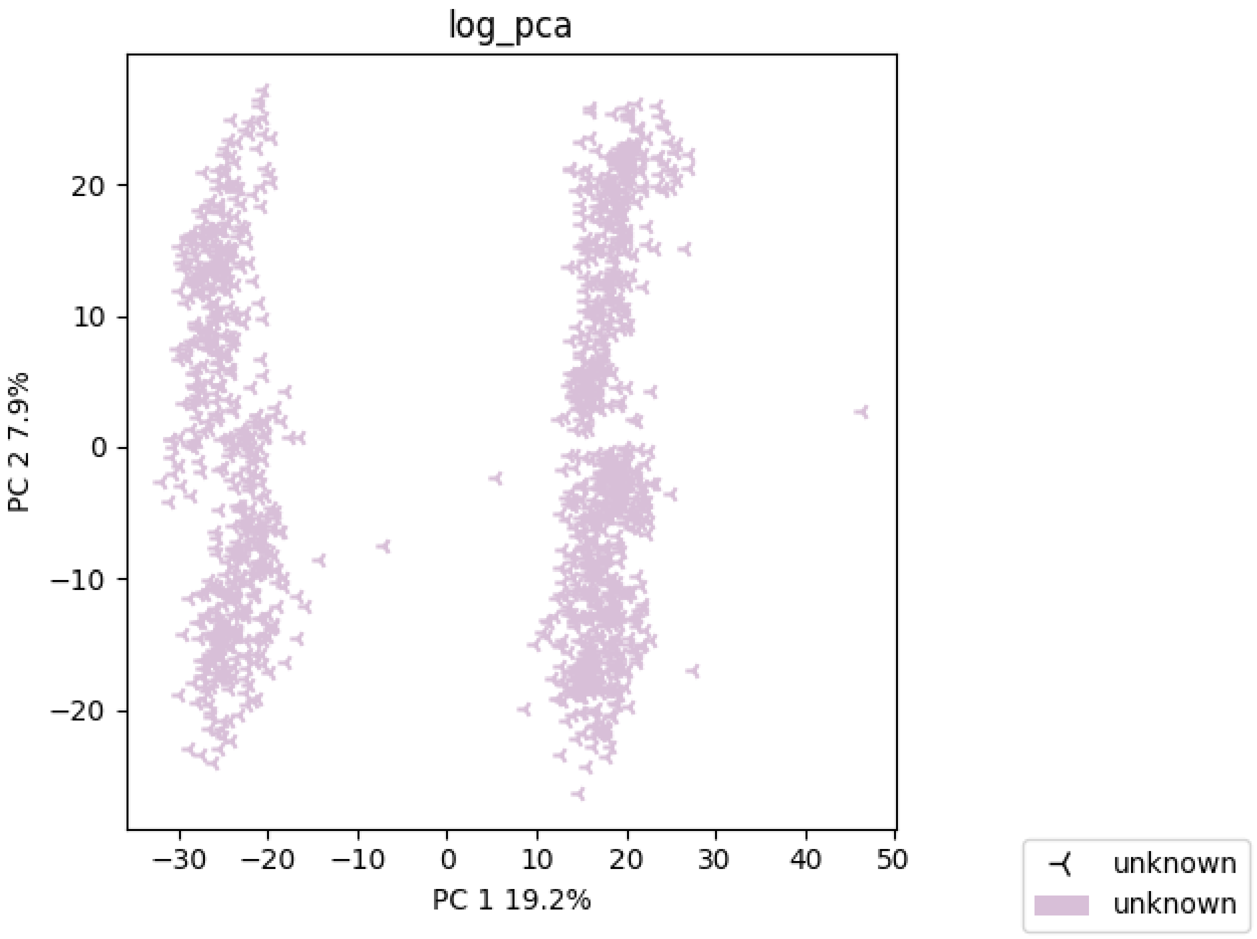

This is PCA performed on the log2 transformed feature table. This is very similar to PCA without log transform but may better handle outliers. It has standardized to a mean of zero and unit variance.

PCPFM Report - HZV029_plasma_HILIC_pos

Table: for_analysis Figure: log_pca

PCPFM Report - HZV029_plasma_HILIC_pos

Table: full Figure: pearson_correlation

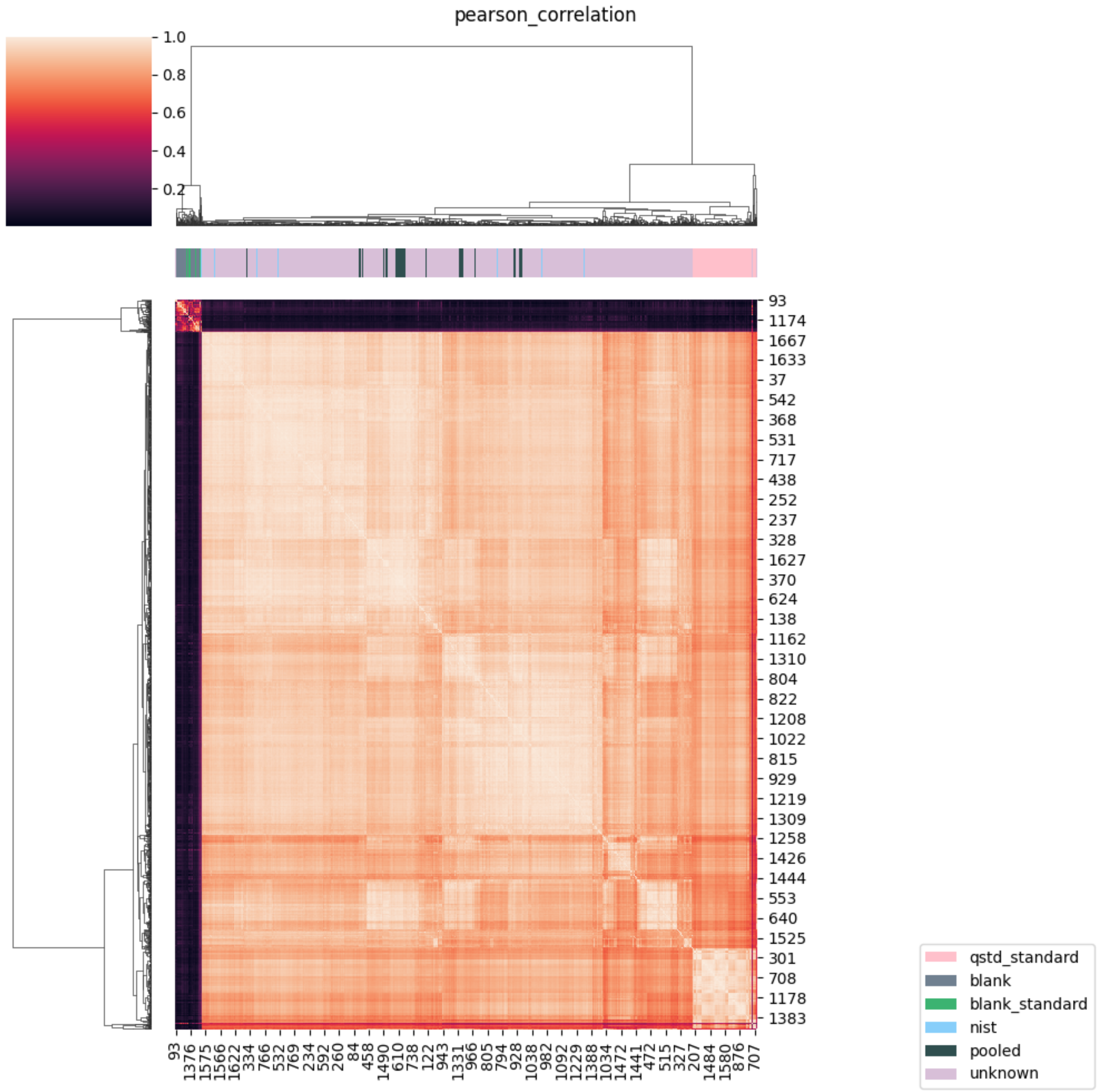

The pearson correlation represents how similar samples in feature intensities but assumes linearity. Higher correlation typically implies more similarity in samples; however, if many features are missing and have zero values, this correlation will appear falsely high. More informative correlation will be capture after imputing missing values and log transforming.

PCPFM Report - HZV029_plasma_HILIC_pos

Table: preferred Figure: pearson_correlation

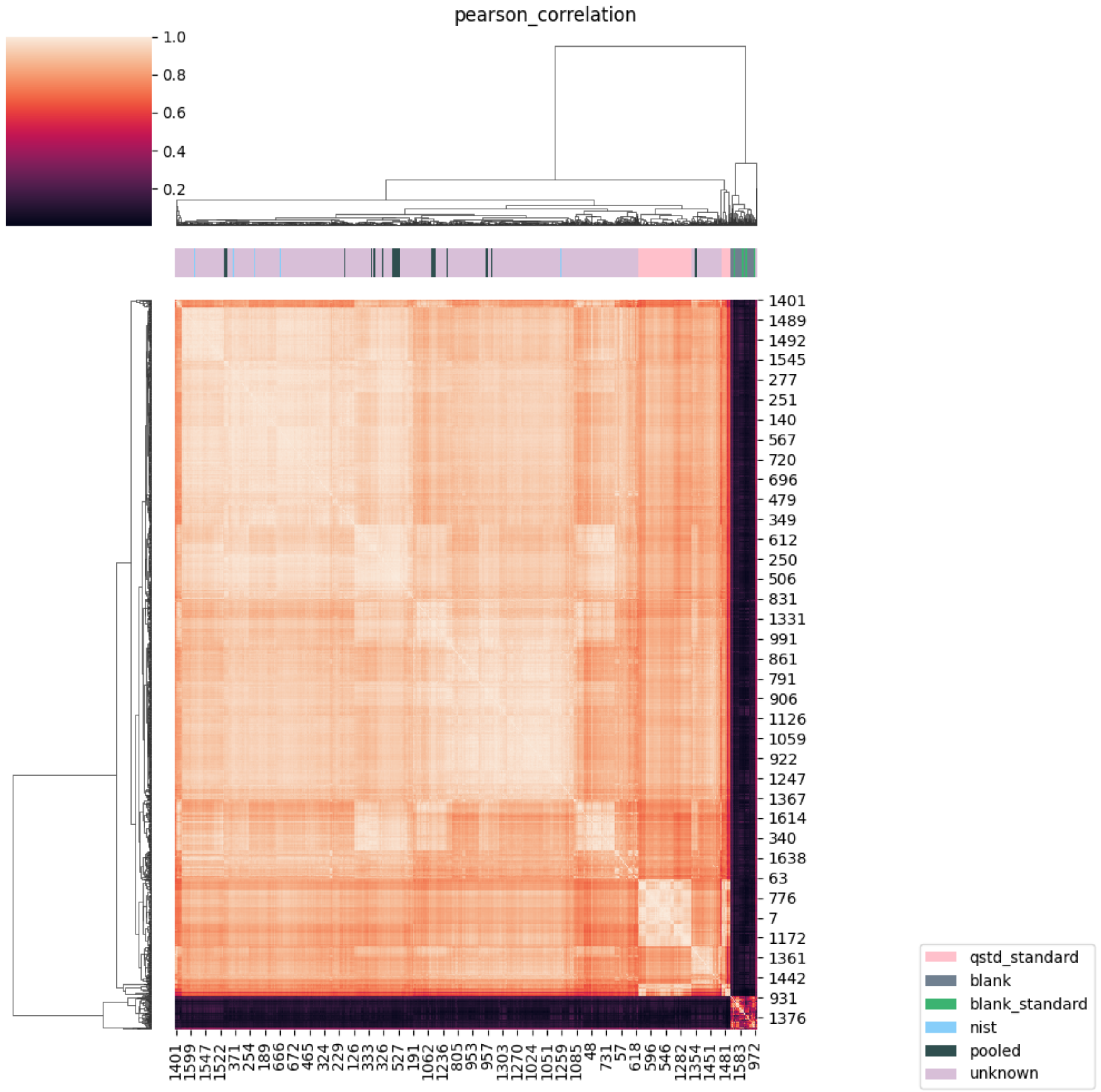

The pearson correlation represents how similar samples in feature intensities but assumes linearity. Higher correlation typically implies more similarity in samples; however, if many features are missing and have zero values, this correlation will appear falsely high. More informative correlation will be capture after imputing missing values and log transforming.

PCPFM Report - HZV029_plasma_HILIC_pos

Table: preferred_blank_masked Figure: pearson_correlation

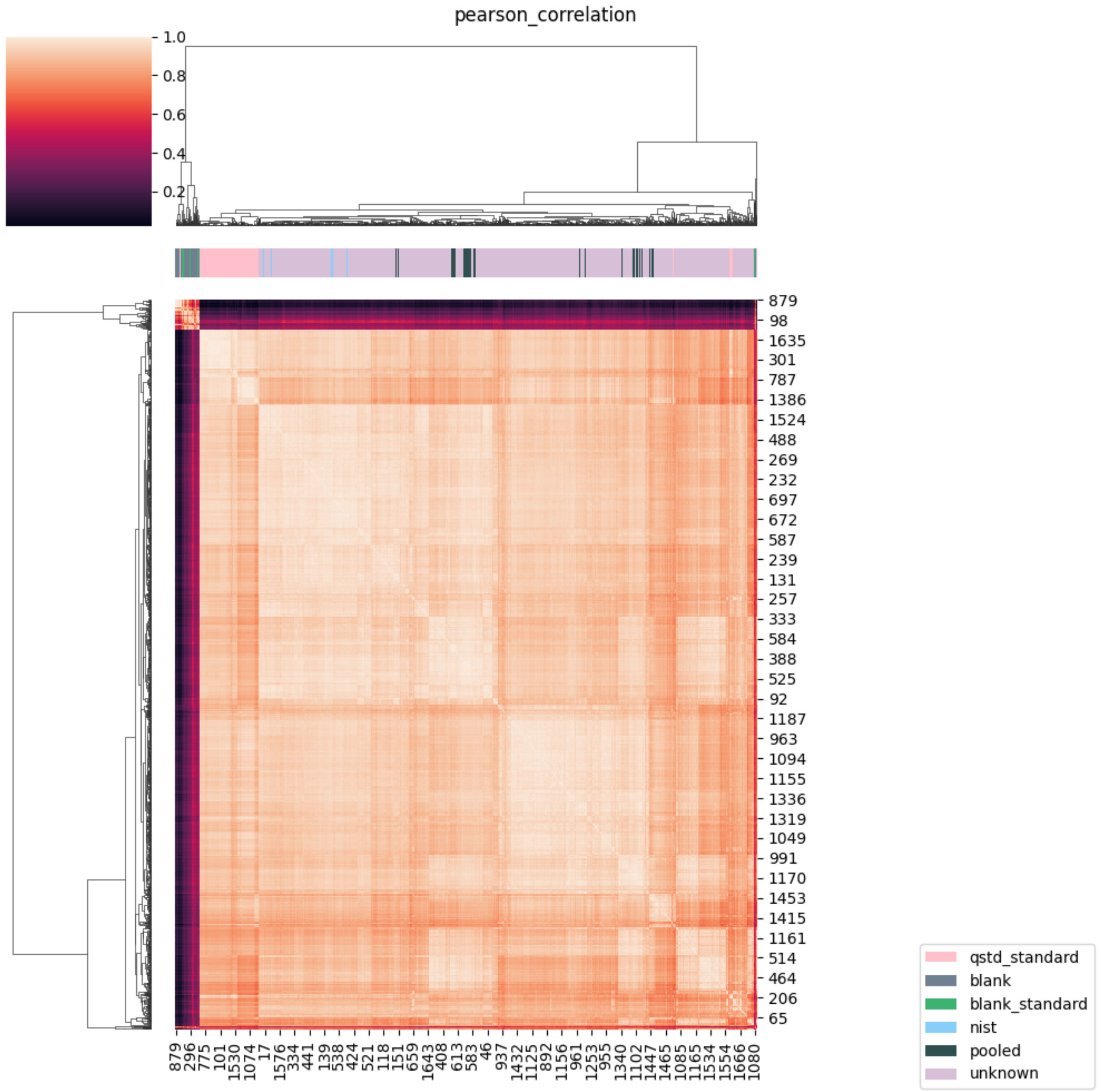

The pearson correlation represents how similar samples in feature intensities but assumes linearity. Higher correlation typically implies more similarity in samples; however, if many features are missing and have zero values, this correlation will appear falsely high. More informative correlation will be capture after imputing missing values and log transforming.

PCPFM Report - HZV029_plasma_HILIC_pos

Table: masked_preferred_unknowns Figure: pearson_correlation

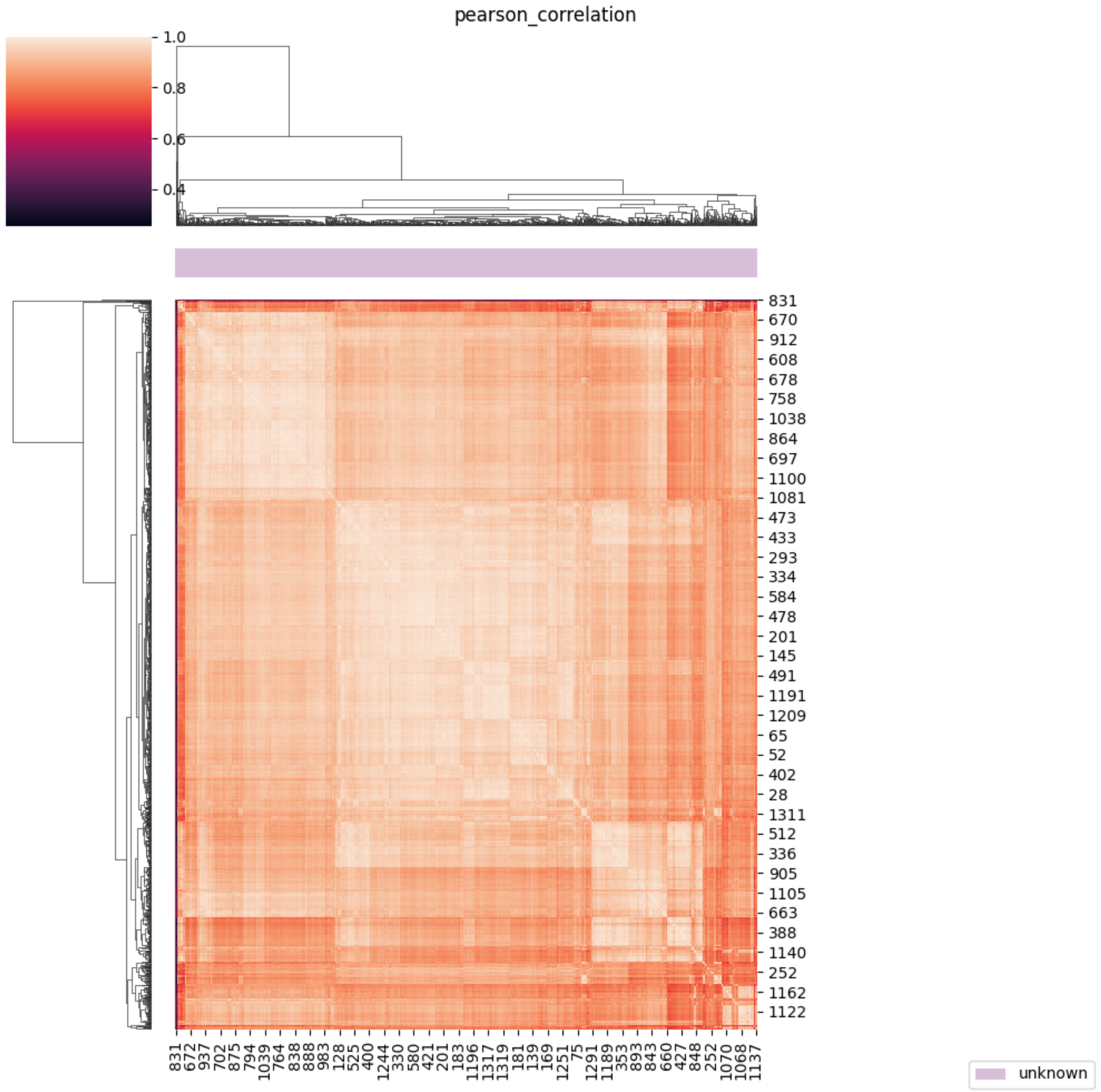

The pearson correlation represents how similar samples in feature intensities but assumes linearity. Higher correlation typically implies more similarity in samples; however, if many features are missing and have zero values, this correlation will appear falsely high. More informative correlation will be capture after imputing missing values and log transforming.

PCPFM Report - HZV029_plasma_HILIC_pos

Table: qaqc_filtered_masked_pref_unknowns Figure: pearson_correlation

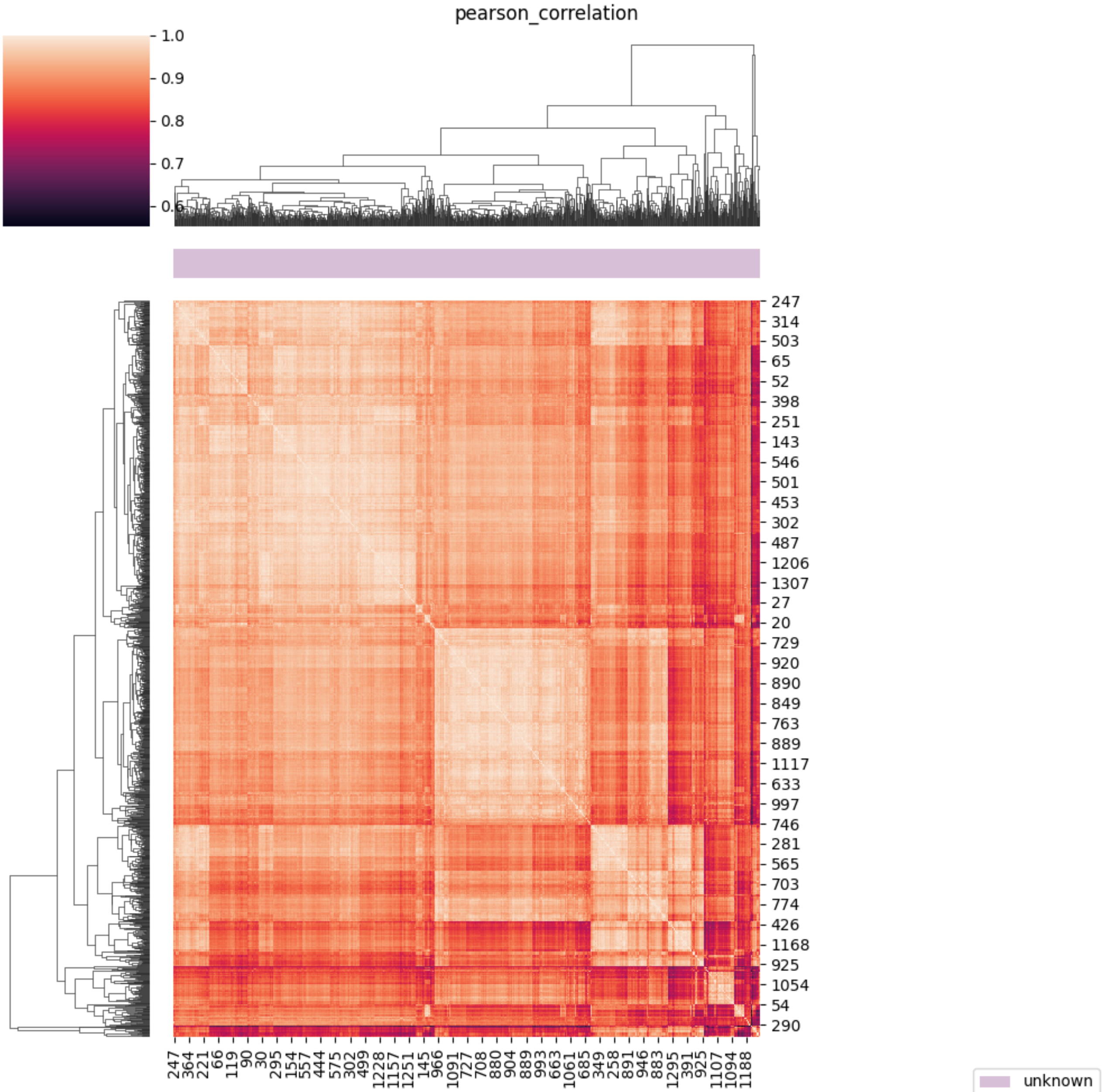

The pearson correlation represents how similar samples in feature intensities but assumes linearity. Higher correlation typically implies more similarity in samples; however, if many features are missing and have zero values, this correlation will appear falsely high. More informative correlation will be capture after imputing missing values and log transforming.

PCPFM Report - HZV029_plasma_HILIC_pos

Table: pref_normalized Figure: pearson_correlation

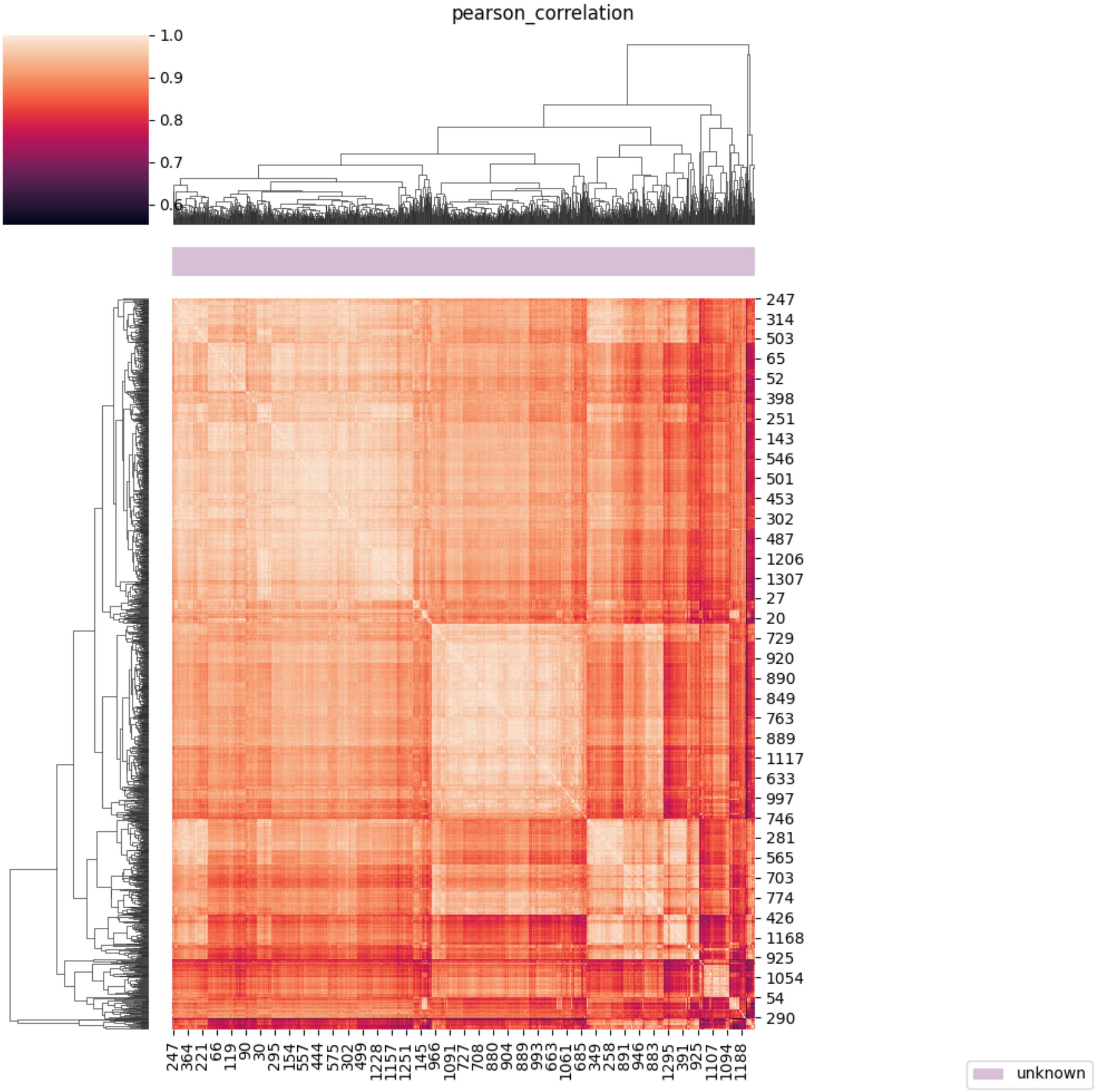

The pearson correlation represents how similar samples in feature intensities but assumes linearity. Higher correlation typically implies more similarity in samples; however, if many features are missing and have zero values, this correlation will appear falsely high. More informative correlation will be capture after imputing missing values and log transforming.

PCPFM Report - HZV029_plasma_HILIC_pos

Table: pref_dropped Figure: pearson_correlation

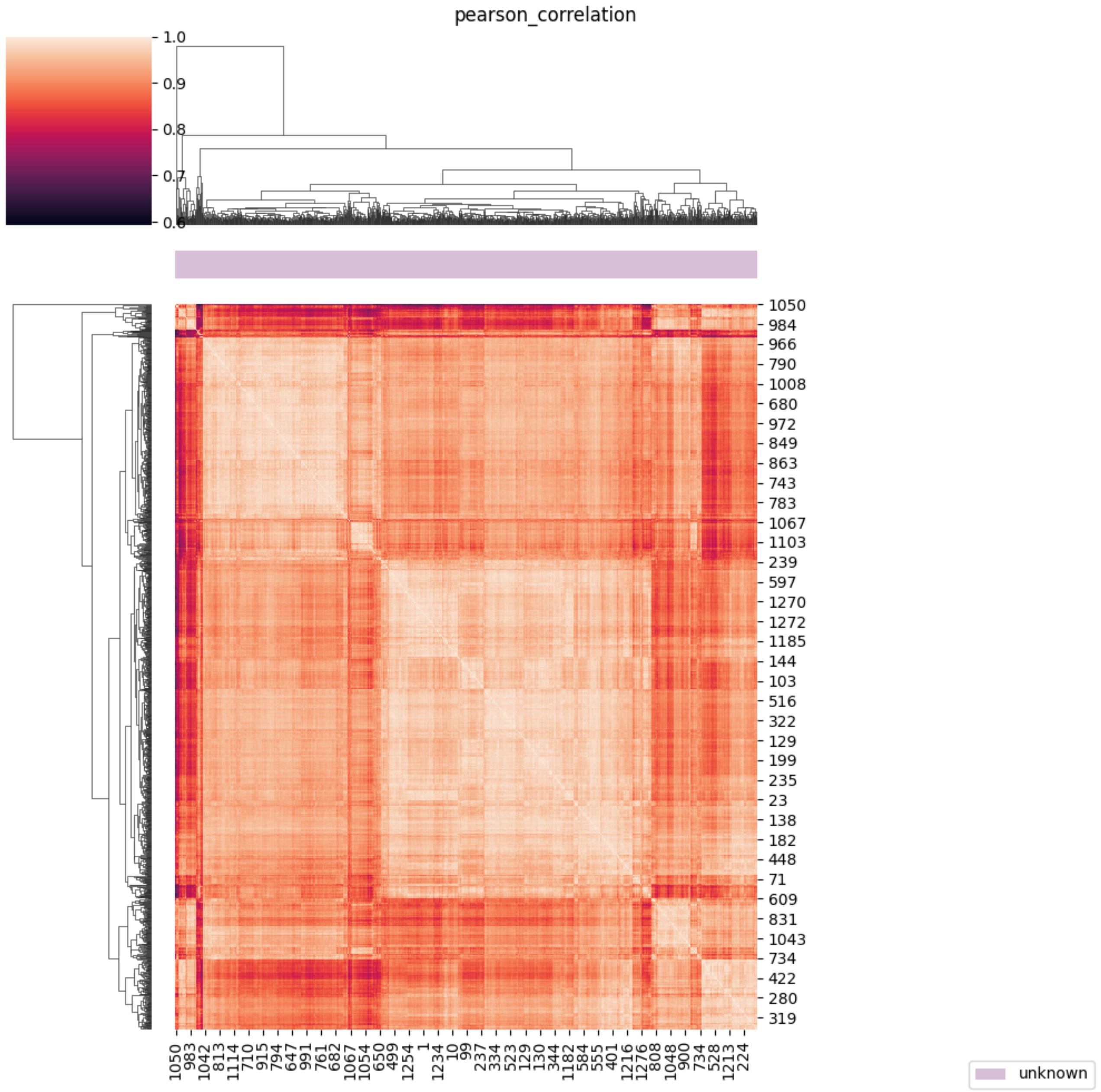

The pearson correlation represents how similar samples in feature intensities but assumes linearity. Higher correlation typically implies more similarity in samples; however, if many features are missing and have zero values, this correlation will appear falsely high. More informative correlation will be capture after imputing missing values and log transforming.

PCPFM Report - HZV029_plasma_HILIC_pos

Table: pref_interpolated Figure: pearson_correlation

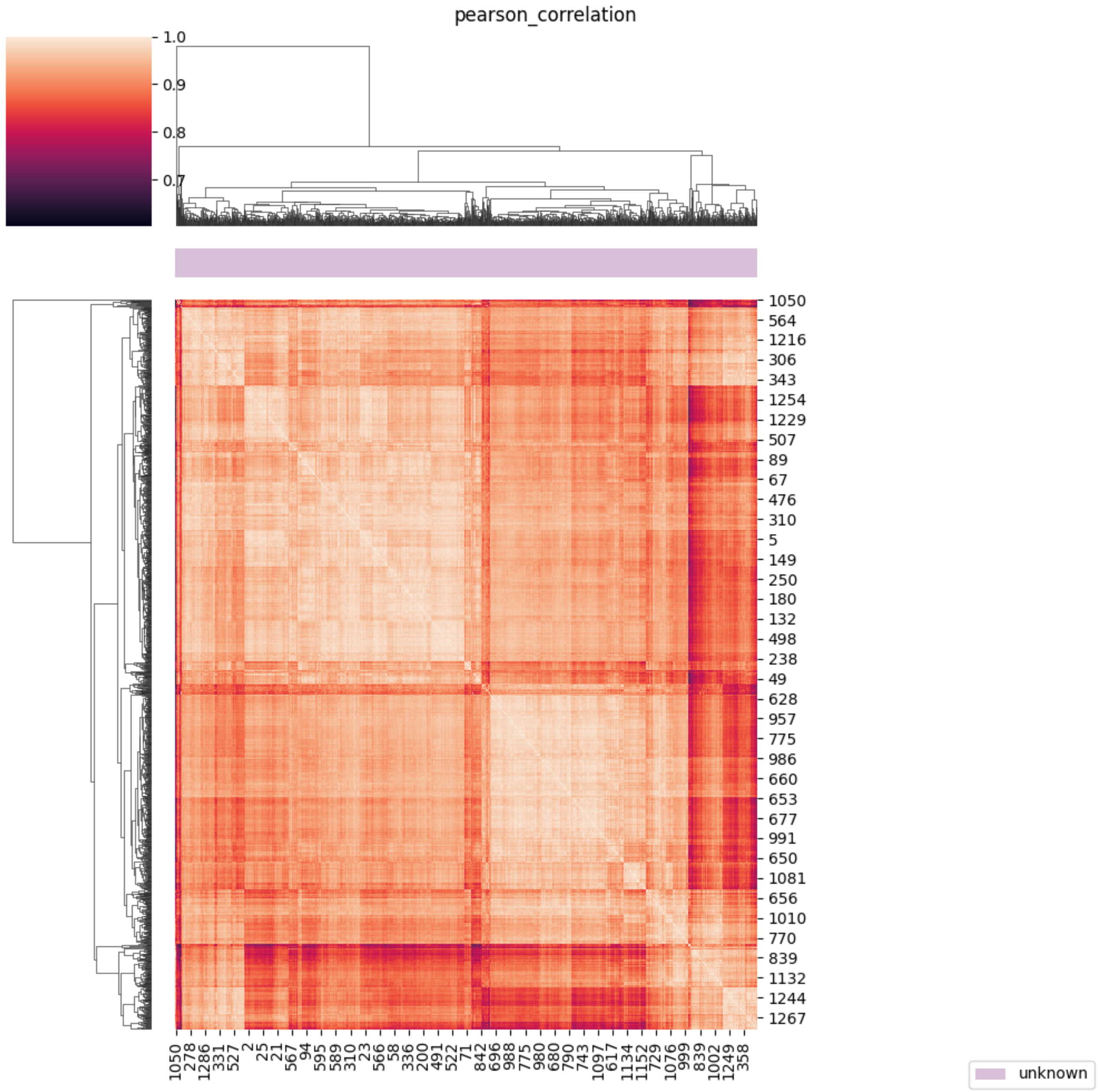

The pearson correlation represents how similar samples in feature intensities but assumes linearity. Higher correlation typically implies more similarity in samples; however, if many features are missing and have zero values, this correlation will appear falsely high. More informative correlation will be capture after imputing missing values and log transforming.

PCPFM Report - HZV029_plasma_HILIC_pos

Table: for_analysis Figure: pearson_correlation

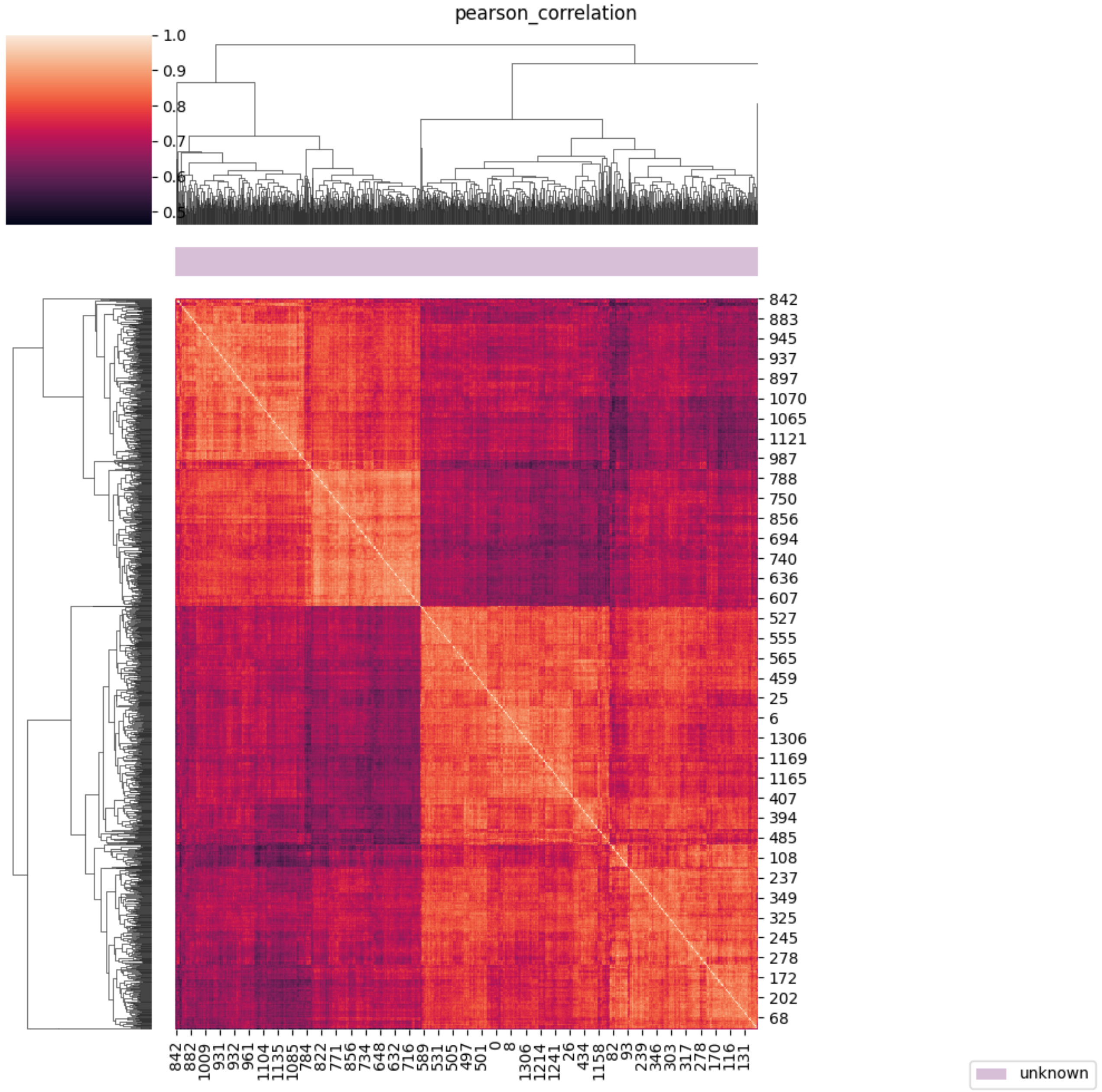

The pearson correlation represents how similar samples in feature intensities but assumes linearity. Higher correlation typically implies more similarity in samples; however, if many features are missing and have zero values, this correlation will appear falsely high. More informative correlation will be capture after imputing missing values and log transforming.

PCPFM Report - HZV029_plasma_HILIC_pos

Table: full Figure: log_tics

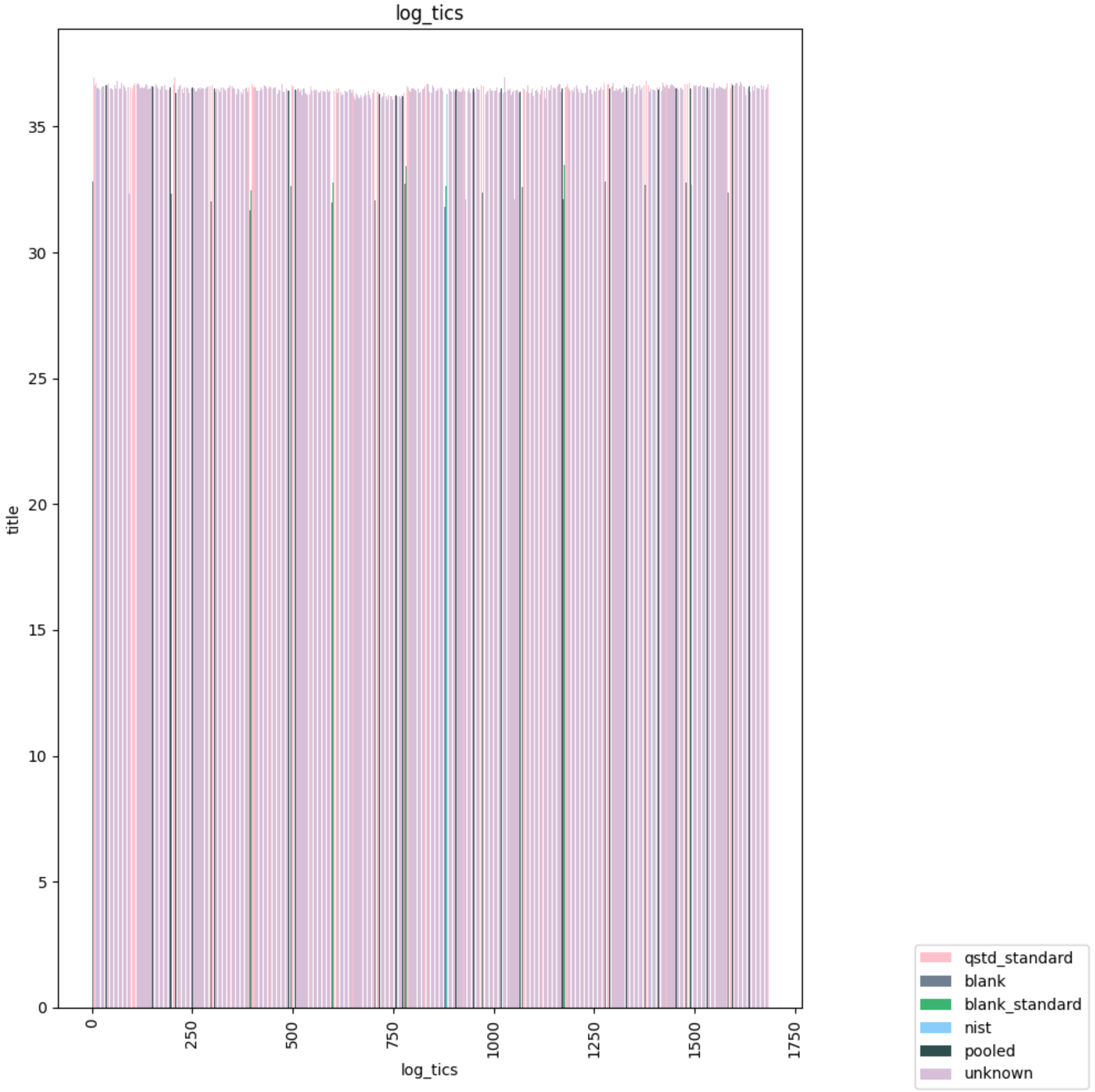

The sum of all ions in the feature table then log10 transformed. This is useful for finding all sorts of anomalies

PCPFM Report - HZV029_plasma_HILIC_pos

Table: preferred Figure: log_tics

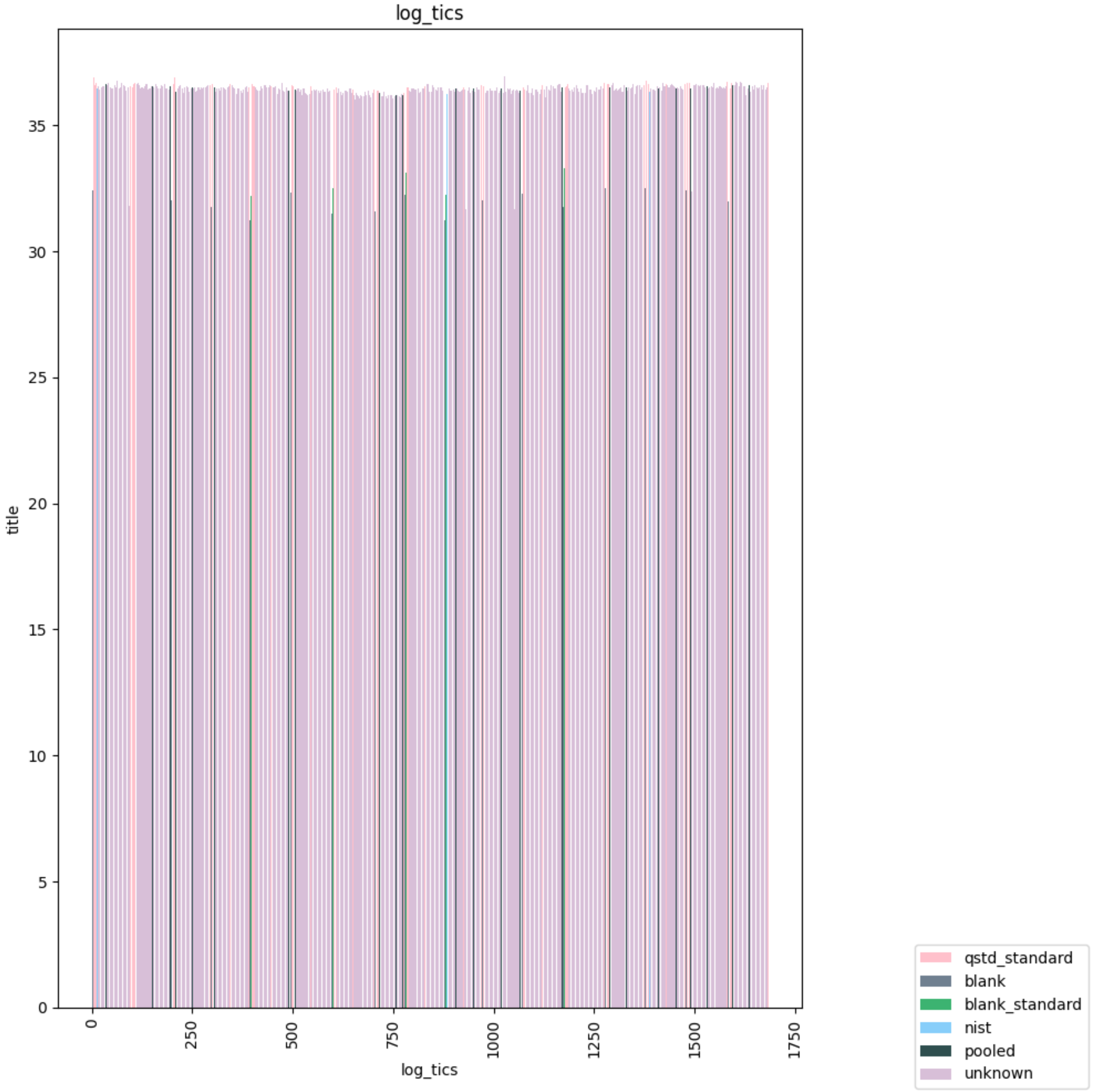

The sum of all ions in the feature table then log10 transformed. This is useful for finding all sorts of anomalies

PCPFM Report - HZV029_plasma_HILIC_pos

Table: preferred_blank_masked Figure: log_tics

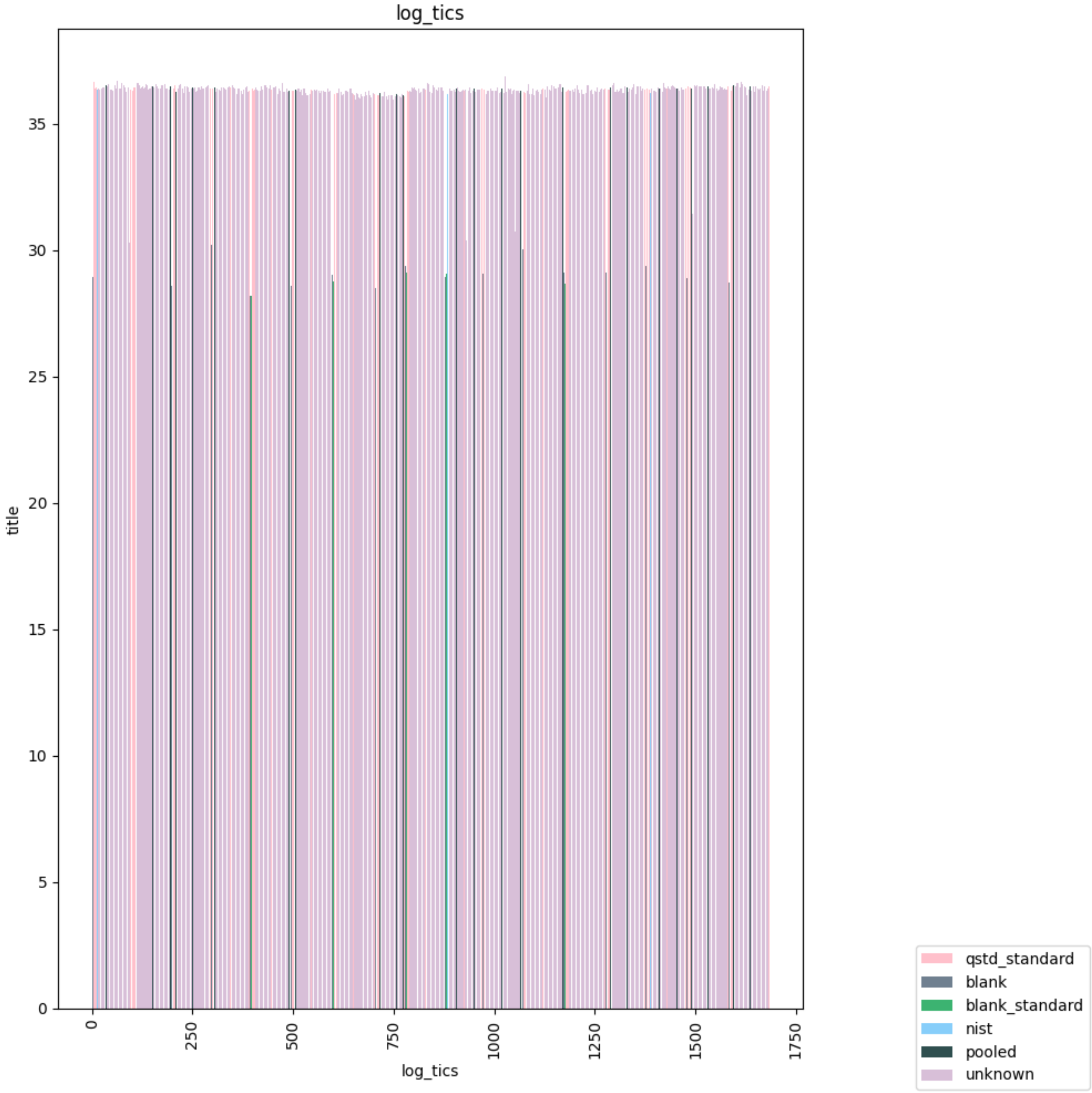

The sum of all ions in the feature table then log10 transformed. This is useful for finding all sorts of anomalies

PCPFM Report - HZV029_plasma_HILIC_pos

Table: masked_preferred_unknowns Figure: log_tics

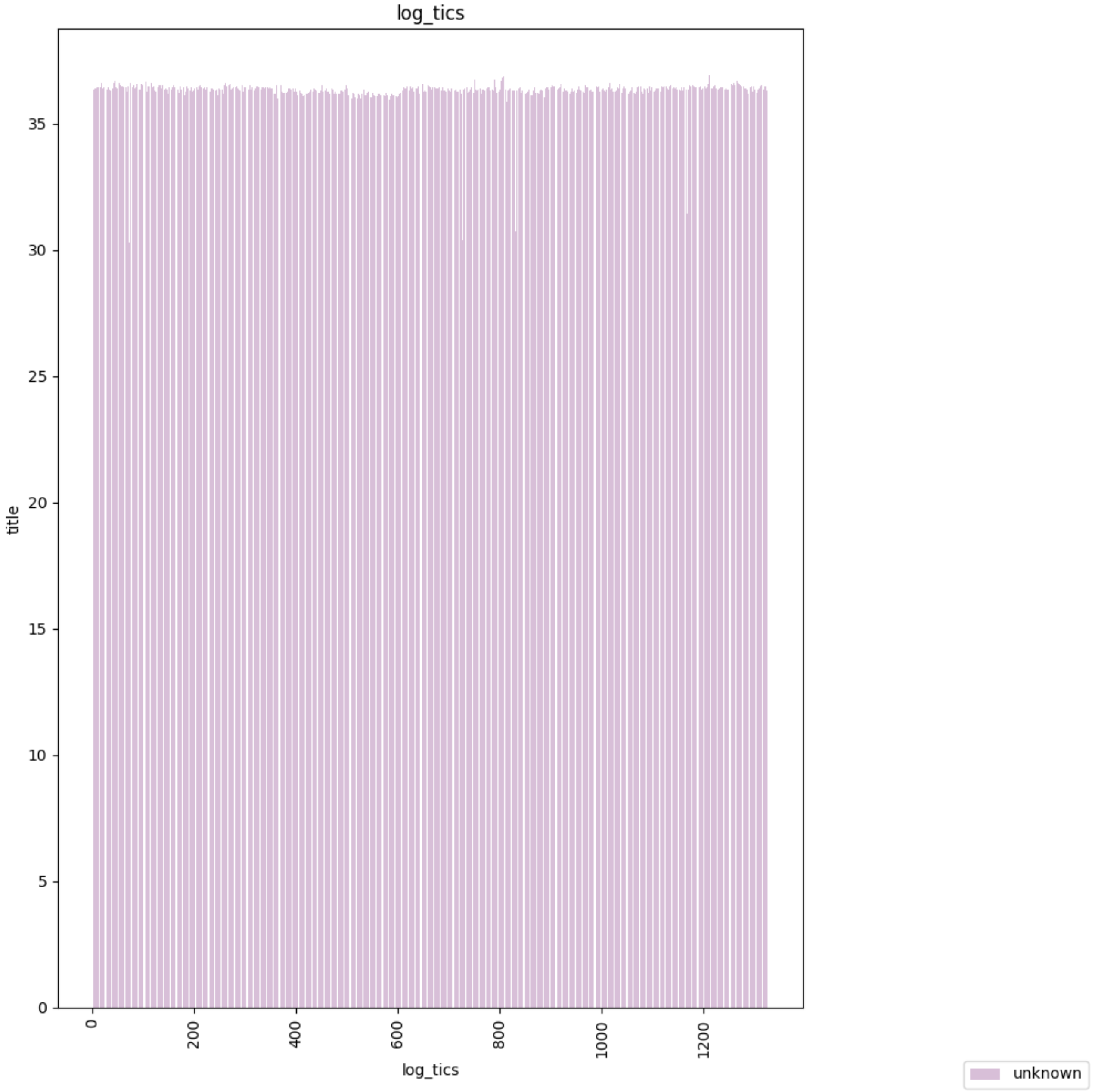

The sum of all ions in the feature table then log10 transformed. This is useful for finding all sorts of anomalies

PCPFM Report - HZV029_plasma_HILIC_pos

Table: qaqc_filtered_masked_pref_unknowns Figure: log_tics

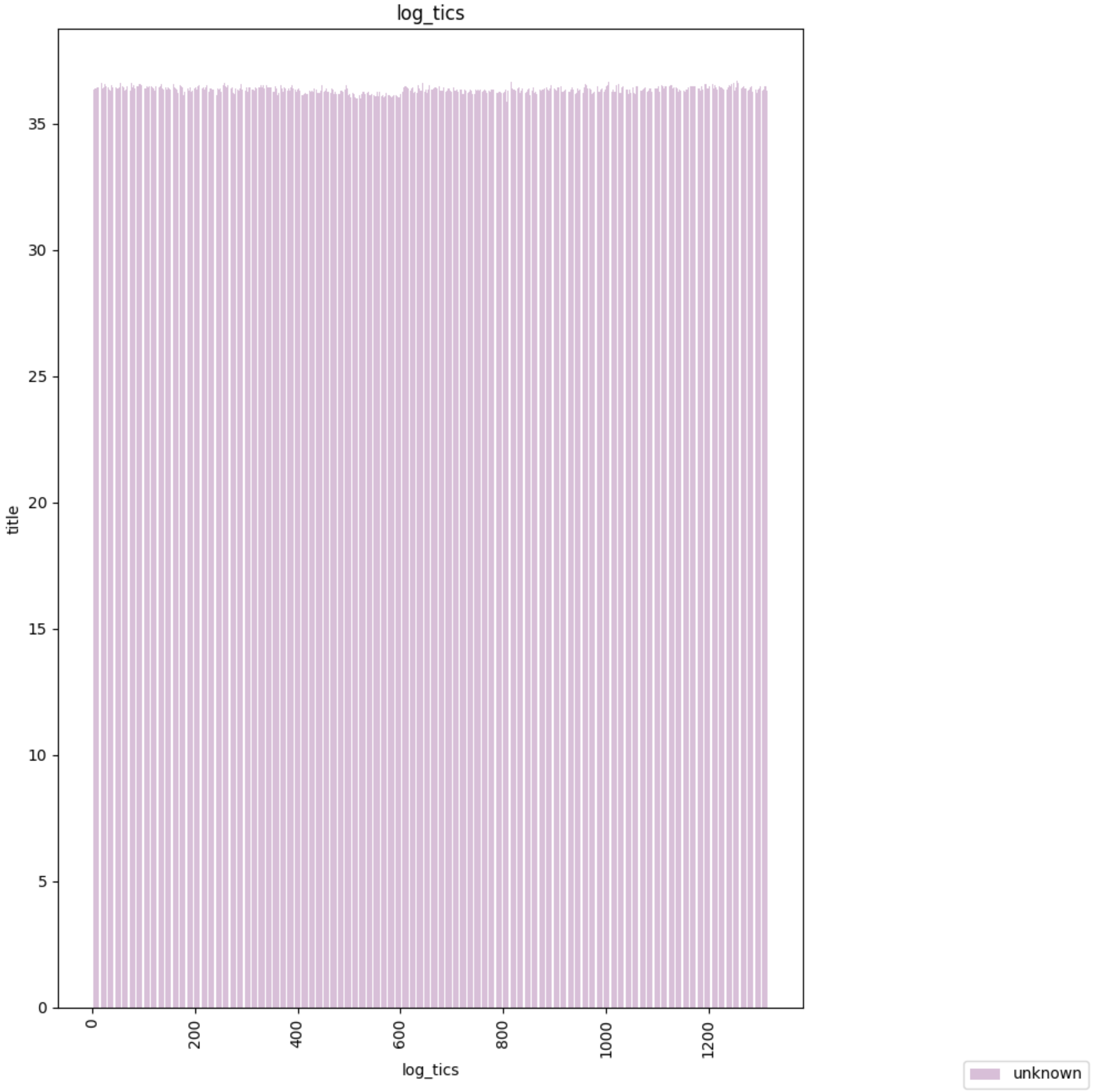

The sum of all ions in the feature table then log10 transformed. This is useful for finding all sorts of anomalies

PCPFM Report - HZV029_plasma_HILIC_pos

Table: pref_normalized Figure: log_tics

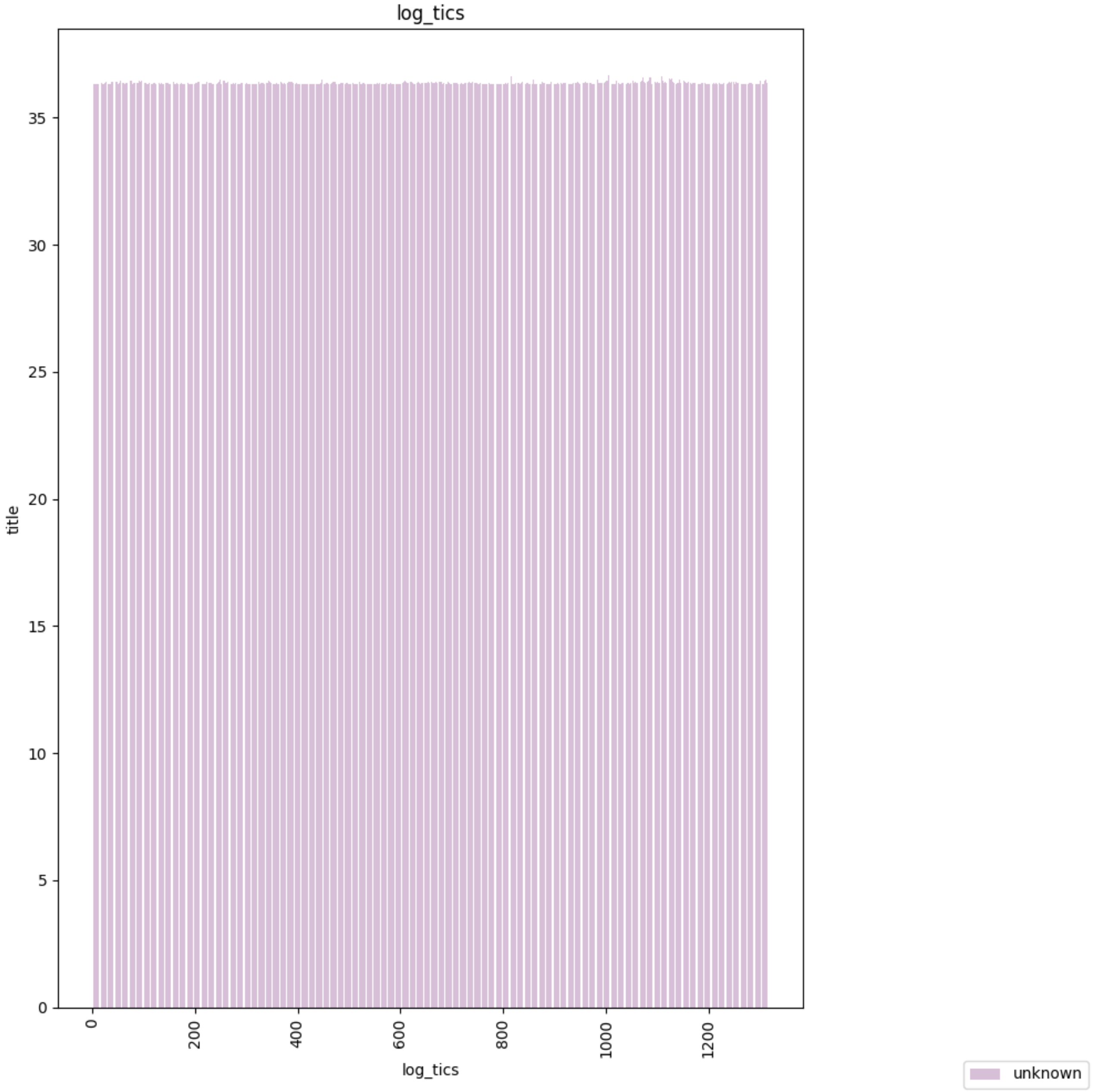

The sum of all ions in the feature table then log10 transformed. This is useful for finding all sorts of anomalies

PCPFM Report - HZV029_plasma_HILIC_pos

Table: pref_dropped Figure: log_tics

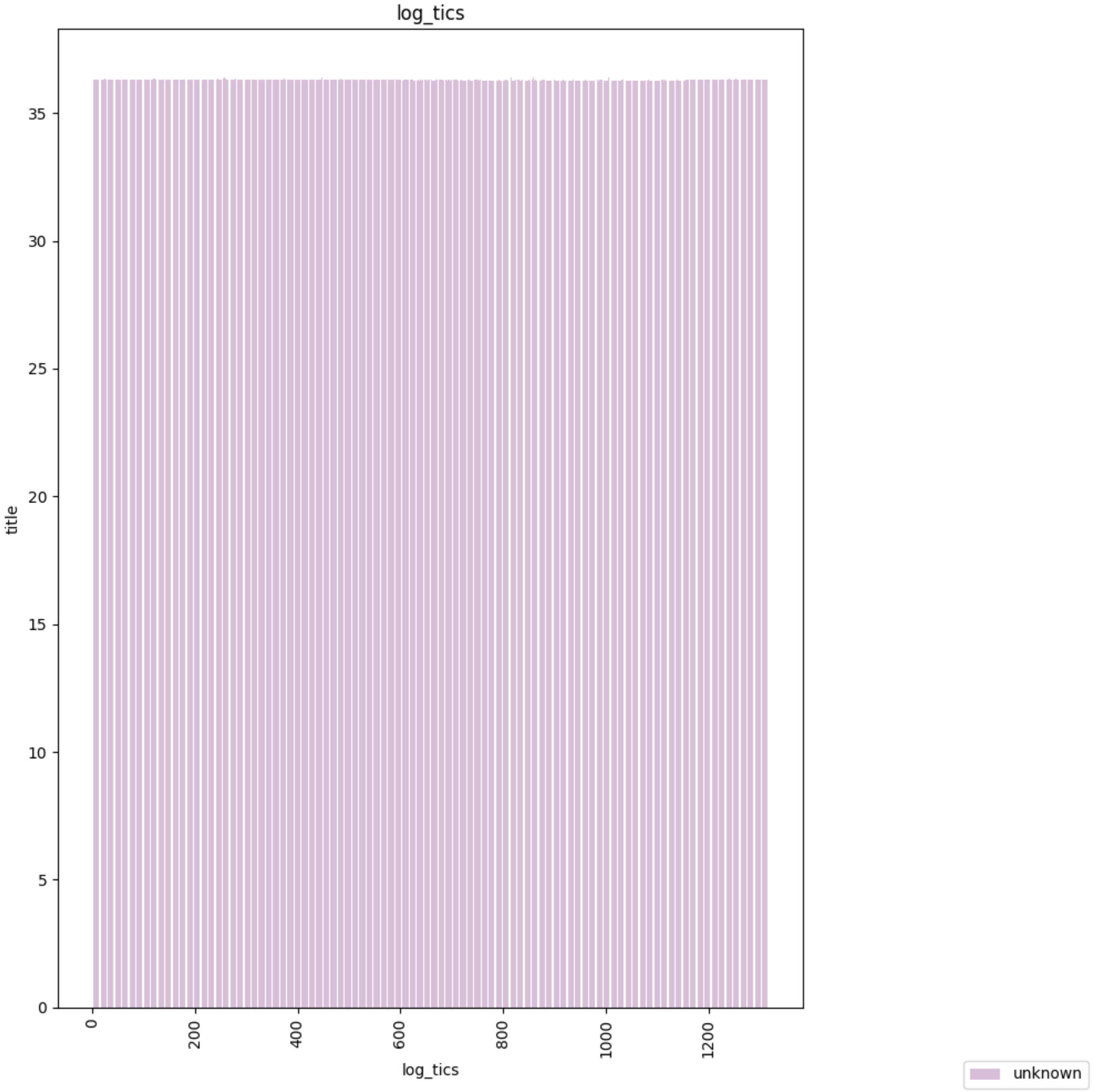

The sum of all ions in the feature table then log10 transformed. This is useful for finding all sorts of anomalies

PCPFM Report - HZV029_plasma_HILIC_pos

Table: pref_interpolated Figure: log_tics

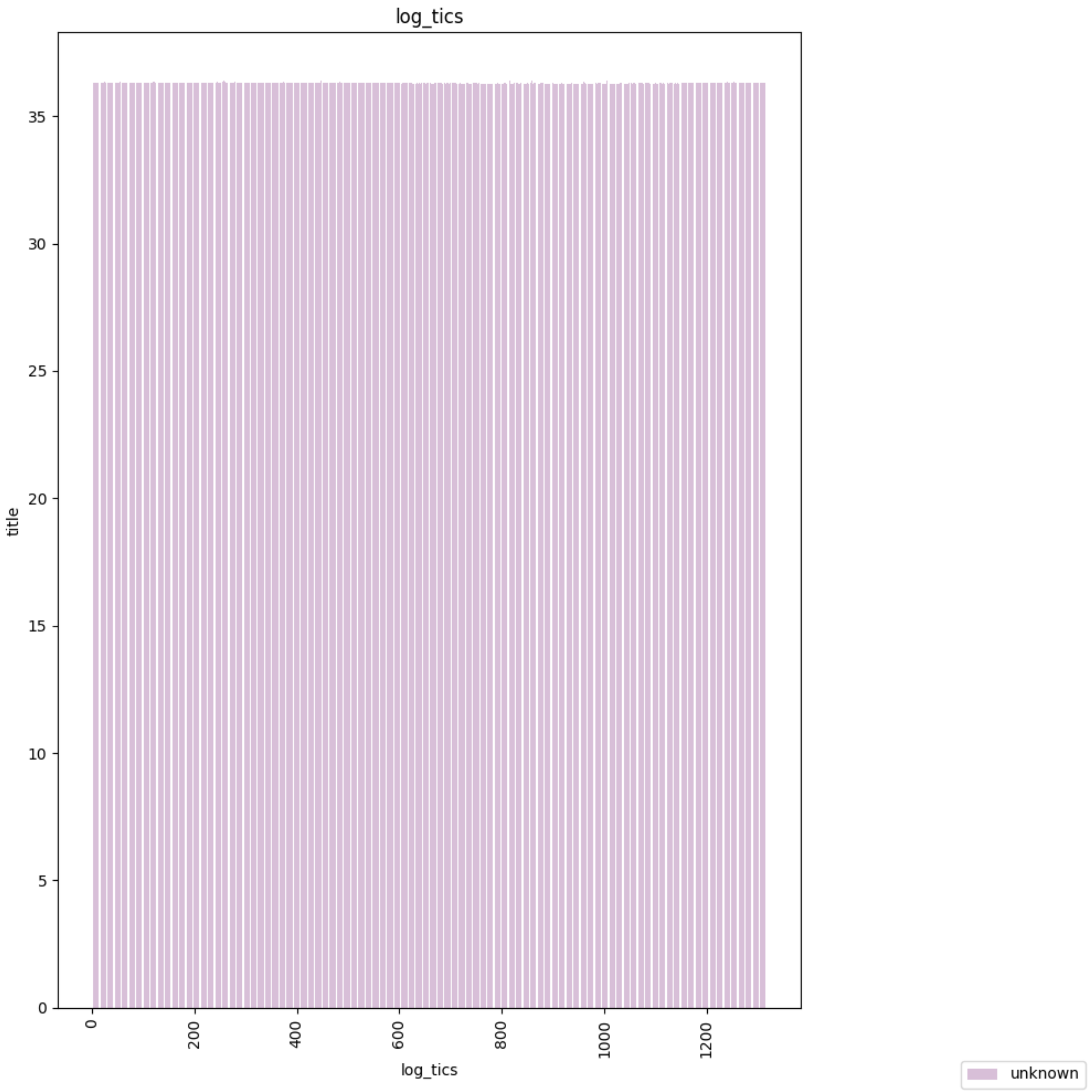

The sum of all ions in the feature table then log10 transformed. This is useful for finding all sorts of anomalies

PCPFM Report - HZV029_plasma_HILIC_pos

Table: for_analysis Figure: log_tics

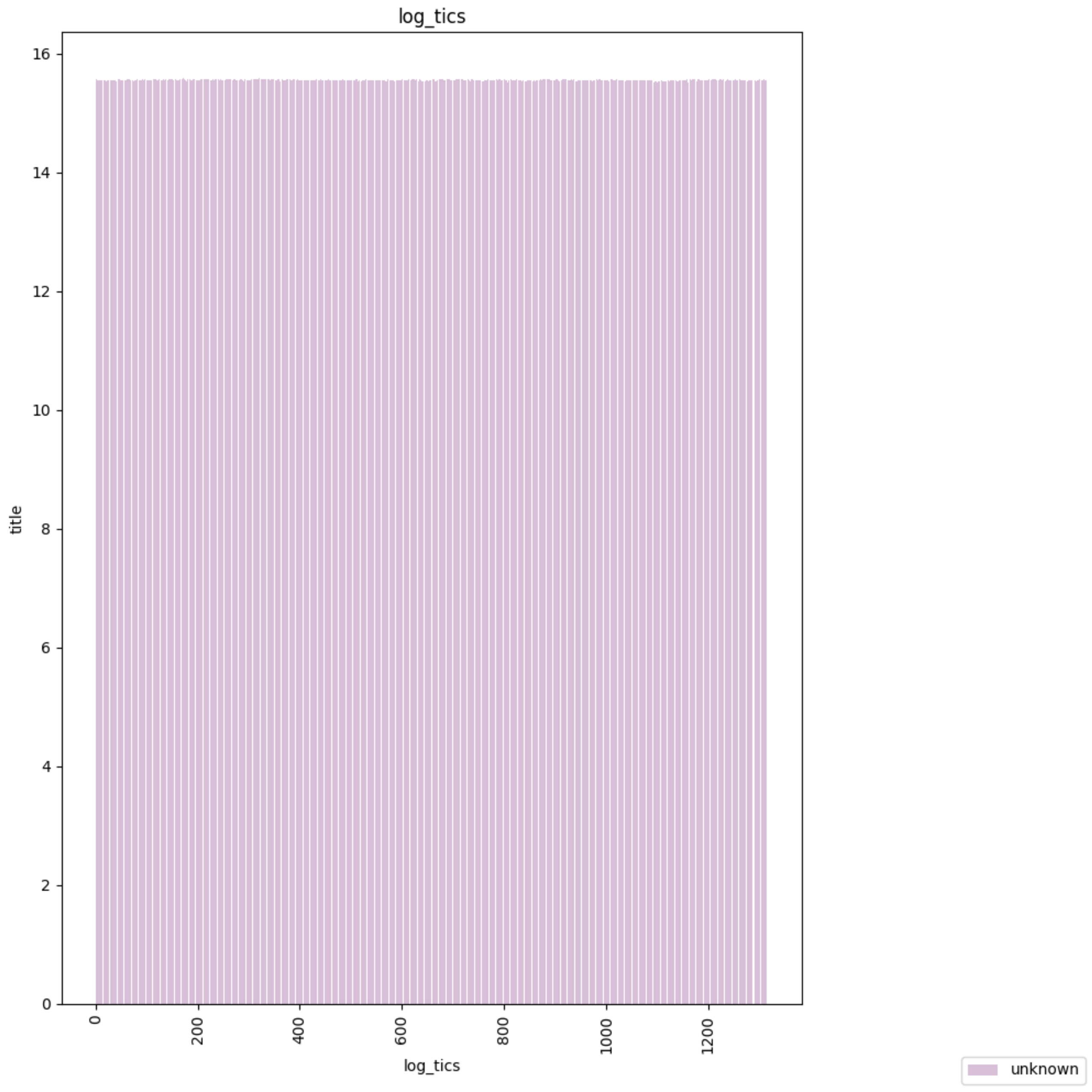

The sum of all ions in the feature table then log10 transformed. This is useful for finding all sorts of anomalies

PCPFM Report - HZV029_plasma_HILIC_pos

Table: full Figure: feature_count_z_scores

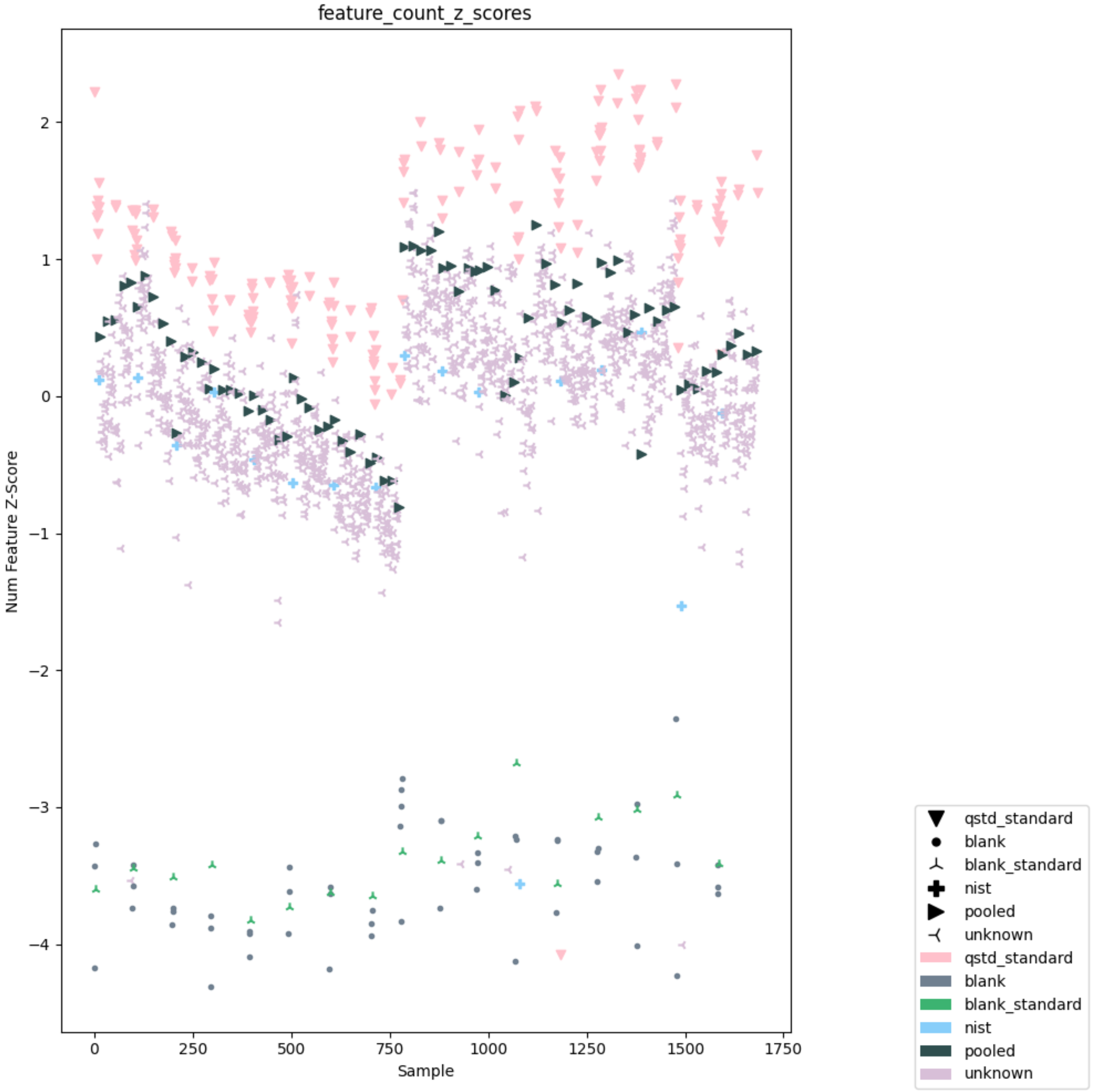

A z-score calculated using the standard deviation and median number of features per sample in the feature table. This is useful for finding failed injections and other anomalies.

PCPFM Report - HZV029_plasma_HILIC_pos

Table: preferred Figure: feature_count_z_scores

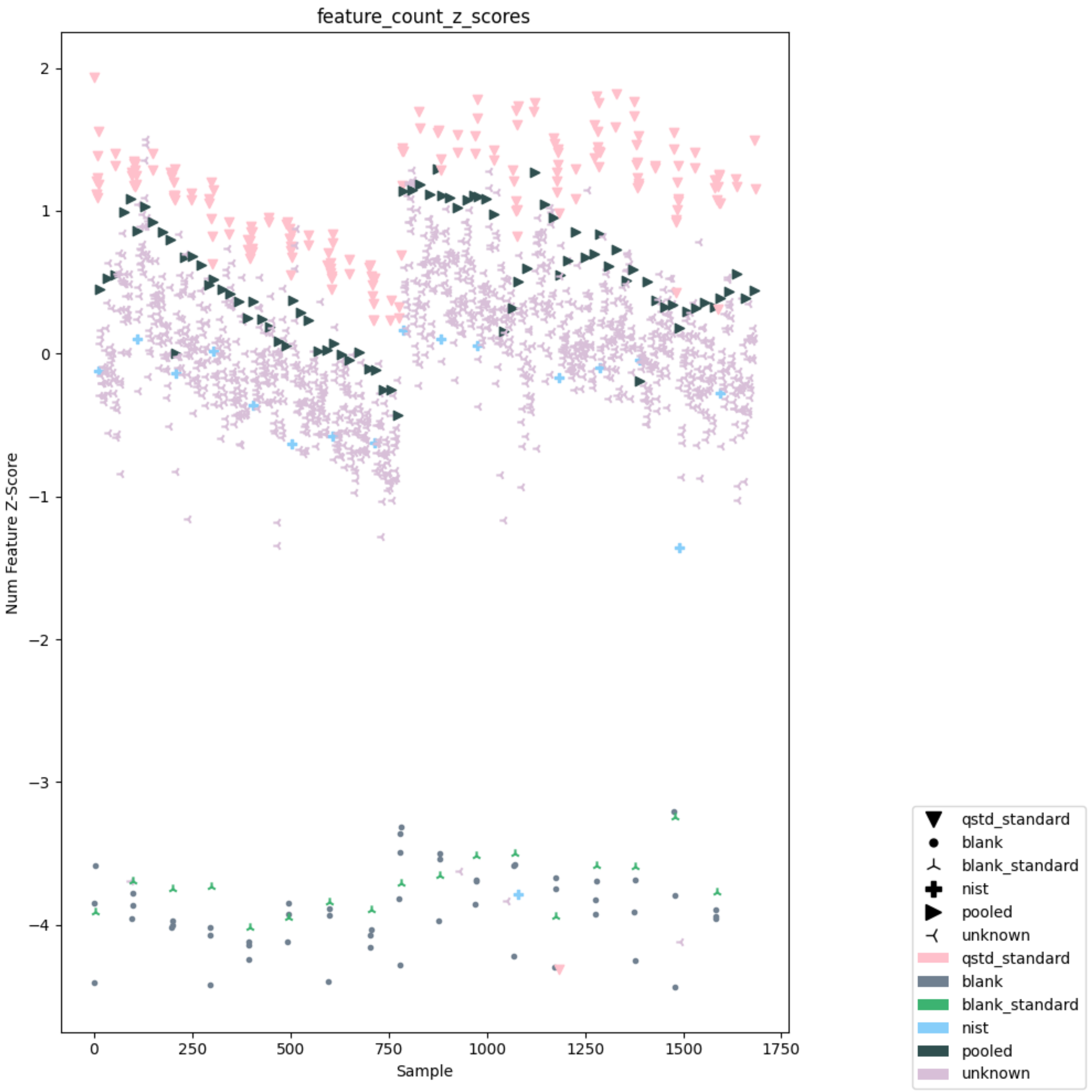

A z-score calculated using the standard deviation and median number of features per sample in the feature table. This is useful for finding failed injections and other anomalies.

PCPFM Report - HZV029_plasma_HILIC_pos

Table: preferred_blank_masked Figure: feature_count_z_scores

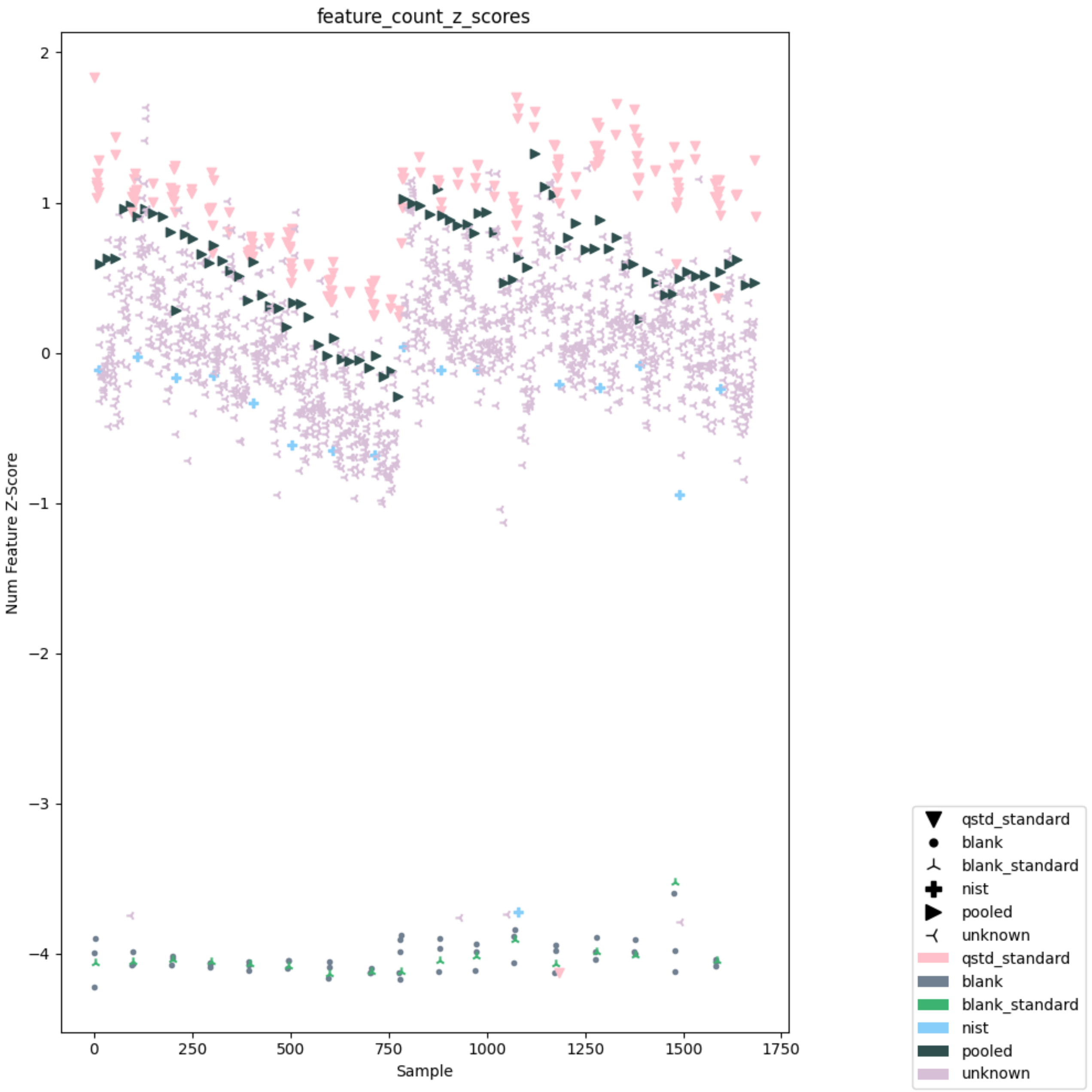

A z-score calculated using the standard deviation and median number of features per sample in the feature table. This is useful for finding failed injections and other anomalies.

PCPFM Report - HZV029_plasma_HILIC_pos

Table: masked_preferred_unknowns Figure: feature_count_z_scores

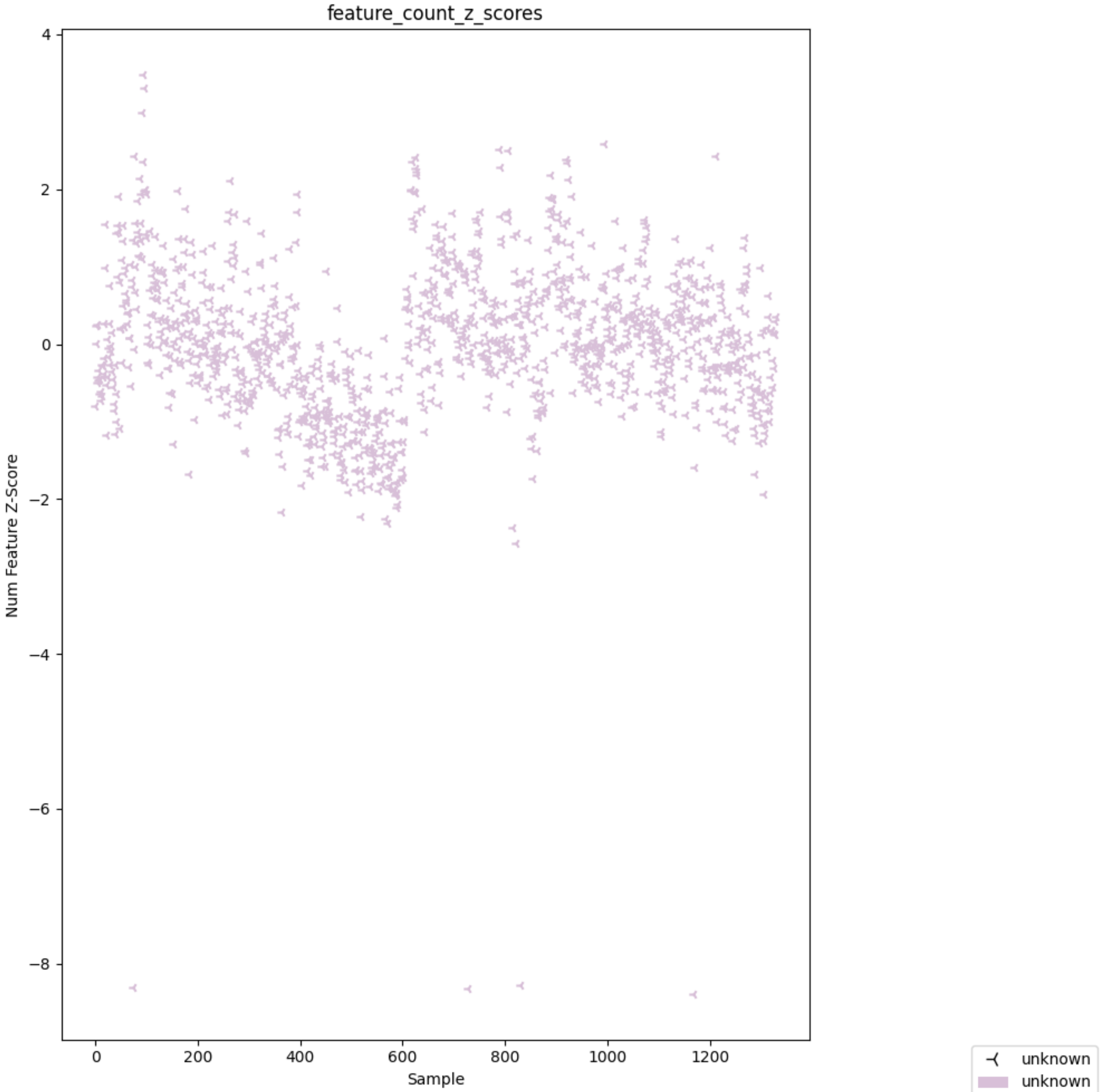

A z-score calculated using the standard deviation and median number of features per sample in the feature table. This is useful for finding failed injections and other anomalies.

PCPFM Report - HZV029_plasma_HILIC_pos

Table: qaqc_filtered_masked_pref_unknowns Figure: feature_count_z_scores

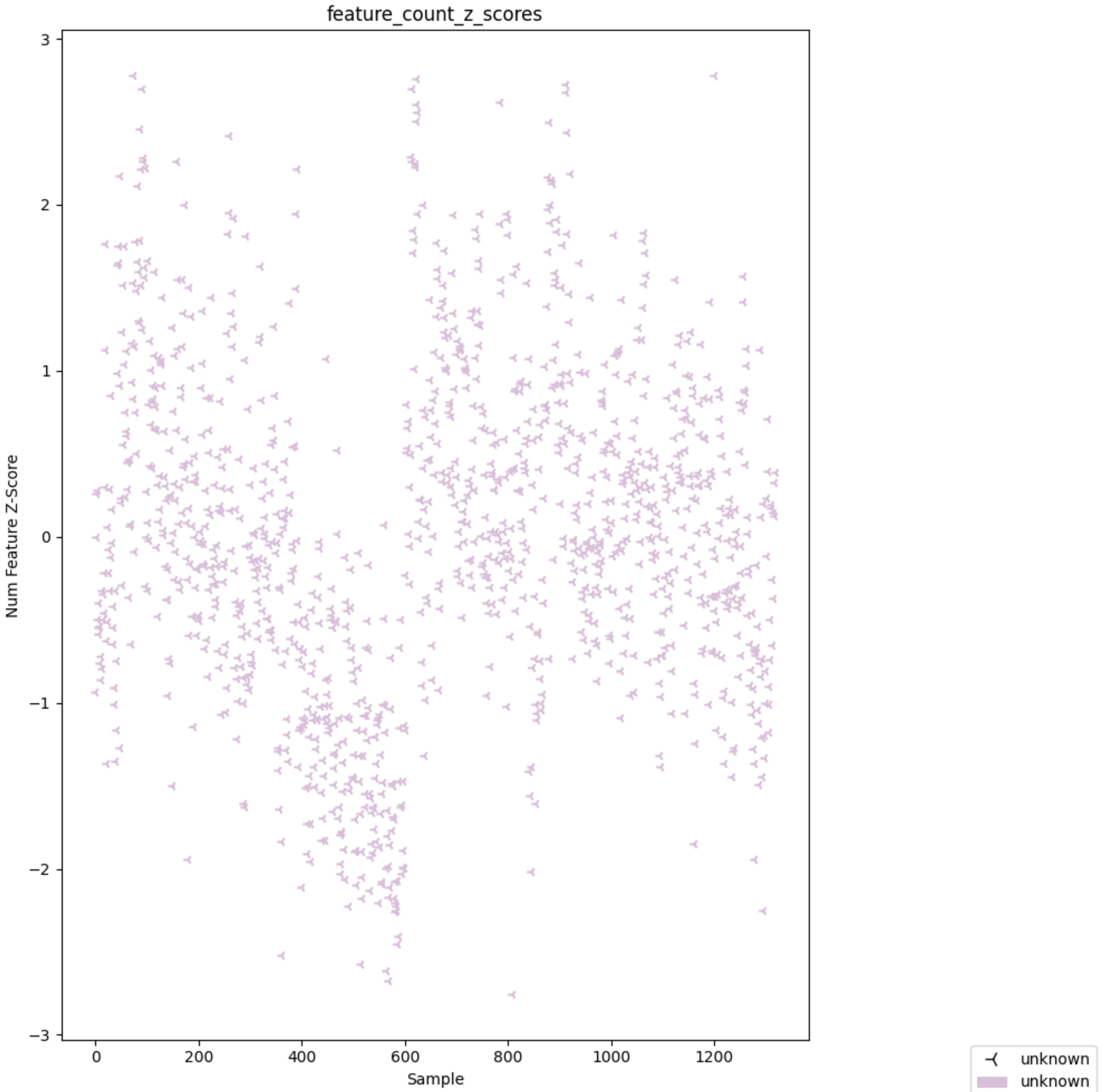

A z-score calculated using the standard deviation and median number of features per sample in the feature table. This is useful for finding failed injections and other anomalies.

PCPFM Report - HZV029_plasma_HILIC_pos

Table: pref_normalized Figure: feature_count_z_scores

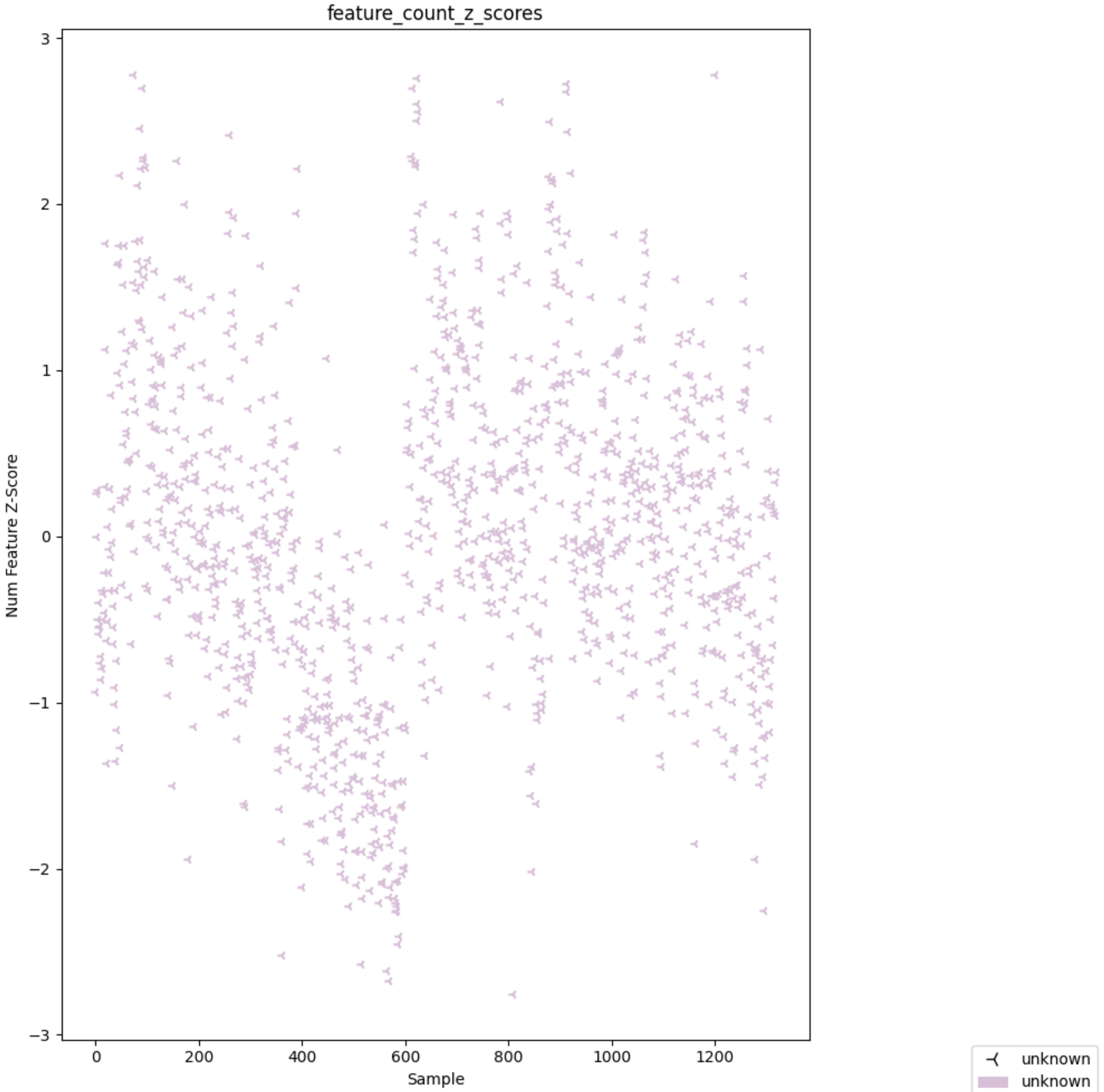

A z-score calculated using the standard deviation and median number of features per sample in the feature table. This is useful for finding failed injections and other anomalies.

PCPFM Report - HZV029_plasma_HILIC_pos

Table: pref_dropped Figure: feature_count_z_scores

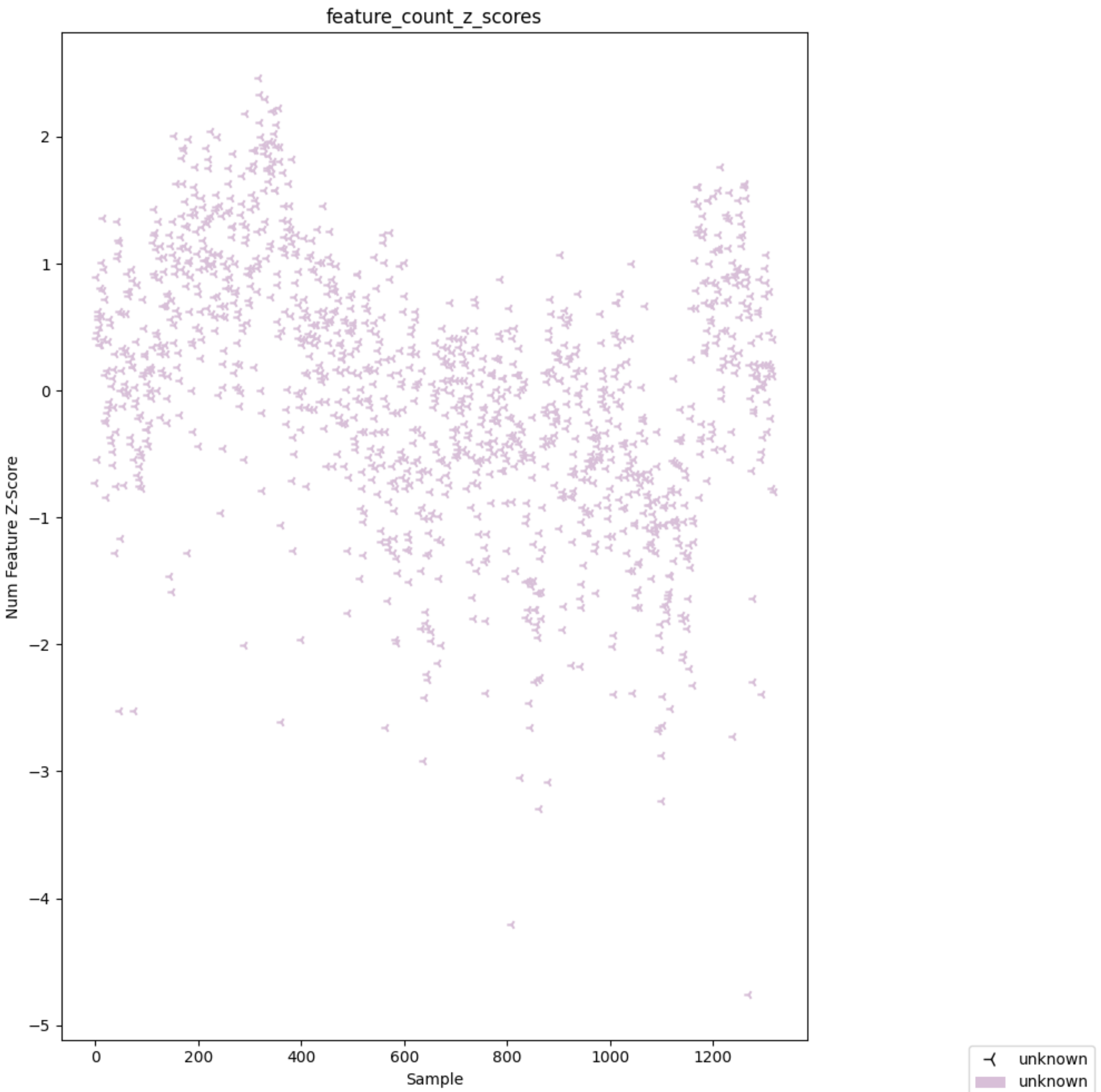

A z-score calculated using the standard deviation and median number of features per sample in the feature table. This is useful for finding failed injections and other anomalies.

PCPFM Report - HZV029_plasma_HILIC_pos

Table: pref_interpolated Figure: feature_count_z_scores

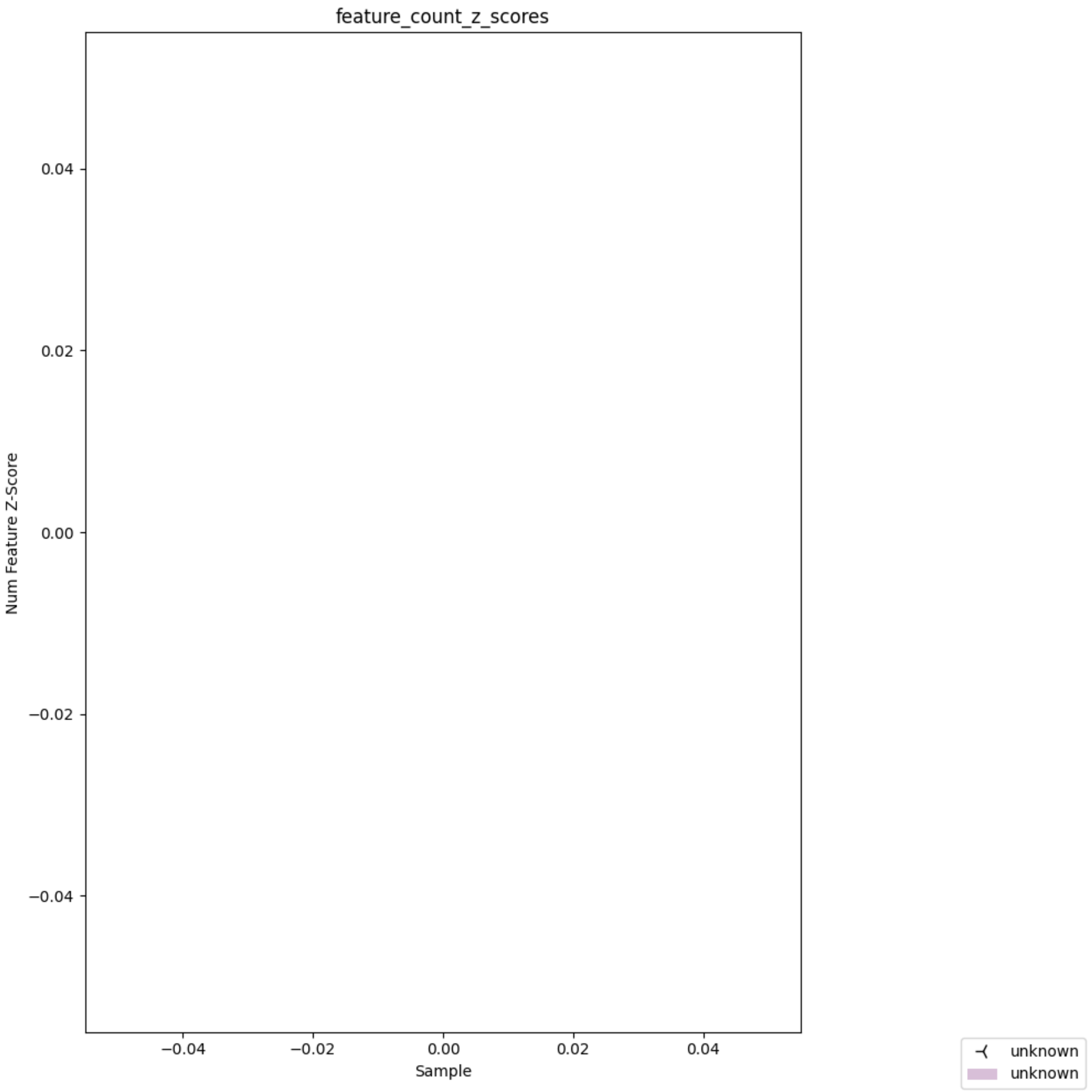

A z-score calculated using the standard deviation and median number of features per sample in the feature table. This is useful for finding failed injections and other anomalies.

PCPFM Report - HZV029_plasma_HILIC_pos

Table: for_analysis Figure: feature_count_z_scores

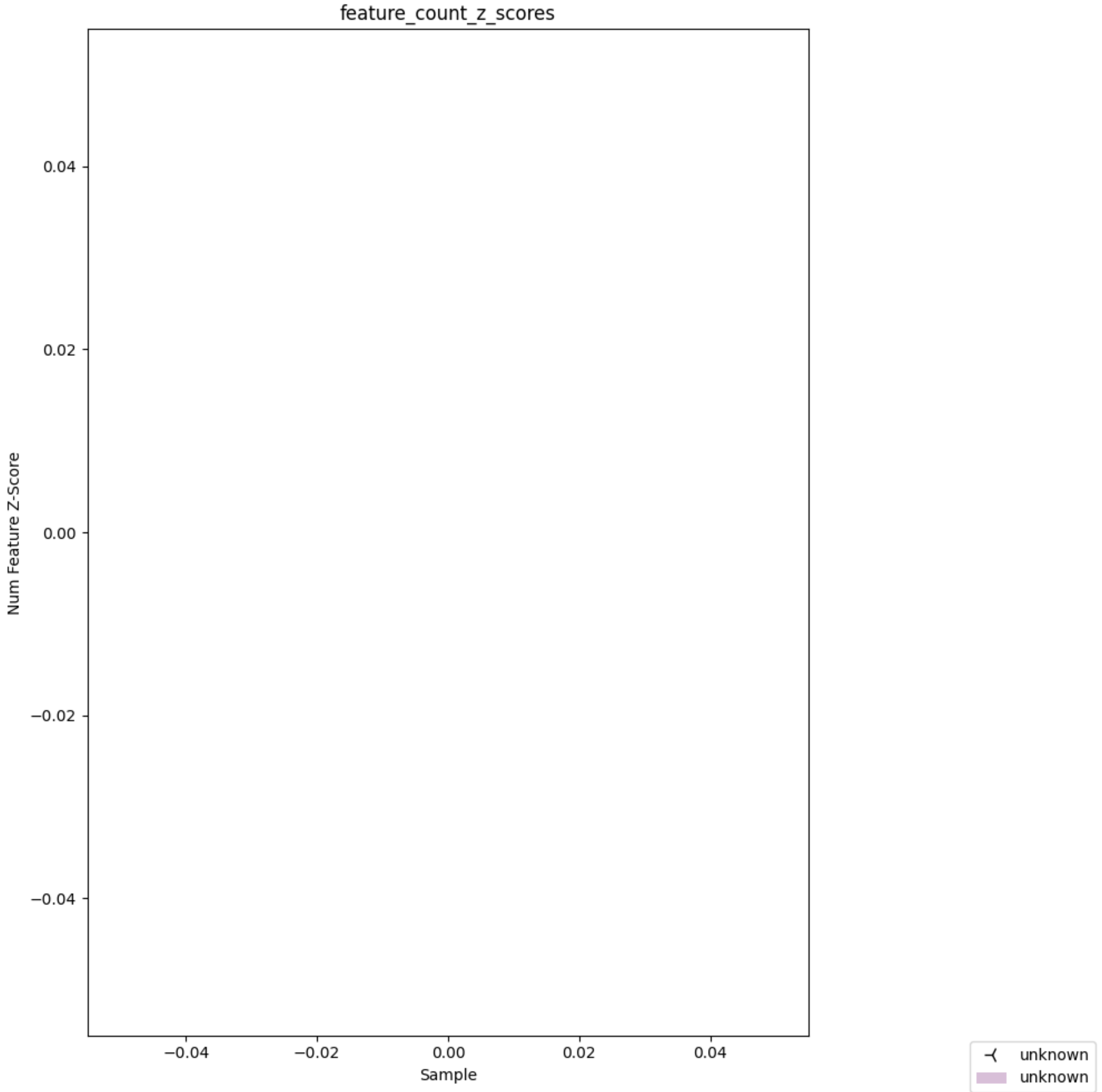

A z-score calculated using the standard deviation and median number of features per sample in the feature table. This is useful for finding failed injections and other anomalies.

Software Version Summary

pcpfm:1.0.13
seaborn:0.13.0
scipy:1.10.1
pymzml:2.5.2
mass2chem:0.4.9
matplotlib:3.8.2
matchms:0.23.1
metDataModel:0.6.0

PCPFM Report - HZV029_plasma_HILIC_pos

pycombat:0.20
gdown:4.7.1
intervaltree:3.1.0
scikit-learn:1.3.2
combat:0.3.3
numpy:1.24.4
jms-metabolite-services:0.5.7
asari-metabolomics:1.12.8
fpdf:1.7.2
setuptools:69.0.2
pandas:2.1.3
khipu-metabolomics:0.7.5

OS: Darwin
Python Version: 3.9.6
Architecture: arm64
Uname: Darwin MLG-JGM444 22.6.0 Darwin Kernel Version 22.6.0: Thu Nov 2 07:43:25 PDT 2023; root:xnu-8796.141.3.701.17~6/RELEASE_ARM64_T6020 arm64 arm

Command History

1712945005.027071:start_analysis

1712945006.173805:/Users/mitchjo/Library/Python/3.9/bin/pcpfm;assemble;-o;/Users/mitchjo/Analyses;-j;HZV029_plasma_HILIC_pos;-s;./sequence_files/HZV029_Plasma_w_reruns.csv;--filter;../../../filters/hilicpos.json;--name_field;File Name

1712945006.245746:/Users/mitchjo/Library/Python/3.9/bin/pcpfm;assemble;-o;/Users/mitchjo/Analyses;-j;HZV029_plasma_HILIC_pos;-s;./sequence_files/HZV029_Plasma_w_reruns.csv;--filter;../../../filters/hilicpos.json;--name_field;File Name

1712946247.679151:/Users/mitchjo/Library/Python/3.9/bin/pcpfm;asari;-i;/Users/mitchjo/Analyses/HZV029_plasma_HILIC_pos/

1712946353.960649:/Users/mitchjo/Library/Python/3.9/bin/pcpfm;blank_masking;--table_moniker;preferred;--new_moniker;preferred_blank_masked;--blank_value;blank;--sample_value;unknown;--query_field;Sample Type;--blank_intensity_ratio;3;-i;/Users/mitchjo/Analyses/HZV029_plasma_HILIC_pos/

1712946413.387945:/Users/mitchjo/Library/Python/3.9/bin/pcpfm;drop_samples;--table_moniker;preferred_blank_masked;--new_moniker;masked_preferred_unknowns;--drop_value;unknown;--drop_field;Sample Type;--drop_others;true;-i;/Users/mitchjo/Analyses/HZV029_plasma_HILIC_pos/

1712946470.1084101:/Users/mitchjo/Library/Python/3.9/bin/pcpfm;drop_outliers;--table_moniker;masked_preferred_unknowns;--new_moniker;qaqc_filtered_masked_pref_unknowns;-i;/Users/mitchjo/Anal

PCPFM Report - HZV029_plasma_HILIC_pos

yses/HZV029_plasma_HILIC_pos/

1712946536.6583319:/Users/mitchjo/Library/Python/3.9/bin/pcpfm;normalize;--table_moniker;qaqc_filtered_masked_pref_unknowns;--new_moniker;pref_normalized;--TIC_normalization_percentile;0.90;-i;/Users/mitchjo/Analyses/HZV029_plasma_HILIC_pos/

1712946548.63862:/Users/mitchjo/Library/Python/3.9/bin/pcpfm;drop_missing_features;-i;/Users/mitchjo/Analyses/HZV029_plasma_HILIC_pos/;-tm;pref_normalized;-nm;pref_dropped

1712946560.547254:/Users/mitchjo/Library/Python/3.9/bin/pcpfm;impute;-i;/Users/mitchjo/Analyses/HZV029_plasma_HILIC_pos/;--table_moniker;pref_dropped;--new_moniker;pref_interpolated

1712946571.7119439:/Users/mitchjo/Library/Python/3.9/bin/pcpfm;log_transform;-i;/Users/mitchjo/Analyses/HZV029_plasma_HILIC_pos/;--table_moniker;pref_interpolated;--new_moniker;for_analysis

1712946698.46121:/Users/mitchjo/Library/Python/3.9/bin/pcpfm;build_empCpds;-i;/Users/mitchjo/Analyses/HZV029_plasma_HILIC_pos/;-tm;full;-em;for_analysis;--add_singletons;true

1712946912.0258172:/Users/mitchjo/Library/Python/3.9/bin/pcpfm;map_ms2;-i;/Users/mitchjo/Analyses/HZV029_plasma_HILIC_pos/;-em;for_analysis;-nm;for_analysis2;--ms2_dir=/Users/mitchjo/Datasets/ForPCPFM/AcquireX_Datasets/Plasma_5_min_HILICpos/Pooled/

1712947059.6565602:/Users/mitchjo/Library/Python/3.9/bin/pcpfm;l4_annotate;-i;/Users/mitchjo/Analyses/HZV029_plasma_HILIC_pos/;-em;for_analysis2;-nm;HMDB_LMSD_annotated_for_analysis

1712947612.673506:/Users/mitchjo/Library/Python/3.9/bin/pcpfm;l2_annotate;-i;/Users/mitchjo/Analyses/HZV029_plasma_HILIC_pos/;-em;HMDB_LMSD_annotated_for_analysis;-nm;MoNA_HMDB_LMSD_
d_for_analysis

1712947803.022312:/Users/mitchjo/Library/Python/3.9/bin/pcpfm;report;-i;/Users/mitchjo/Analyses/HZV029_plasma_HILIC_pos/;--color_by=["Sample Type"];--marker_by=["Sample Type"]

1712947803.099259:/Users/mitchjo/Library/Python/3.9/bin/pcpfm;report;-i;/Users/mitchjo/Analyses/HZV029_plasma_HILIC_pos/;--color_by=["Sample Type"];--marker_by=["Sample Type"]

1712949123.740278:/Users/mitchjo/Library/Python/3.9/bin/pcpfm;finish;-i;/Users/mitchjo/Analyses/HZV029_plasma_HILIC_pos/
