# Supplementary material for: Common data models to streamline metabolomics processing and annotation, and implementation in a Python pipeline
Source: PLoS Comput Biol. 2024 Jun 6;20(6):e1011912. doi: 10.1371/journal.pcbi.1011912 (PMC11185459; doi:10.1371/journal.pcbi.1011912)
Supplement: S5 File — (ZIP) [file pcbi.1011912.s007.zip › metDataModel-master/docs/DataModel-illustration-202401-SL.pptx]

## Slide 1
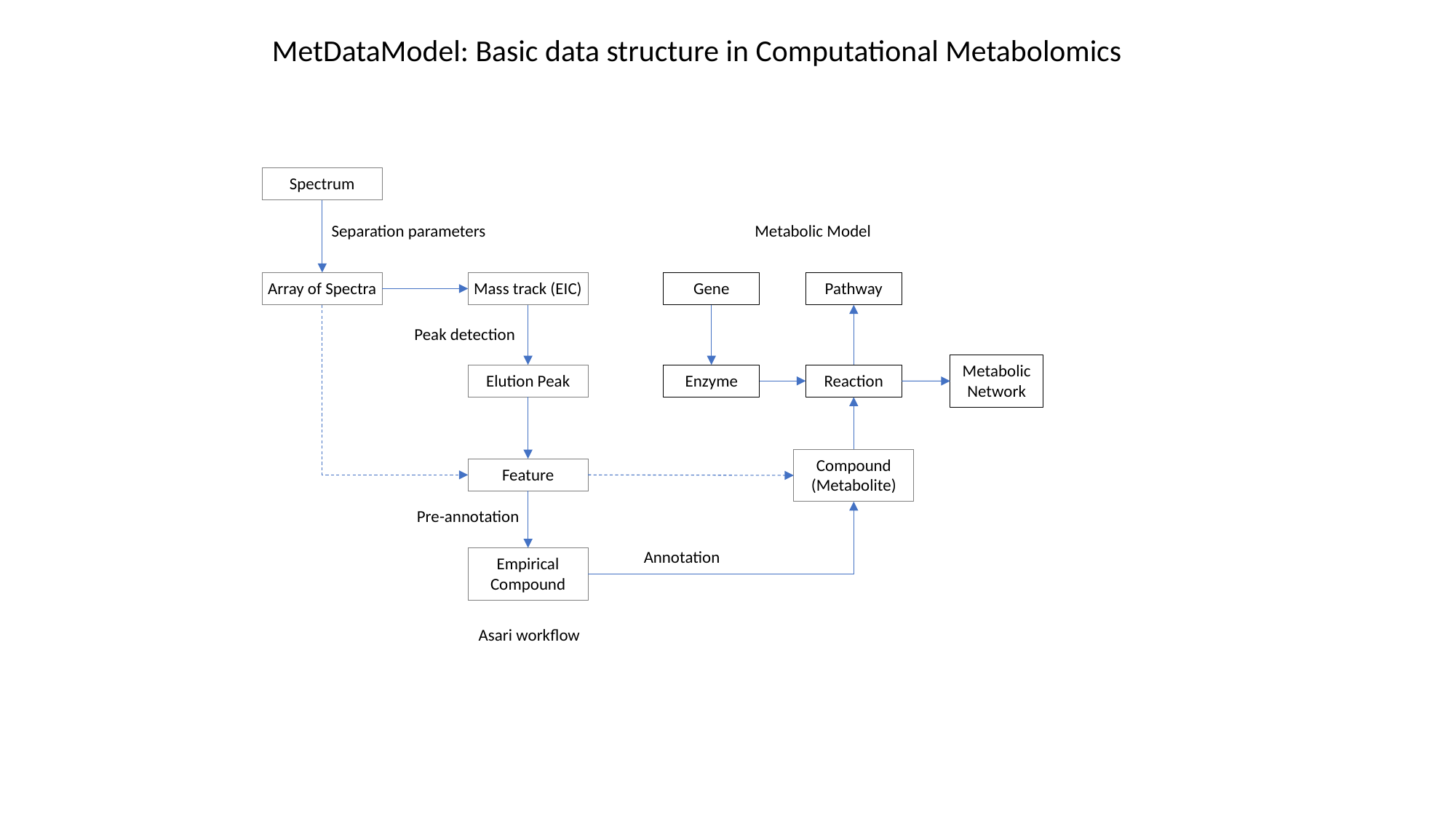

MetDataModel: Basic data structure in Computational Metabolomics
Spectrum
Separation parameters
Metabolic Model
Array of Spectra
Mass track (EIC)
Gene
Pathway
Peak detection
Metabolic
Network
Elution Peak
Enzyme
Reaction
Compound
(Metabolite)
Feature
Pre-annotation
Annotation
Empirical
Compound
Asari workflow

## Slide 2
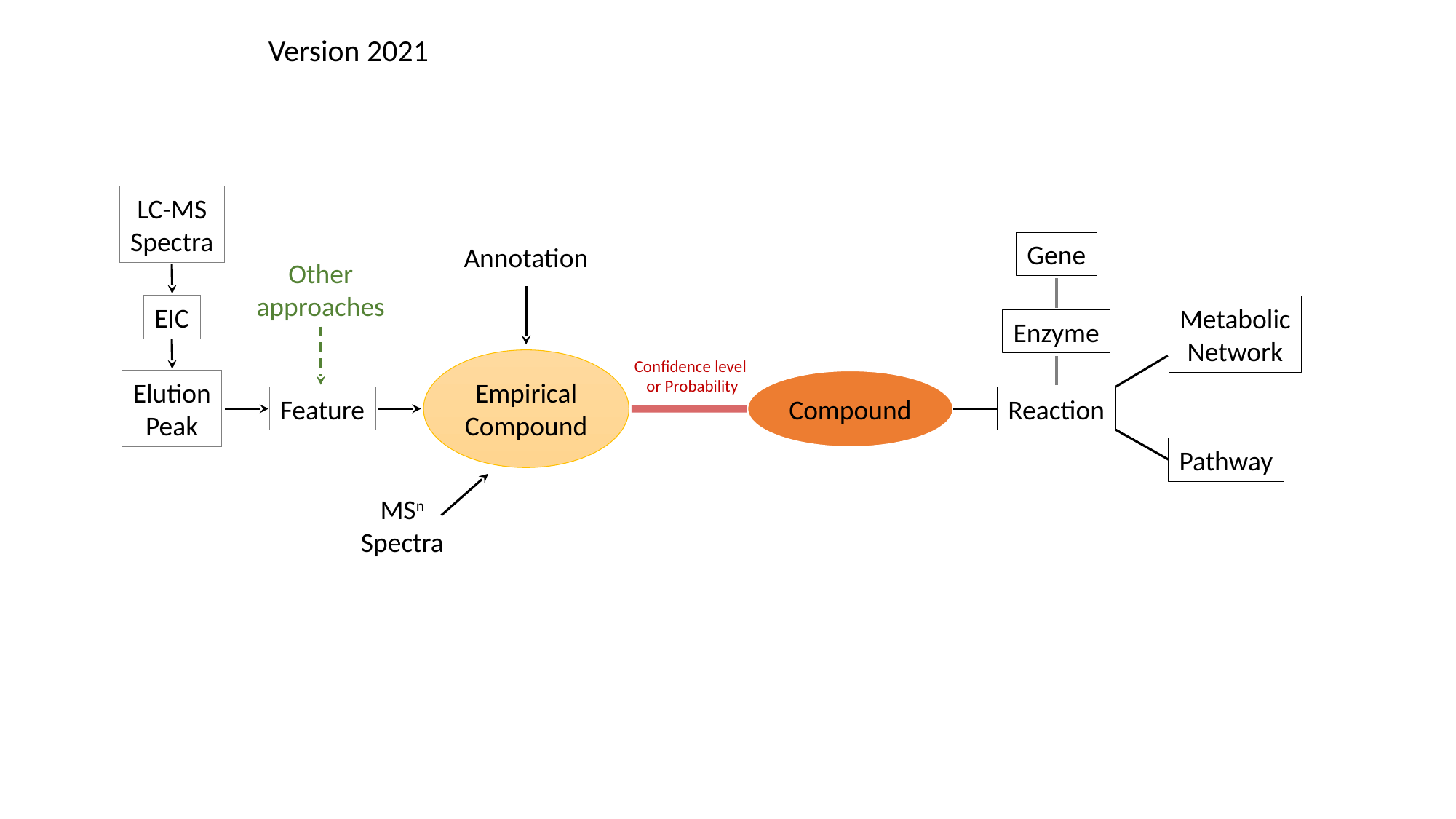

Version 2021
LC-MS
Spectra
Gene
Annotation
Other
approaches
EIC
Metabolic
Network
Enzyme
Empirical
Compound
Confidence level
or Probability
Elution
Peak
Compound
Feature
Reaction
Pathway
MSn
Spectra

## Slide 3
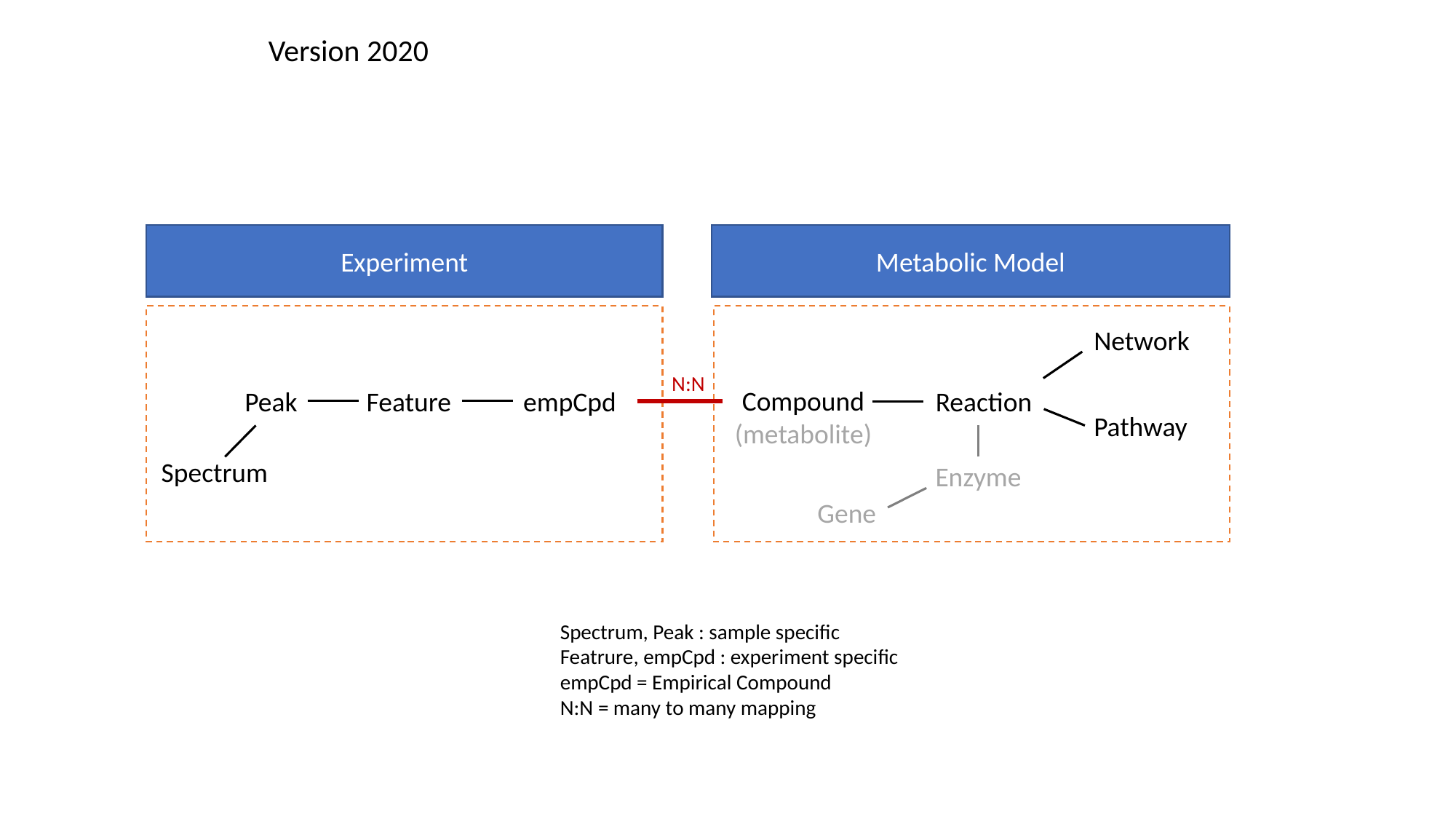

Version 2020
Experiment
Metabolic Model
Network
N:N
Compound
(metabolite)
Peak
Feature
empCpd
Reaction
Pathway
Spectrum
Enzyme
Gene
Spectrum, Peak : sample specific
Featrure, empCpd : experiment specific
empCpd = Empirical Compound
N:N = many to many mapping
