# Supplementary figures and images for: Common data models to streamline metabolomics processing and annotation, and implementation in a Python pipeline
Source: PLoS Comput Biol. 2024 Jun 6;20(6):e1011912. doi: 10.1371/journal.pcbi.1011912 (PMC11185459; doi:10.1371/journal.pcbi.1011912)

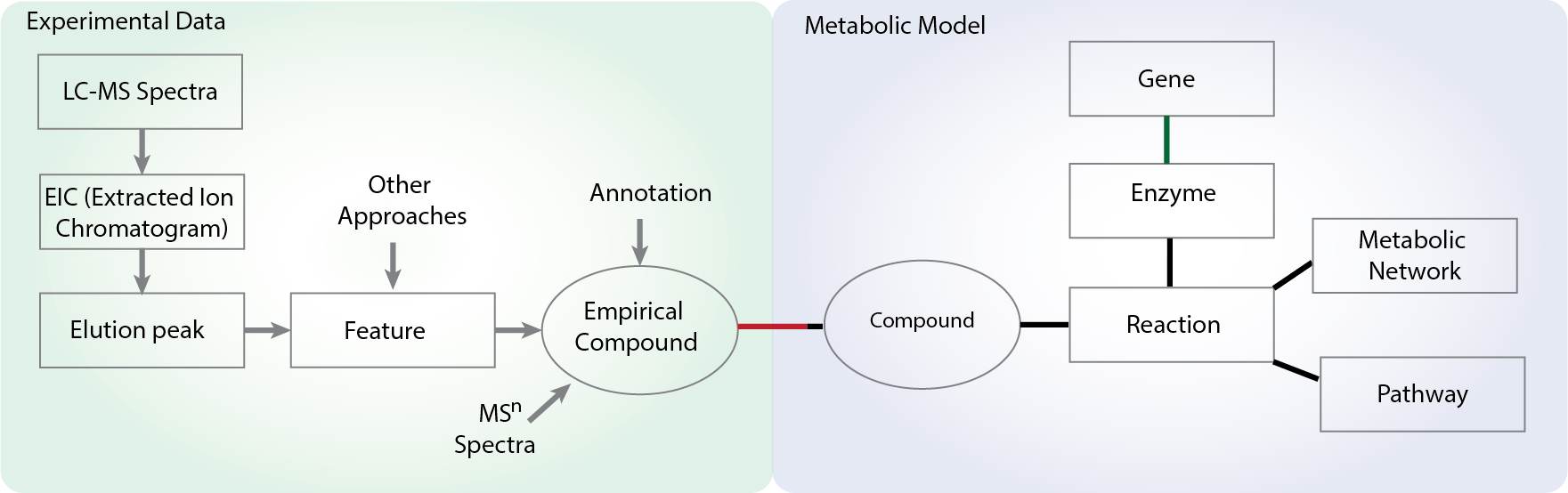

Supplement: S5 File — (ZIP) [file pcbi.1011912.s007.zip › metDataModel-master/docs/datastru.png]

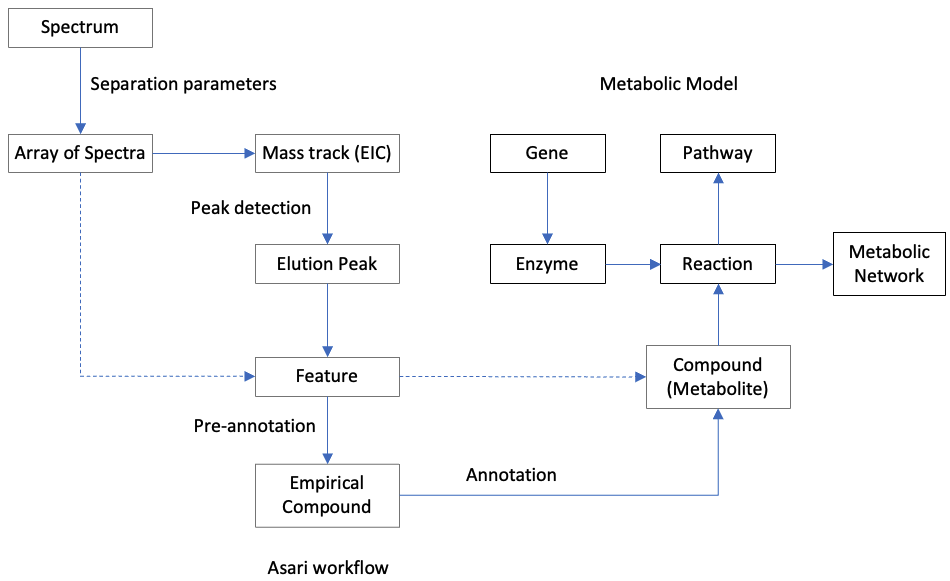

Supplement: S5 File — (ZIP) [file pcbi.1011912.s007.zip › metDataModel-master/docs/datastru2024.png]
