# Supplementary material for: Common data models to streamline metabolomics processing and annotation, and implementation in a Python pipeline
Source: PLoS Comput Biol. 2024 Jun 6;20(6):e1011912. doi: 10.1371/journal.pcbi.1011912 (PMC11185459; doi:10.1371/journal.pcbi.1011912)
Supplement: Attachment — Submitted filename: pcpfm_response_document_0501-SL.docx [file pcbi.1011912.s008.docx]

We thank the reviewers for their comments. All the issues raised by the reviewers are addressed below.

We have updated the manuscript, figures 2B and C, figure 3D, figure 4B-E, and figures 5 D-F and both the pcpfm and metDataModel software packages in response to these comments.

Given the software update and the community adaptation (two workshops have been held), we wish to make the results here to be consistent with the newest software version and have regenerated the results. This update has no significant impact on the results or the conclusions of the manuscript. The HZV029 Lipidomics dataset has been renamed to "HZV029 Two-Phase HILIC", which is a more correct description and the method for its generation was corrected.

All figures have been checked by PACE. Data availability and reproducibility have been described the same as the previous submission via data and code repositories.

The reviewer comments are numbered in blue, and our responses are in black (line numbers refer to the revised clean version).

**Reviewer 1**

1. I would suggest the authors provide more detailed descriptions of each step in their pipeline. For example, how does the pipeline detect and remove outliers? What QA/QC measures are implemented? What missing value imputation approaches are used? In addition, the authors should clarify their contributions by specifying whether they implemented the algorithms, e.g., t-SNE themselves or used a function from (which?) existing Python packages but wrapped for LC-MS data.

This has been addressed in the main text by providing more details enumerating every metric that is provided and what libraries they employ (Lines 166-192). In addition, a new section of workflow description is added to the end of Supplemental File S1 (Lines 228-277) that both summarizes the default parameters used by each operation but also the details of each workflow used in the manuscript and where they deviate from the defaults and why.

For the specific questions, outlier detection is based on feature count using a Z-score cutoff of 2.5 relative to the median number of features but can use any QA/QC result that is 1-dimensional (i.e., not PCA, t-SNE or raw correlation values). The QA/QC metrics include PCA (with and without log transformation of feature intensities before scaling), t-SNE, correlation clustermaps (spearman, kendall-tau, pearson), median correlation expressed using Z-scores, raw and log TICs, and mean and median feature intensity plots. Most operations are performed using numpy, scipy and pandas via custom routines, however machine learning approaches including PCA and t-SNE use scikit-learn, while clustermaps use seaborn. Currently only imputation with a scalar multiple of the minimum value for the feature is implemented, but more options for each operation will be implemented in the future. The above information is now in the manuscript.

2. The authors should provide a clear justification for their workflow. For instance, explaining why batch correction is done after missing value imputation. What "various statistical tests" are used, and how do they "quantify sample properties"?

The pcpfm workflow is designed to be flexible, where end users can tailor it to their specific experiments. No parameters are hard coded. The default workflow has been utilized in our own biomedical projects and is provided here as an example.

A specific order of steps is recommended for blank masking, normalization, removal of outliers, feature imputation and filtering, because each step removes bias for the next step. However, should an end-user wish to omit a step, change orders or parameters, they can do so with ease. The batch correction step is sensitive to missing values, but it can be done before (by omitting features with many missing values) or after missing value imputation. The "various statistical tests" are described in the response to issue 1. Z-scores are used to "quantify sample properties".

These above points have been added to the manuscript (Lines 183-189). To further clarify the flexible sequence, we have modified figure 2 to remove any ordering of the steps that is not strictly necessary and added this explicitly to the manuscript.

3. What does the author mean by saying "Some operations can be made batch aware ..." and "These operations are implemented using a mixture of sklearn (please refer to the package as scikit-learn) ..."?

The pipeline can perform many operations in a batch-aware fashion meaning that the operations are performed in a two-step process where they are first applied within a batch and then across the entire dataframe after the batch-level operation is completed. This is useful for normalization as it first normalizes per-batch effects then corrects the batches to one another. Other procedures in the pipeline such as missing value imputation and blank masking can also be performed in this manner, although this is likely to exacerbate or possibly introduce batch effects. As such, we have clarified the main text to describe the two-pass normalization only.

This has been clarified in the main text (Lines 179-182).

The package is now correctly referred to as scikit-learn in the main text.

4. The author should clearly explain the outputs of the pipeline. For example, what are the contents of the "PDF reports"? What results from each step in Fig 2 will be included

We have clarified in the main text that the contents of the PDF reports are controlled by a user-provided JSON file. There are examples in the main repository and example reports are provided as Supplemental Files S2 and S3. We have also enumerated the default contents of these reports in the main text (Lines 200-205). While they do not include every step shown if figure 2, PCA, TICs, pearson correlation clustermaps, missing value Z-score plots are shown for each feature table and a tabular summary of the explained features, and their annotations is provided for each empirical compound.

5. The url of the MetDataModel GitHub repo is correct but the link is incorrect.

If this is in reference to the “link” provided on line 257 of the pdf version of the manuscript, this error appears to be the result of the software used to open the pdf inferring the presence of a link but not accounting for the line break between lines 257 and 258. In the submitted version of the manuscript, there was no hyperlink provided, just the URL. Thus, this is something that should be rectified during proofing. We have confirmed that the provided URL is correct.

**Reviewer 2**

1. - (line 193) There is no reference information for the four studies mentioned. This is necessary for the reader to be able to compare previous results with those from the presented software.

These datasets were described in detail in Supplemental File S1 and references to each public dataset’s corresponding publication were provided when they were discussed (lines 233, 242 and 245).

We have added references to line 218 for the sake of clarity.

2. - (line 199) There is no methodological description of how the test was conducted. How is the reader meant to believe that the performance comparison to XCMS is fair/valid/informative?

Detailed versions of packages are now added to the manuscript lines 227-230 with additional details in Supplemental File S1, lines 230-238. Since these tools are open, anyone can reproduce the results with provided data.

The performance delta observed here agrees with previous comparisons of asari to other common pre-processing tools (Nature Communications 14.1 (2023): 4113). We have clarified that the comparison between the pcpfm and XCMS for this test consists of the pre-annotation and pre-processing steps only as this represents the minimal processing necessary to get an interpretable feature table for downstream analysis.

3. - (line 200) It is not adequately explained what Figure 2C is depicting (e.g. what is N?).

This was an oversight in the figure legend, N represents the number of MS^1^-only acquisitions in both Figure 2B and 2C. This has been clarified in the revised version (Figure 2 caption, Lines 545 and 553-554).

4. - (lines 195-200) The discussion of results is lacking important details, such as how many features are detected from either software, how many are annotated, what level of confidence are the features annotated with?

The numbers of features and annotations are now added to the Figure 2 caption (Lines 548-551 and 554-558). Since Figure 2C focuses on computational performance and annotation is the focus of Figure 3, we have limited Figure 2C to preannotation. The direct comparison between the annotation results was omitted intentionally here for two reasons. First, for computational limitations processing the entire HZV029 dataset with MetaboAnalystR is impractical (too slow) and since AcquireX was used for generating data for annotation the more apt comparison is to compare HZV029 results against Compound Discoverer, as this is the closest to ground truth as possible for this dataset, which was done later in the manuscript. For the Checkmate Subset, where we have ground truth annotations via the library and a small enough sample size for both tools to process them, we compare those results later in the manuscript as well.

5. - (lines 214-220) There is no appreciable description or discussion of the methodology or results presented.

The methods used to generate the MS^2^ annotations for both the CD and pcpfm were provided in supplemental. In short, we constructed “annotated feature sets” which were sets of strings created by concatenating the annotation name with the mapped feature’s ID. These sets were then compared using the set logic operators in python to evaluate their similarity. We have added the additional detail in the main text (Lines 244-252) and Supplemental File 1 (Lines 170-205) regarding the implementation of the comparison.

6. - (lines 221-231) There is no appreciable description or discussion of the methodology or results presented. This section seems to highlight the conclusions without describing the results themselves or how those results support the derived conclusions.

We have added (Lines 256-268) to the main text to describe how the results support conclusions. In addition, description regarding the workflows used to generate the results is added in Supplemental File 1 (Lines 239-277). The workflows and notebooks for the results are also available in the code repository.

7. - My feedback for the results sections referring to Figure 5 are likewise to the comments above

We have added Lines 277-280, 283-290, 291-299, and 302 to the main text to describe how the results support conclusions. In addition, description regarding the workflows used to generate the results is added in Supplemental File 1 (Lines 239-276) The workflows and notebooks for the results are also available in the code repository.

Minor Points:
8. - (line 15) Typo in abstract: “… it is essential is have a set …”

Corrected, this should have said ‘is essential **to** have a set’.

9. - In my view, the implementation of the MetDataModel classes would benefit from two changes: 1) use of dataclasses and 2) use of type annotations. Dataclasses (see PEP 557) are a better construct for the intended use case as I understand it. Semantically they convey the intent of the defined objects as means of storing state with minimal behavior. Syntactically they greatly simplify the class definitions since only the desired attributes (with default values where applicable) and methods (seems like serialize is the main one) must be explicitly defined while boilerplate methods like __init__, __str__, __repr__, etc. are defined for you. Dynamic typing is a convenient feature of Python, but the reality is that there are also many other languages in common use which conform to more strong typing conventions. With the stated goal of defining these data structures in a way that is language agnostic and generalizable, I feel that the inclusion of type annotations for the attributes of the MetDataModels classes is a must in order to avoid ambiguity for those who wish to adapt the proposed data model into applications written in strongly typed languages.

Thanks for this interesting suggestion. We have now added a dataclass-based implementation with type annotations of the metDataModel to the repository, which is used by pcpfm and related packages. The previous implementation is moved to the “simple” model as an alternative.

**Reviewer 3**

1. Although I agree that MetaboAnalyst is the most popular and convenient software tool in metabolomics, you need to provide a comparison of functionalities with some other software based on R and with or without GUI.
   First of all there are patRoon (doi: 10.1186/s13321-020-00477-w ; <https://github.com/rickhelmus/patRoon>) and xcmsrocker/rmwf (doi: 10.1186/s13321-022-00586-8 ; <https://github.com/yufree/xcmsrocker> ; <https://github.com/yufree/rmwf>). They mainly focus on comprehensive raw data processing, peak detection and annotation, including MS2.
   Also OUKS provides an extensive variety of options for peak table processing such as: correction, normalization, imputation, statistical analysis and QA/QC (doi: 10.1021/acs.jproteome.1c00392; <https://github.com/plyush1993/OUKS>).
   Add comparison with GUI tools such as Web-version of MetaboAnalyst, Workflow4Metabolomics (doi: 10.1016/j.biocel.2017.07.002 ; <https://workflow4metabolomics.org/>) and IP4M (doi: 10.1186/s12859-020-03786-x).

Thanks for this suggestion. The xcmsrocker/rmwf repos are actually maintained by a colleague in our team. A new Supplemental Table 2 is added to summarize the relative capabilities and features of the pcpfm to the tools you listed above. In addition, we have included TidyMass (Shen et al. "TidyMass an object-oriented reproducible analysis framework for LC–MS data." Nature Communications 13.1 (2022): 4365), which is in the same category of XCMS/R based tools. References to these tools are added to the main manuscript (Lines 214-216).
